# Biosynthesis and characterization of silver nanoparticles from *Asplenium dalhousiae* and their potential biological properties

**DOI:** 10.1371/journal.pone.0325533

**Published:** 2025-06-30

**Authors:** Shafia Parveen, Shazia Iqbal, Saima Maher, Paul Wilson, Habib Nasir, Samreen Soomro, Shazia Nisar, Muhammad Imran, Musarat Riaz, Rabail Urooj, Mah Ganj Bakhshi, Maria Essa, Farah Mukhtar, Attya Bhatti, Hussnain A. Janjua, Amir Faisal, Arsalan Saleem

**Affiliations:** 1 Department of Chemistry, Sardar Bahadur Khan Women University Quetta, Quetta, Pakistan; 2 Department of Chemistry, Balochistan University of Information Technology, Engineering and Management Sciences, Quetta, Pakistan; 3 Department of Chemistry, Warwick University, Coventry, United Kingdom; 4 Department of Chemistry, School of Natural Sciences, National University of Sciences and Technology, Islamabad, Pakistan; 5 Department of Pharmaceutics, Faculty of Pharmacy, Northern Border University, Rafha, Saudi Arabia; 6 Department of Chemistry, University of Karachi, Karachi, Pakistan; 7 Department of Chemistry, Faculty of Science, King Khalid University, Abha, Saudi Arabia; 8 Department of Environmental Science, Sardar Bahadur Khan Women University Quetta, Quetta, Pakistan; 9 Department of Biomedicine, Atta-ur-Rahman School of Applied Biosciences (ASAB), National University of Sciences and Technology (NUST), Islamabad, Pakistan; 10 Department of Industrial Biotechnology, Atta-ur-Rahman School of Applied Biosciences (ASAB), National University of Sciences and Technology (NUST), Islamabad, Pakistan; 11 Department of Biology, Syed Babar Ali School of Science and Engineering, Lahore University of Management Sciences, Lahore, Pakistan; 12 Department of Biology, Georgia State University, Atlanta, Georgia, United States of America; SRM Institute of Science and Technology, INDIA

## Abstract

This study investigated the green synthesis of silver nanoparticles (AgNPs) using the medicinal plant *Asplenium dalhousiae* focusing on its bioactive chemical constituents as natural reducing agents. Aqueous, chloroform, and *n*-hexane extracts of the plant leaves were utilized in the nanoparticle synthesis process. The synthesized AgNPs were confirmed through UV-visible spectroscopy, showing absorption peaks at approximately 420 nm, 443 nm, and 439 nm. Fourier-transform infrared (FTIR) spectroscopy was used to recognize the functional groups in the plant extracts responsible for facilitating the reduction process. The morphological and structural characteristics of the synthesized nanoparticles were analyzed using Scanning Electron Microscopy (SEM) and X-ray Diffraction (XRD). These analyses revealed that the nanoparticles synthesized using the Aqueous, chloroform, and n-hexane extracts were predominantly spherical silver nanoparticles (AgNPs) with a crystalline structure and an average diameter of 46.98 ± 12.45 nm, as determined by SEM. The antibacterial efficacy of the synthesized AgNPs was evaluated against *Escherichia coli*, *Bacillus subtilis*, and *Pseudomonas aeruginosa* at a concentration of 30 μg/ml. Among the tested nanoparticles, the AgNPs synthesized from the *n*-hexane extract exhibited the highest antibacterial activity, with zones of inhibition measuring 20.0 ± 1.8 mm for *E. coli*, 19.0 ± 1.2 mm for *B. subtilis*, and 19.5 ± 1.4 mm for *P. aeruginosa*. Additionally, the silver nanoparticles (AgNPs) from *Asplenium dalhousiae* demonstrated significant α-amylase inhibition, with 85.04% inhibition at 500 µg/ml, compared to Acarbose (90.84%) and the leaf extract (78.65%). The antioxidant activity of the synthesized AgNPs was assessed using the DPPH method, which confirmed their significant antioxidant properties alongside their antibacterial activity. The aqueous and n-hexane silver nanoparticles (AgNPs) showed strong cytotoxic activity with low IC_50_ values, particularly in A2780 cells (15.76 µg/ml and 9.11 µg/ml, respectively), while the plant methanolic extract and CHCl_3_ AgNPs exhibited much higher IC_50_ values, indicating moderate to low activity. This study highlights the potential of AgNPs in handling anticancer, α-amylase, and antibacterial infections. By assimilating natural products with nanotechnology, it deals an inventive approach to developing targeted, eco-friendly therapies, paving the way for cutting-edge biomedical applications and improved treatment outcomes.

## Introduction

Nanotechnology, a rapidly advancing interdisciplinary field including chemistry, biomedical science, material science, diagnostics, therapeutics, and medicine, comprises the production and application of nanoscale materials with various properties [1]. Its key significance is highlighted by its broad uses, predominantly catalyzing organic toxin reduction in environmental perceptions [2–4]. Furthermore, nanotechnologies have vital biological properties, such as anti-bacterial [5,6], Anti-oxidant [7,8], and anticancer activities [9]. Many inorganic metallic elements, comprising iron, copper, zinc, gold, silver, magnesium, and titanium, have been utilized in the biological, physical, and chemical synthesis of nanomaterials for a several determinations. Chemical methods are involved the use of harmful chemicals and energy intensive procedures, raising concerns about their environmental effect and the formation of toxic by-products. In compare, green synthesis methods deal a more sustainable resolution by utilizing bio-based materials, such as microorganisms, plants, as eco-friendly sources for nanoparticle synthesis. Many studies have confirmed that green synthesis not only reduces environmental harm but also effectively produces nanoparticles with desirable characteristics. Silver nanoparticles (AgNPs) play an important role in advanced research due to their nano medicinal prospective [10,11]. The green method in nanotechnology presents several advantages, encompassing cost-effectiveness, environmental affability, high yield, and precipitate synthesis [12]. In this method, the synthesis of silver nanoparticles (AgNPs) apply extracts from various plant parts [13–17]. AgNPs are known for their biocompatibility and are important in the medical and pharmaceutical domains [18]. such as antibacterial activities [19,20], antioxidant qualities [21,22], immunological advantages [23], and anticancer activity, have been identified by researchers. Silver nanoparticles (AgNPs) have recently emerged as promising candidates for the development of novel anticancer drugs due to their broad spectrum of biological importance [9–11]. It exhibits potential cytotoxic effects on cancerous cells, which can be recognized to their exceptional physicochemical properties. The promising use of nanoparticles as therapeutic agents for focused cancer detection and controlling is rapidly gaining traction. Specially, AgNPs have concerned significant attention for their anticancer properties, comprising their capability to affect with signaling pathways dangerous for cancer cell growth and metastasis. literature suggest that AgNPs can induce cytotoxic effects on malignant cells, thereby inhibiting tumor growth and progression [24,25]. In addition to their anticancer potential, medicinal plants have long been recognized for their therapeutic benefits, particularly in regions like Pakistan, where traditional medicine relies heavily on plant-based remedies [26]. The adverse effects associated with synthetic pharmaceuticals, such as hypertension, hormonal imbalances, and weight gain, have further driven the resurgence in the use of natural products for medicinal purposes. A systematic investigation and further studies are required to recognize the mechanisms underlying these special effects and to confirm their efficacy with robust scientific confirmation [26]. A current study focused on the efficient exploration of *Asplenium dalhousiae*, a well-known medicinal plant. This research represents the first ever synthesis of silver nanoparticles using this plant species. The flowering period of *Asplenium dalhousiae* spans from July to December, thriving in moist environments typical of temperate and subtropical regions []. Compounds isolated from *Asplenium dalhousiae* comprise flavonoids, phenols, glycosides, alkaloids, coumarins, anthraquinones, quinones, cardiac glycosides, terpenoids, and saponins. The plant is particularly rich in phenolic and flavonoid as a major component, which contribute to its antioxidant activities [26–31]. The study developed extracts from *Asplenium dalhousiae* to synthesize AgNPs and subsequently evaluated their antibacterial and antioxidant properties, α-amylase enzyme assay, Anticancer activity [32–35]. Despite the plant’s recognized biological significance, its full potential remains underexplored. Previous reported data have proved its antibacterial and antifungal activities, as well as antifertility properties observed in adult male rats. A comprehensive investigation of *Asplenium dalhousiae*, aiming on its potential for silver nanoparticle (AgNP) synthesis and the bioactivities of these nanoparticles. While several studies have discovered plant mediated AgNP synthesis, comprising research on other Asplenium species, this is the first study to explore the use of *Asplenium dalhousiae* in this context. Additionally, our study is the first to assess the bioactivity of the AgNPs synthesized from *Asplenium dalhousiae* in terms of α-amylase inhibition and anticancer effects, specifically against colorectal (HCT116) and ovarian (A2780) cancer cell lines. Our findings contribute to the growing body of knowledge on plant mediated nanomaterials and highlight the new application of *Asplenium dalhousiae* for nanotechnology based therapeutic interventions.

## Materials and methods

### Sample collection, identification and analysis

The selected plant for this research, *Asplenium dalhousiae,* was collected from the Valley of Channat Bagh site in Azad Kashmir. Taxonomic identification was performed by Dr. Amir from NARC, Islamabad. The voucher specimen has been deposited in the Botany Department Herbarium under the code CHEM-311DX. The collected plant material was washed with distill water and then shade-drying, followed by uniform grinding the powder material was kept in refrigerator for further procedure.

### Preparation of plant extracts *(Asplenium dalhousiae)*

**Fig 1:**
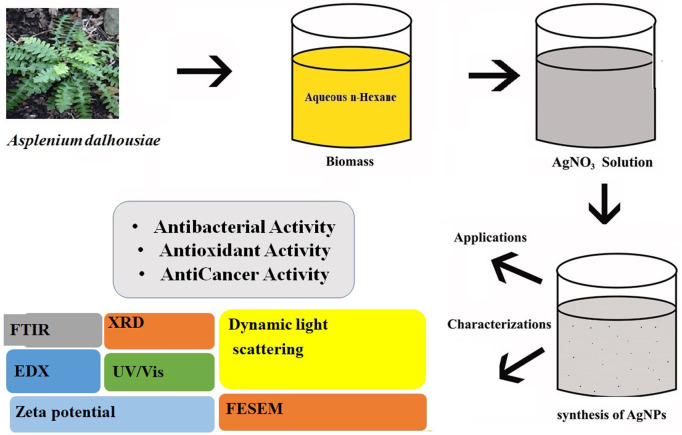
Graphical abstract illustrating the green synthesis of silver nanoparticles (AgNPs) using Asplenium dalhousiae leaf extracts and their evaluation for antibacterial, antioxidant, α‐amylase inhibitory, and anticancer activities.

The 500g of powdered dried plant material soaked in 100% methanol for 15 days at room temperature (approximately 25°C) [30]. The procedure was followed by filtration with help of 125 mm pore size Whatman filter paper. The filtrate was placed in rotatory evaporator to evaporate at 45°C [33]. The gummy crude extract was dissolved and suspended in distilled water (140 ml) and successively portioned with different organic solvent such as *n-*Hexane (3 × 200 ml), CHCl_3_ (3 × 200 ml) and a water layer (3 × 200 ml), (Fig 1).

### Synthesis of silver nanoparticle

1 mM silver nitrate solution and the plant aqueous, chloroform, and *n*-hexane extracts solution (1:1) were mixed on a hot plate with continuous stirring at 62°C and pH 11.72 until a color change indicated the synthesis of AgNPs [31]. The reaction progress was constantly monitored using a UV-visible spectrophotometer to record the SPR peak.

## Characterization of silver nanoparticles

### UV-Vis spectroscopy

UV-Vis spectroscopy plays an important role for examining the reduction of silver ions to silver nanoparticles. The UV spectra of *Asplenium dalhousiae* plant extract were taken at different concentrations the range was 250 mL to 650 mL in 10 mL AgNO_3_ solution. UV-2800, BMS model was used in this research. The spectra of AgNPs were recorded between 300 and 800 nm. The UV-Vis spectrum demonstrates the surface Plasmon resonance (SPR) absorption band of nanoparticles, showing a gradual decrease in absorbance with a shift in wavelength and changes in band width. This finding underscores the significance of the reduction of silver ions to silver nanoparticles and characterizing the silver optical properties of silver nanoparticles and structural features.

### Fourier Transform Infrared (FTIR)

Fourier Transform Infrared (FTIR), is used to recognize the functional group existent in plant. This technique, highlighting the role of bioactive plants chemicals in reduction and capping processes. The IR spectra were recorded with support of Perklin Elmer spectrum 100 models.

### X-ray diffraction

X-ray diffraction is a very advantageous technique to determine the crystalline nature structure and characterization of synthesized nanoparticles through X-ray diffractogram [36]. The instrumental model was used XRD; D8 Advance; Bruker, Billerica, MA, USA.

### Scanning electron microscopy (SEM)

Scanning Electron Microscopy (SEM), the morphology and size of synthesized silver nanoparticles (Ag-NPs) were examined using the JEOL-6490A-JSM (SEM).

### Energy dispersion X-ray (EDX)

Energy dispersion X-ray (EDX) was employed to acquire elemental composition of synthesized silver nanoparticles.

### Preparation of *Asplenium dalhousiae* extract and AgNPs

The plant extracts were prepared using *n*-hexane, chloroform, and distilled water as solvents. For the green synthesis of silver nanoparticles (AgNPs), each dried extract (10 mg) was dissolved in 10 mL of sterile distilled water. A 1 mM aqueous solution of silver nitrate (AgNO₃) was then added to each plant extract solution to make a final reaction volume of 100 mL. The mixtures were incubated at room temperature, and the formation of AgNPs was indicated by a visible color change. The resulting solutions were then centrifuged at 4,000 rpm for 25 minutes to separate the synthesized silver nanoparticles. The supernatants were discarded, and the nanoparticle pellets were collected and stored for further characterization.

### Antibacterial activity assay

The collected obtained pellets were used. The disc diffusion method was applied for antibacterial activity [22,34]. Amoxicillin+Clavuanic acid was used as standard solution. The nutrient agar was prepared by suspending 28.0 grams in 1000 ml of distilled water and sterilizing it by autoclaving at 15 Ibs pressure (121^°^C) for 15 minutes. The agar was then mixed well and poured into prepared sterile petri plates. *Asplenium dalhousiae* leaves extracts and its synthesized silver nanoparticles were studied [34]. The Gram-positive bacteria and Gram-negative bacteria strains were used in the biological assays. Nutrient agar was spread on sterilizes petri plates suspended in about 10 ml of physiological saline in a Roux bottle and was incubated at 37°C for 24 hours. The preparation of antibiotic sensitivity discs involves using Whatman No. 1 filter paper to create discs approximately 1 mm in diameter. These discs are then sterilized by placing them in hot air. After sterilization, each disc is loaded with 30 μl of the sample extract, silver nanoparticles (AgNPs), and a standard solution such as Amoxicillin+Clavuanic acid (30 mg/ml distilled water). Subsequently, the loaded discs are refrigerated for 24 hours before being used for experiments [35].

### Microorganisms

The antimicrobial assay of Ag nanoparticles was examined against The following bacteria were tested the Gram-negative *Escherichia coli* (ATCC 25922) and *Pseudomonas aeruginosa* (ATCC 27853), as well as the Gram-positive *Bacillus subtilis* (ATCC 13048). Nutrient agar (NA) was used for bacterial growth. The agar plates were dried for 10 minutes. The selected bacterial strains were inoculated in nutrient broth to facilitate the growth of microorganisms.

### Evaluation of anti-oxidant activity by DPPH assay

The antioxidant assay of plant three extracts a (*n-*hexane, chloroform and aqueous, extracts) and synthesized nanoparticles were evaluated by methanolic DPPH assay [37]. This assay is constructed on the reduction of a DPPH in the presence of a reducing agent or antioxidant, the result of this reaction is the development of a stable non-radical DPPH-H molecule. The AgNPs solution was prepared in methanol at different concentrations the range were 10–100 µg/ml, and 200µl from all dilution of silver nanoparticle solutions were added to 0.8 µl of DPPH solution. The samples were incubated in the dark for 30 minutes at room temperature, and the absorbance of each solution was read at 517 nm. Ascorbic acid was used as a positive control, and the antioxidant capacity to scavenge the DPPH radical was calculated as a percentage using the formula:


DPPH scavenging capacity (%) = [(A0 – A1) / A0] × 100 

Where A0 is the absorbance of the control.

A1 is the absorbance of the sample [1].

### In-vitro α-amylase enzyme assay

Alpha amylase inhibitors attach to the alpha bonds of polysaccharides like glycogen and starch, preventing their breakdown into glucose and maltose. These inhibitors play a vital role in several applications, and management human diseases. They work by reducing endogenous alpha amylase activity, thereby impeding the conversion of complex carbohydrates and disaccharides into absorbable monosaccharides. Clinical trials have shown that these inhibitors delay the digestion of complex carbohydrates and disaccharides, inhibiting α-glucosidases and impacting the absorption of glucose. Polyphenols found in dietary sources act as natural inhibitors of α-amylase and α-glucosidase, slowing down glucose absorption by interfering with these key enzymes involved in carbohydrate digestion. Numerous folkloric and medicinal plant extracts have demonstrated significant α-amylase inhibition activity, although additional animal studies are required to validate their hypoglycemic effects. Previous research has explored the role of medicinal plants in α-amylase enzyme inhibition such as, *Prosopis cineraria* (L.), *Terfezia claveryi*, *Chenopodium album* L., and *Salvia lavandulifolia Vahl* show the highest potential for α-amylase inhibition [32].

### Anticancer activity

Anticancer activity states to the capability of a compound to inhibit the growth and propagation of cancer cells. To estimate this activity against colorectal (HCT116) and ovarian (A2780) cancer cell lines, the cancer cell lines (HCT116 and A2780) are cultured in media DMEM or RPMI-1640, added with 1% penicillin-streptomycin and 10% fetal bovine serum (FBS). The cells are kept in a moistened incubator at 36°C with 5% CO₂ [38]. The test components, silver nanoparticles (AgNPs), and plants extracts were dissolved in a DMSO solvent and diluted in the culture media to prepare different concentrations.

Cancer cells are placed into 96-well plates at a density of 5,000–10,000 cells per well and left overnight to attach to the surface. The cells are then treated with different concentrations of the test compound for 24, 48, and 72 hours. After treatment, cell viability is assessed using methods such as the MTT or CCK-8 assay. These assays measure metabolic activity by detecting color or fluorescence changes, which are proportional to the number of viable cells. Apoptosis is assessed using flow cytometry with annexin V-FITC and propidium iodide (PI) staining, while cell cycle analysis is performed by examining DNA content through PI staining. Morphological changes, such as cell shrinkage and chromatin condensation, are observed under a microscope to confirm apoptotic activity [39,40]. Results are expressed as IC₅₀ values, which indicate the concentration of the compound needed to inhibit 50% of cell growth. These values are statistically analyzed to compare treated cells with controls. Compounds demonstrating significant inhibition of cell growth, induction of apoptosis, or cell cycle stop against HCT116 and A2780 cell lines may have potential therapeutic implications for colorectal and ovarian cancer treatment [41–44]. The statistical significance of these results was assessed using one-way ANOVA followed by Duncan’s multiple range test, as previously described by Harvey and Paige [45,37].

### Statistical analysis

The statistical analysis involved expressing the results as Mean ± SD it is used to determine significant differences in each samples, a one-way analysis of variance (ANOVA) was conducted, followed by the Duncan’s test for multiple comparisons, as outlined by Harvey and Paige (1998) [45,37]. The statistical analysis was carried out using Graph Pad Prism Software, version 5. A significance level of p < 0.05 was considered, indicating that results with a p-value below this threshold were deemed statistically significant. This approach provided a comprehensive evaluation of the antibacterial activity of Ag-NPs and their effectiveness against different bacterial strains

## Results

### Synthesis of AgNPs

In the current study, (S1 Fig) the biosynthesis of silver nanoparticles (AgNPs) was conducted under laboratory conditions using *Asplenium dalhousiae* (S2 Fig) plant extract, with three different fractions. The synthesis of silver nanoparticles (AgNPs) was confirmed by a visible color change in the reaction medium from yellow to orange-brown.

### UV-Vis spectroscopy

**Fig 2:**
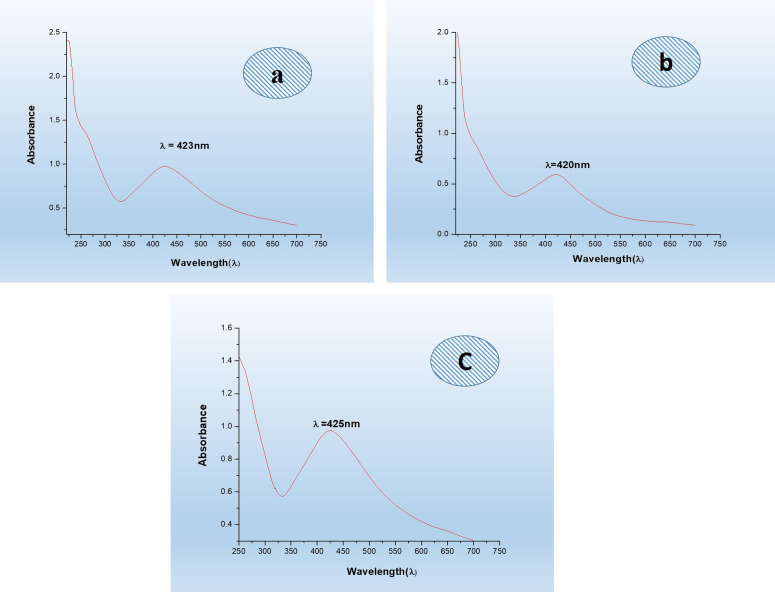
UV-Vis spectra of *Asplenium dalhousiae* plant extract. a) aqueous b) chloroform c) n-hexane extracts.

The green synthesis of silver nanoparticles using the plant extract of *Asplenium dalhousiae* resulted in a color change from pale yellow to orange-brown, representing the formation of silver nanoparticles the UV-Vis results showing absorption peaks at 423 nm (aqueous extract), (Fig 2a) 420 nm (chloroform extract), (Fig 2b) and 425 nm (*n*-hexane extract), (Fig 2c). These absorption peaks support with previous reported data [33], of silver nanoparticles at 400 nm. Particularly, the broadening of the peak detected in spectra of the *n-*hexane as the higher concentration of silver nanoparticles it was compared with the other two extracts.

### Fourier transforms infrared spectroscopy

Fourier transformed infrared spectroscopy is used to identified the function group present in plant. It also very valuable to designates about molecular structure and type of chemical bonding which are responsible for the reduction of silver ion.

**Fig 3:**
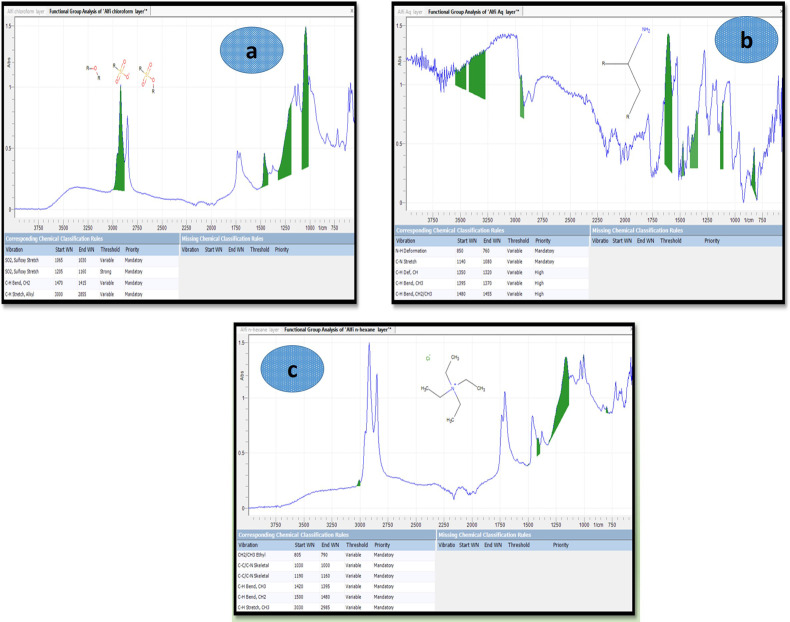
FTIR spectra of silver nanoparticles (AgNPs) synthesized using *Asplenium dalhousiae* extracts. a) AgNPs synthesized from aqueous extract, b) AgNPs synthesized from chloroform extract, and c) AgNPs synthesized from n-hexane extract.

FT-IR study on plant extracts and AgNP samples indicate the presence of different functional groups in plant. The strong broad band around 3,400 cm^−1^ indicates the presence of alcoholic, phenolic, and carboxylic groups, predominantly O-H stretching of hydroxyl groups, N-H stretching vibrations of amines and amides, and water molecules [27]. The C-N stretching vibration of aromatic amines is seen at 1,145 cm^−1^, whereas the primary and secondary amines and amides are showed by bands between 900–600 cm^−1^. Ketones, aldehydes, quinines, and esters are suggested by peaks between 1,700 and 1,600 cm^−1^ (C = O vibration). Additionally, C-H stretching of alkanes is observed at 2,920 cm^−1^, C = C vibration of aromatic structures at 1,630 cm^−1^, and C-O stretching of phenolic groups at 1,245 cm^−1^ [39]. A broad band at 1,040 cm^−1^ indicates aromatic ethers and polysaccharides (C-O-C stretch). The FT-IR results shown in (Fig 3) confirm that the surface of AgNPs is covered by organic bioactive compounds consequent from the plant extracts *Asplenium dalhousiae* used in the study.

### XRD analysis

**Fig 4:**
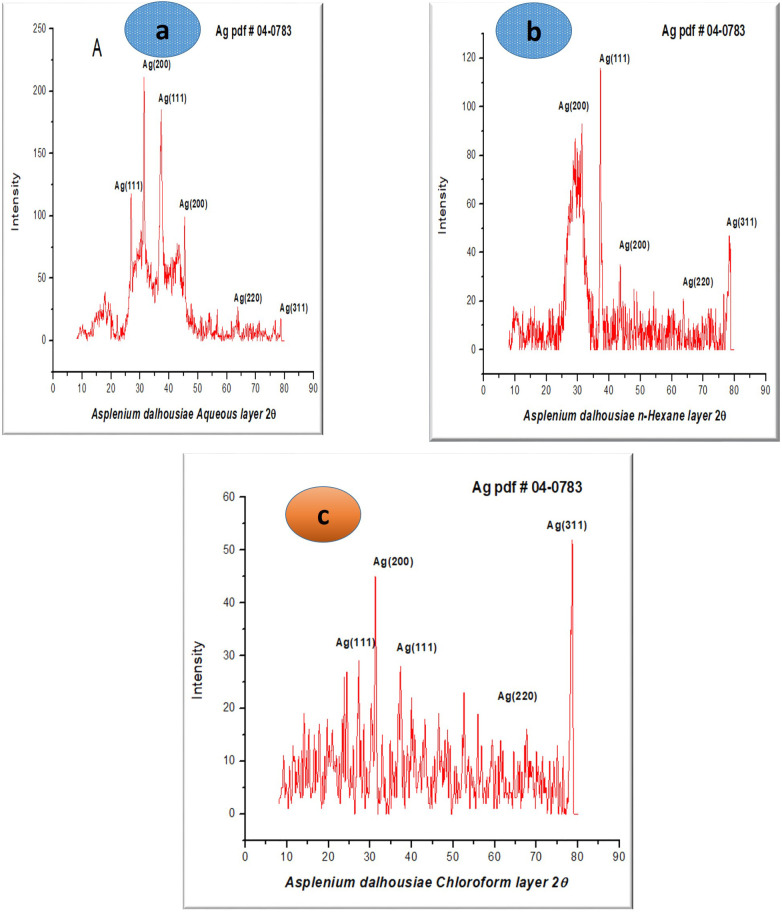
XRD patterns of silver nanoparticles (AgNPs) synthesized using *Asplenium dalhousiae* plant extracts: a) aqueous extract, b) n-hexane extract, c) chloroform extract.

The X-ray diffraction (XRD) analysis of the synthesized silver nanoparticles (AgNPs) from *Asplenium dalhousiae* confirmed their crystalline nature, with sharp peaks observed at 2θ values of 37.93°, 45.5°, 64.40°, and 77.08° (Fig 4a–4c). These peaks correspond to the (111), (200), (220), and (311) crystallographic planes of face-centered cubic (fcc) silver crystals, indicating a well-defined structure. Show in (Fig 4). The particle size was calculated to be approximately 20 nm using the Debye-Scherrer formula, which takes into account factors such as the X-ray wavelength, peak width, and Bragg angle. Notably, no impurities were identified in the sample, confirming the purity of the synthesized nanoparticles [41]. The data aligns with previous studies on silver nanoparticles, where similar 2θ values are associated with fcc silver structures. The sharpness of the peaks further supports the high crystallinity of the nanoparticles. The particle size of 20 nm falls within the typical range for biologically synthesized AgNPs, making them suitable for various applications such as antimicrobial agents or catalysts [36]. the study confirmed that the silver nanoparticles were well-defined and had a consistent diffraction profile across all samples.

### Scanning electron microscope (SEM)

**Fig 5:**
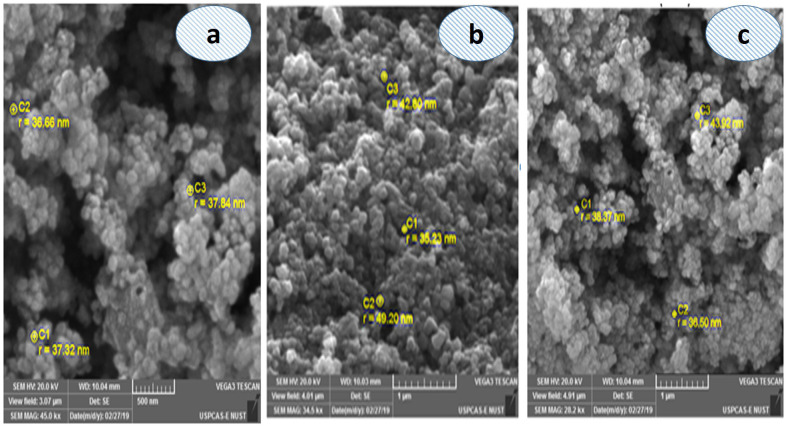
SEM images of silver nanoparticles (AgNPs) synthesized using *Asplenium dalhousiae* plant extracts: a) aqueous extract, b) n-hexane extract, c) chloroform extract.

The Scanning Electron Microscope (SEM) study of the synthesized silver nanoparticles consequent from plant extracts provided important insights into their morphology and structure. This high-resolution imaging technique allowed for the detailed examination of the nanoparticle surfaces, revealing their size, shape, and distribution [40–42]. The AgNPs synthesized using the aqueous extract (Fig 5a), *n*-hexane extract (Fig 5b), and chloroform extract (Fig 5c) exhibited varying morphological characteristics as observed in the SEM images. Among these, the nanoparticles derived from the *n*-hexane extract (Fig 5b) showed with more uniform and well-dispersed spherical particles. The particle sizes ranged from 1–100 nm, with most particles appearing below 100 nm in diameter, predominantly spherical in shape across all extracts (Fig 5).

### Energy dispersive x-ray (EDX)

**Fig 6:**
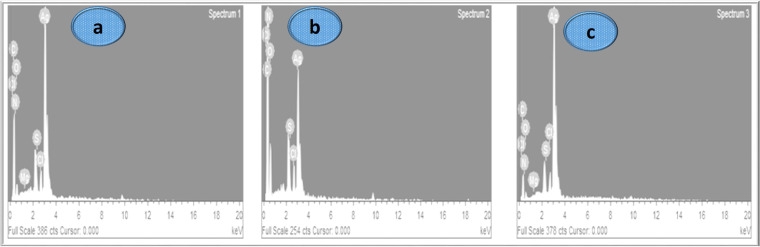
EDX spectra of silver nanoparticles (AgNPs) synthesized using *Asplenium dalhousiae* plant extracts: a) aqueous extract, b) chloroform extract, c) n-hexane extract.

The EDX spectrum showed a strong peak corresponding to silver metal, confirming the presence of silver nanoparticles in the samples. The spectrum provided valuable information about the elemental composition of the nanoparticles, (Fig 6) which is vital for understanding their properties and potential applications [43,44,46,47]. EDX indicated the existence of silver (94%) and oxides, (6%) confirming the purity of nanoparticles, showed in (Fig 6a–6c).

### Antibacterial activity of AgNPs

**Fig 7:**
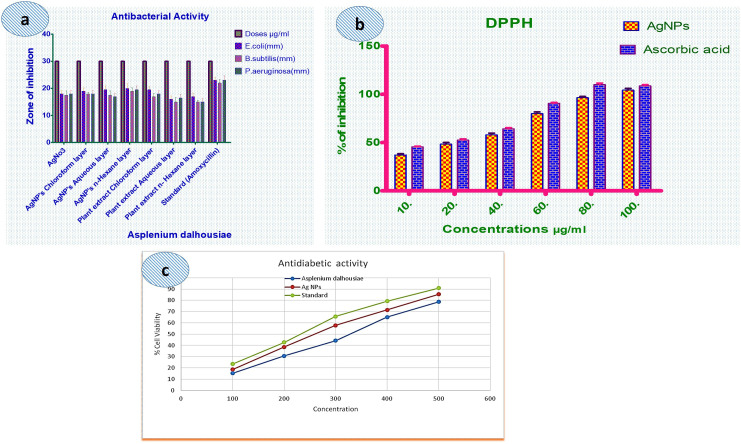
Graphical representation of DPPH, anti-bacterial, and anti-diabetic activity.

The antimicrobial activity of *Asplenium dalhousiae* different crude extract and synthesized AgNPs exhibited highest activity at the concentration of 30 μg/ml against three bacterial strains *Escherichia coli*, *Bacillus subtilis*, and *Pseudomonas aeruginosa*. (S1 Table). The results, showed as the mean zone of inhibition (ZOI, mm) and standard deviation (SD), show distinctive differences in activity between the tested substances. Silver nitrate (AgNO_3_) exhibited moderate antimicrobial activity, (Fig 7) with inhibition zones ranging from 17.5 mm to 18.0 mm across the bacterial strains. (S1 Table). These results suggest that AgNO_3_ has some antimicrobial potential, though its efficacy is not as high as some of the other treatments (Table 1).

**Table 1:** Antibacterial activity of silver nanoparticles and plant extracts against selected bacterial.

Group Format	Dose (µg/mL)	*E. coli* (mm)	*B. subtilis* (mm)	*P. aeruginosa* (mm)
AgNO₃	30	18.0 ± 1.2	17.5 ± 1.6	18.0 ± 1.2
AgNP of CHCl₃ extract	30	19.0 ± 1.2	18.0 ± 0.6	18.0 ± 1.4
AgNP Aqueous extract	30	19.5 ± 1.4	17.5 ± 1.6	17.0 ± 1.1
AgNP *n*-Hexane layer	30	20.0 ± 1.8	19.0 ± 1.2	19.5 ± 1.4
Plant extract of CHCl₃ extract	30	19.5 ± 1.0	17.0 ± 1.2	18.0 ± 1.2
Plant extract of Aqueous layer	30	16.0 ± 1.2	15.0 ± 1.6	16.5 ± 1.2
Plant extract of *n*-Hexane layer	30	17.0 ± 1.2	15.0 ± 0.6	15.0 ± 1.2
Standard (Amoxycillin)	30	23.0 ± 1.2	22.0 ± 1.2	23.0 ± 1.6

The was evaluated at a concentration of 30 µg/mL.

The mean zone of inhibition (ZOI, mm) ± standard deviation (SD) against *Escherichia coli*, *Bacillus subtilis*, and *Pseudomonas aeruginosa*.

Silver nanoparticles derived from chloroform (CHCl_3_), aqueous, and *n-*hexane extracts all demonstrated enhanced antimicrobial activity compared to AgNO_3_. Show in (Fig 7a) Among these, AgNPs from the *n-*hexane exhibited the maximum zones of inhibition, with values ranging from 19.0 mm to 20.0 mm across the three bacterial strains. AgNPs from the CHCl_3_ extract showed inhibition zones of 18.0 mm to 19.0 mm, however AgNPs from the aqueous extract had slightly lower activity, with inhibition zones ranging from 17.0 mm to 19.5 mm. Plant extracts also showed variable degrees of antimicrobial activity. The CHCl_3_ extract showed the highest activity with inhibition zones ranging from 17.0 mm to 19.5 mm. The aqueous extract and *n-*hexane extract extracts demonstrated less activity, with inhibition zones ranging from 15.0 mm to 17.0 mm, representing a lower efficiency in comparison to both silver nanoparticles and the standard antibiotic. (Table 2). The silver nanoparticles (AgNPs) showed anti-bacterial efficiency against both Gram-positive and Gram-negative bacteria [38,48,49]. silver nanoparticles, particularly those derived from the *n-*hexane layer, showed the most potent antimicrobial activity, especially against *E. coli* [42–44]. Plant extracts, while effective, exhibited relatively weaker activity compared to the silver nanoparticle formulations. Amoxicillin, as the standard antibiotic, demonstrated the highest inhibition ones, underscoring its broad-spectrum antimicrobial efficacy (Fig 7a).

**Table 2:** DPPH Radical scavenging capacity of ascorbic acid and (AgNPs).

Concentrations	Ascorbic Acid (Mean ± SD)	AgNPs Mean ± SD
10	45.69 ± 0.6	37.19 ± 1.2
20	52.54 ± 1.4	48.45 ± 1.6
40	64.27 ± 1.2	58.06 ± 1.8
60	90.77 ± 0.8	85.12 ± 1.6
80	110.06 ± 1.6	96.56 ± 1.2
100	108.77 ± 1.2	104.38 ± 1.8

### 2,2-Diphenyl-1-picrylhydrazyl (DPPH) radical assay

The study demonstrates the significant antioxidant activity of *Asplenium dalhousiae* extracts, with the CHCl₃ crude extract exhibiting the highest DPPH radical scavenging activity due to its efficiency in extracting bioactive compounds. The variation in IC₅₀ values among solvent extracts highlights the critical role of solvent polarity in optimizing the extraction of antioxidant compounds (S2 Table). In contrast, the aqueous extract displayed lower activity, Additionally, silver nanoparticles (AgNPs) derived from the extracts exhibited notable biological activity, with an IC₅₀ value of 23.64 μg/ml, slightly higher than ascorbic acid (17.78 μg/ml), (Table 3) yet reflecting strong potency (Fig 7b). AgNPs demonstrated a dose-dependent increase in cell viability, reaching 96.56% at 80 μg/ml and exceeding 100% viability at higher concentrations, suggesting their biocompatibility and therapeutic potential. (Table 2). The comparative DPPH scavenging activity of AgNPs alongside crude extracts is essential for a comprehensive assessment of their antioxidant properties (Fig 7b). These findings underscore the therapeutic promise of *Asplenium dalhousiae*, the importance of solvent selection for bioactive compound extraction, and the enhanced biological potential of AgNPs as an antioxidant agent for therapeutic applications.

**Table 3:** DPPH radical scavenging capacities of ascorbic acid and AgNPs.

S. No	Concentration (μg/ml)	% Cell Viability	
		AgNPs	Ascorbic Acid
1	10	37.19 ± 1.2	45.69 ± 1.2
2	20	48.45 ± 1.6	52.54 ± 1.6
3	40	58.06 ± 1.8	64.27 ± 1.8
4	60	85.12 ± 1.6	90.77 ± 1.6
5	80	96.56 ± 1.2	110.06 ± 1.2
6	100	104.38 ± 1.8	118.77 ± 1.8
IC_50_ (μg/ml)		23.64	17.78

### Inhibition of *in-vitro* α-amylase enzyme assay

The hypoglycemic effects of *Asplenium dalhousiae* leaf extract and silver nanoparticles (AgNPs), (S3 Table) demonstrating a concentration dependent inhibition of α-amylase activity. (Fig 7c) At a concentration of 100 µg/ml, the inhibition percentages were 15.25% for the leaf extract, 18.58% for AgNPs, and 23.45% for Acarbose. (Table 4). With an increased concentration of 500 µg/ml, the inhibition percentages significantly rose to 78.65% for the leaf extract, 85.04% for AgNPs, and 90.84% for Acarbose [50]. The half-maximal inhibitory concentration (IC_50_) values for the α-amylase inhibition activity were calculated as 320.26 μg/ml for the leaf extract, 274.04 μg/ml for AgNPs, and 239.40 μg/ml for Acarbose. (Fig 7c) Notably, AgNPs exhibited a substantial *in vitro* antidiabetic effect against α-amylase activity, showing efficacy that was closer to the standard drug Acarbose than the crude leaf extract.

**Table 4:** Inhibition of *in-vitro* α-amylase enzyme assay.

Concentration (μg/ml)	% Cell Viability		
	*Asplenium dalhousiae*	Ag NPs	Standard
100	15.25 ± 1.01	18.58 ± 1.30	23.45 ± 1.25
200	30.55 ± 1.50	38.5 ± 2.08	42.61 ± 2.45
300	44.16 ± 2.40	57.7 ± 3.09	65.74 ± 3.06
400	65.05 ± 3.01	71.44 ± 3.80	79.31 ± 3.70
500	78.65 ± 4.02	85.4 ± 4.09	90.84 ± 4.56
IC_50_ (μg/ml)	320.26	274.04	239.40

### Anticancer activity

The AgNPs were tested for their anticancer activity against HCT116 and A2780 cell lines with the lowest IC_50_ concentration at 5, 10 and 30 μg/ml for various exposure times of 24, 48 and 72 h. Generally, green synthesized silver nanoparticles manifested the significant anticancer activity with toxic manner depending on the agents responsible for reducing and capping the silver nitrate into silver [38,51].

**Fig 8:**
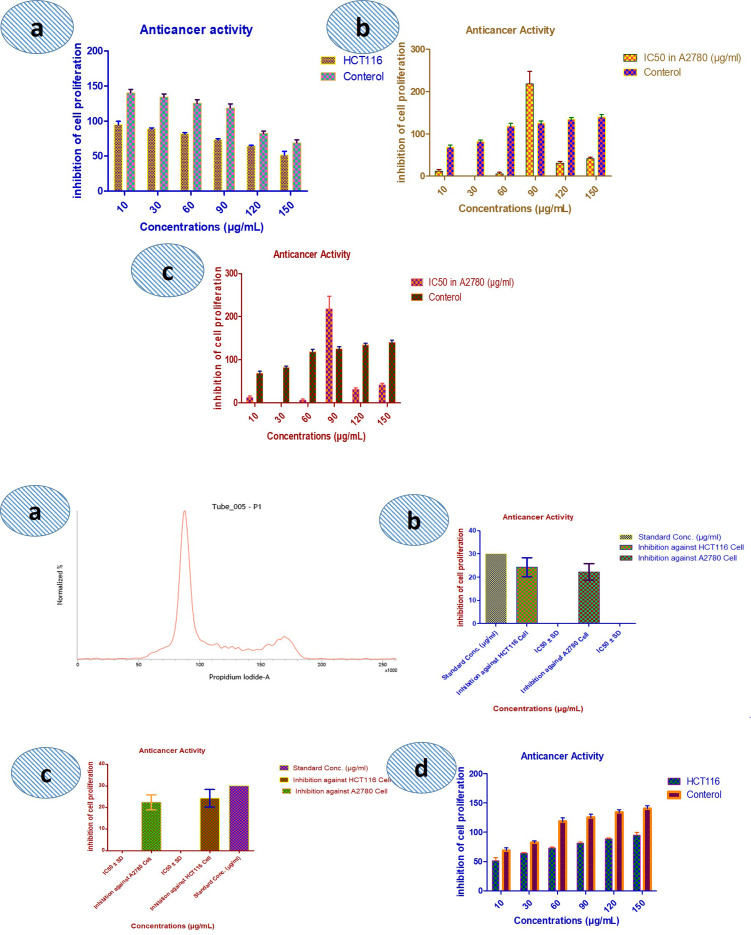
Graphical representation of the anticancer activity.

The *Asplenium dalhousiae* leaf extracts and silver nanoparticles (AgNPs) demonstrated significant inhibition of cell viability and proliferation in both A2780 and HCT116 cancer cell lines. The experimental results, analyzed using one-way ANOVA in the Origin program (version 8.5) and expressed as the mean ± standard deviation from three independent experiments, are presented in Table 5. At a concentration of 30 µg/ml, the inhibition against HCT116 cells was observed as follows: (Fig 8) the chloroform (CF) fraction showed 25.5% cell viability with an IC_50_ value of 50 ± 5 µg/ml, the hexane (HF) fraction showed 27.7% cell viability with an IC_50_ value of 45 ± 3 µg/ml, and the aqueous (AF) fraction showed 19.8% cell viability with an IC_50_ value of 40 ± 6 µg/ml. Similarly, in A2780 cells, the respective cell viability percentages were 23.2% (CF), 25.3% (HF), and 18.5% (AF), with corresponding IC_50_ values of 45 ± 4 µg/ml, 42 ± 4 µg/ml, and 38 ± 2 µg/ml (Fig 8). These results suggest that all samples inhibited cell proliferation in both cell lines, with the AF fraction showing the lowest cell viability and the most potent inhibitory effect in both HCT116 and A2780 cells, as indicated by the lower IC_50_ values (Table 5). Compared to previously reported data, the IC_50_ values of our samples are competitive [52,53]. For instance, *Asplenium* extracts in earlier studies have reported IC_50_ values above 50 µg/ml for the inhibition of cancer cell lines [40], while AgNPs typically show IC_50_ values around 40–60 µg/ml in similar cell lines [41]. Our findings, particularly with the AF fraction (IC_50_ of 38 ± 2 µg/ml in A2780 cells), demonstrate a more potent anticancer effect, highlighting the promising potential of *Asplenium dalhousiae* and AgNPs in cancer therapy. These results align with the findings of [42], who also demonstrated potent anticancer activity in plant based nanoparticle formulations with IC_50_ values in the similar range.

**Table 5:** The anticancer activity of different samples against HCT116 and A2780 cancer cell line.

Sample I.D. AgNPs	Standard Conc. (µg/ml)	Inhibition against HCT116		Inhibition against A2780	
		Cell Viability (%)	IC_50 _± SD	Cell Viability (%)	IC_50 _± SD
CF	30	25.5 ± 1.2	50 ± 5	23.2	45 ± 4
HF	30	27.7 ± 0.6	45 ± 3	25.3	42 ± 4
AF	30	19.8 ± 1.4	40 ± 6	18.5	38 ± 2

The combination of *Asplenium dalhousiae* leaf methanolic extracts and synthesized silver nanoparticles (AgNPs: aqueous, *n-*hexane, and CHCl_3_ fractions) exhibits varied apoptotic activity against ovarian and colorectal cancer cells. Among the tested samples, the aqueous AgNPs showed the highest potency with IC_50_ values of 21.11 µg/ml standard error of the mean 0.93 for HCT116 cells and 15.76 µg/ml (7.75) for A2780 cells. In compare, the CHCl_3_ AgNPs exhibited the lowest activity, with IC_50_ values of 605.2 µg/ml (258.3) for HCT116 cells and 247.66 µg/ml (188.64) for A2780 cells. The *n-*hexane fraction showed medium efficiency, with IC_50_ values of 45.98 µg/ml (29.56) and 9.11 µg/ml (2.55) for HCT116 and A2780 cells, respectively. (Table 6, S3 Fig). This values compared to previously reported studies, the aqueous and *n-*hexane fractions demonstrated superior cytotoxic activity, with IC_50_ values significantly lower than the 30 50 µg/ml range reported for similar nanoparticle formulations against ovarian cancer cells [43,44]. These findings highlight the enhanced efficacy of combining *Asplenium dalhousiae* extracts with AgNPs, likely due to bioactive compounds in the extracts synergizing with the nanoparticles to increase apoptotic activity. The results suggest a promising strategy for developing potent anticancer treatments.

**Table 6:** *Asplenium dalhousiae* AgNPs inhibition against HCT116 and A2780.

I.D No.	IC_50_ in HCT116 (µg/ml)	Sem	Replicate	IC_50_ in A2780 (µg/ml)	Sem	Replicate
AgNP’s aqueous	21.11	0.93	**2**	15.76	7.75	**2**
AgNP’s, *n-h*exane,	45.98	29.56	**2**	9.11	2.55	**2**
AgNP’s CHCl_3_	605.2	258.3	**2**	247.66	188.64	**2**
Plant methanolic Extract	603.25	45.35	2	34.97	27.38	2
**Result**	Active					

## Discussion

Green synthesis methods, which rely on natural materials, significantly reduce the risk of producing toxic byproducts that may adversely affect human health and the environment [32,33]. This approach is favored for its unique advantages, including non-toxic nature, environmental friendliness, cost-effectiveness, and suitability for biomedical applications, along with unique optical, electrical, catalytic, and magnetic properties [53]. Silver nanoparticles, in particular, have found extensive use in food industries, agriculture, biomedical settings, and drug delivery [54]. This study presents the green synthesis of silver nanoparticles (AgNPs) using *Asplenium dalhousiae* leaf extract, which acts as both a reducing and stabilizing agent in the reaction with silver nitrate salt. This method is noted for its eco-friendly and non-toxic nature, diverging from more traditional chemical synthesis methods that often involve hazardous substances. The synthesized nanoparticles were confirmed to be of pure silver through UV-VIS observed peak at 423 nm (aqueous extract), (Fig 2a) 420 nm (chloroform extract), (Fig 2b) and 425 nm (*n*-hexane extract), (Fig 2c). Many studies have been revealed the use of different plant extracts, such as *A. esculentus* flowers, *Euphorbia wallichii*, were used to synthesize silver nanoparticles efficiently [34,35]. Overall, the green synthesis of silver nanoparticles from *Asplenium dalhousiae* extract showcases a promising method for producing nanoparticles at 423 nm (Fig 2) and EDX analyses, (Fig 5) with sizes ranging from 1 to 100 nm as shown by SEM analysis. (Fig 6). This size range is particularly relevant for biomedical applications [39,40], where smaller particles can exhibit unique properties beneficial for therapy and diagnostics [48,49]. The use of distilled water, *n*-Hexane, and Chloroform as solvents in the synthesis process was explored, aiming to understand the effect of different solvents on the properties of the synthesized nanoparticles. The significance of this approach lies in its contribution to the field of green nanotechnology, providing a sustainable method to produce nanoparticles that are potentially less harmful to the environment and living organisms. *Asplenium dalhousiae* leaf extract not only plays a critical role in the synthesis of AgNPs but also exhibits substantial antioxidant properties. This is demonstrated by its ability to scavenge DPPH radicals, a common assay used to measure antioxidant capacity. This antioxidant activity is significant as oxidative stress is a known contributor to various diseases, including cancer, and antioxidants can help mitigate these effects. (Fig 7) Furthermore, the study investigates into the biological activities of the *Asplenium dalhousiae* leaf extract and synthesized AgNPs, particularly focusing on their enzymatic inhibition and anticancer properties. The α-amylase inhibitory effect, which exhibits a concentration-dependent manner, highlights the potential therapeutic applications in managing conditions such as diabetes, where slowing down the breakdown of starch can be beneficial. (Fig 7c). The most striking finding of this research is the enhanced cytotoxic effect against human ovarian cancer cells when combining *Asplenium dalhousiae* leaf extract with synthesized AgNPs [46,47]. This suggests that specific compounds within the leaf extract may synergistically interact with the silver nanoparticles, enhancing their ability to target and kill cancer cells. This observation is crucial as it opens new avenues for cancer treatment, where the goal is to develop therapies that are not only effective but also selective, and minimizing harm to healthy cells. This study not only advances the field of green nanotechnology by demonstrating an eco-friendly synthesis of silver nanoparticles but also illustrates the multifaceted potential of *Asplenium dalhousiae* leaf extract in therapeutic applications, ranging from antioxidant and enzymatic inhibition to potent anticancer activities. The integration of plant extracts with nanotechnology, as shown in this study, could lead to the development of novel therapeutic agents that are both effective and environmentally sustainable. In comparison to traditional chemical synthesis methods that involve toxic chemicals [51], our eco-friendly approach using *Asplenium dalhousiae* leaf extract aligns with the findings of [52], who synthesized silver nanoparticles using plant extracts and demonstrated their reduced toxicity in vivo [42]. The α-amylase inhibitory effect, which exhibits a concentration dependent manner, highlights the potential therapeutic applications in managing conditions such as diabetes, our results, showing concentration dependent α-amylase inhibition, align with the findings of [50,51], who observed similar effects in [52]. This further supports the potential of plant-derived AgNPs for diabetes management. *Asplenium dalhousiae* leaf extract with synthesized AgNPs. The enhanced cytotoxicity observed against human ovarian cancer cells is consistent with previous studies by [52–54], who reported similar findings with AgNPs synthesized. Our results further emphasize the synergistic potential of plant extract mediated nanoparticle synthesis in cancer therapy. This suggests that specific compounds within the leaf extract may synergistically interact with the silver nanoparticles, enhancing their ability to target and kill cancer cells. This study not only advances the field of green nanotechnology by demonstrating an eco-friendly synthesis of silver nanoparticles but also illustrates the multifaceted potential of *Asplenium dalhousiae* leaf extract in therapeutic applications, ranging from antioxidant and enzymatic inhibition to potent anticancer activities. The integration of plant extracts with nanotechnology in this study mirrors the approach [38], where plant based silver nanoparticles exhibited significant therapeutic potential. This highlights the growing interest in harnessing the combined power of natural products and nanotechnology for medical applications. The integration of plant extracts with nanotechnology, as shown in this study, could lead to the development of novel therapeutic agents that are both effective and environmentally sustainable.

## Conclusion

This study highlights a green and eco-friendly method for synthesizing silver nanoparticles (AgNPs) using *Asplenium dalhousiae* leaf extract, which serves as both a reducing and stabilizing agent. The synthesized AgNPs were characterized using UV-VIS, EDX, and SEM analyses, confirming their purity and nanoscale size (1–100 nm). The findings demonstrate significant biomedical potential, including antioxidant, enzymatic inhibition, and anticancer activities. Notably, the synergistic interaction between the leaf extract and AgNPs exhibited enhanced cytotoxicity against human ovarian cancer cells. These results align with previous findings by [44,54], who reported the biomedical efficacy of plant-mediated AgNPs in cancer therapy. Future studies should focus on further elucidating the molecular mechanisms underlying the enhanced anticancer effects and expanding the application of this green synthesis approach to other therapeutic targets. This integration of nanotechnology and natural products offers a sustainable platform for developing novel therapeutic agents that are environmentally friendly and biocompatible.

## Supporting information

S1 Fig Graphical abstract. (TIF)

S2 Fig Asplenium dalhousiae. (TIF)

S3 Fig Graphical representation of the anticancer activity. (TIF)

S1 Table Antibacterial activity of silver nanoparticles and plant extracts against selected bacterial raw data file. (TIF)

S2 Table DPPH Radical scavenging capacity of ascorbic acid and (AgNPs). (TIF)

S3 Table Inhibition of in-vitro α-amylase enzyme assay raw data file. (TIF)
