# Supplementary figures and images for: Biosynthesis and characterization of silver nanoparticles from Asplenium dalhousiae and their potential biological properties
Source: PLoS One. 2025 Jun 30;20(6):e0325533. doi: 10.1371/journal.pone.0325533 (PMC12208411; doi:10.1371/journal.pone.0325533)

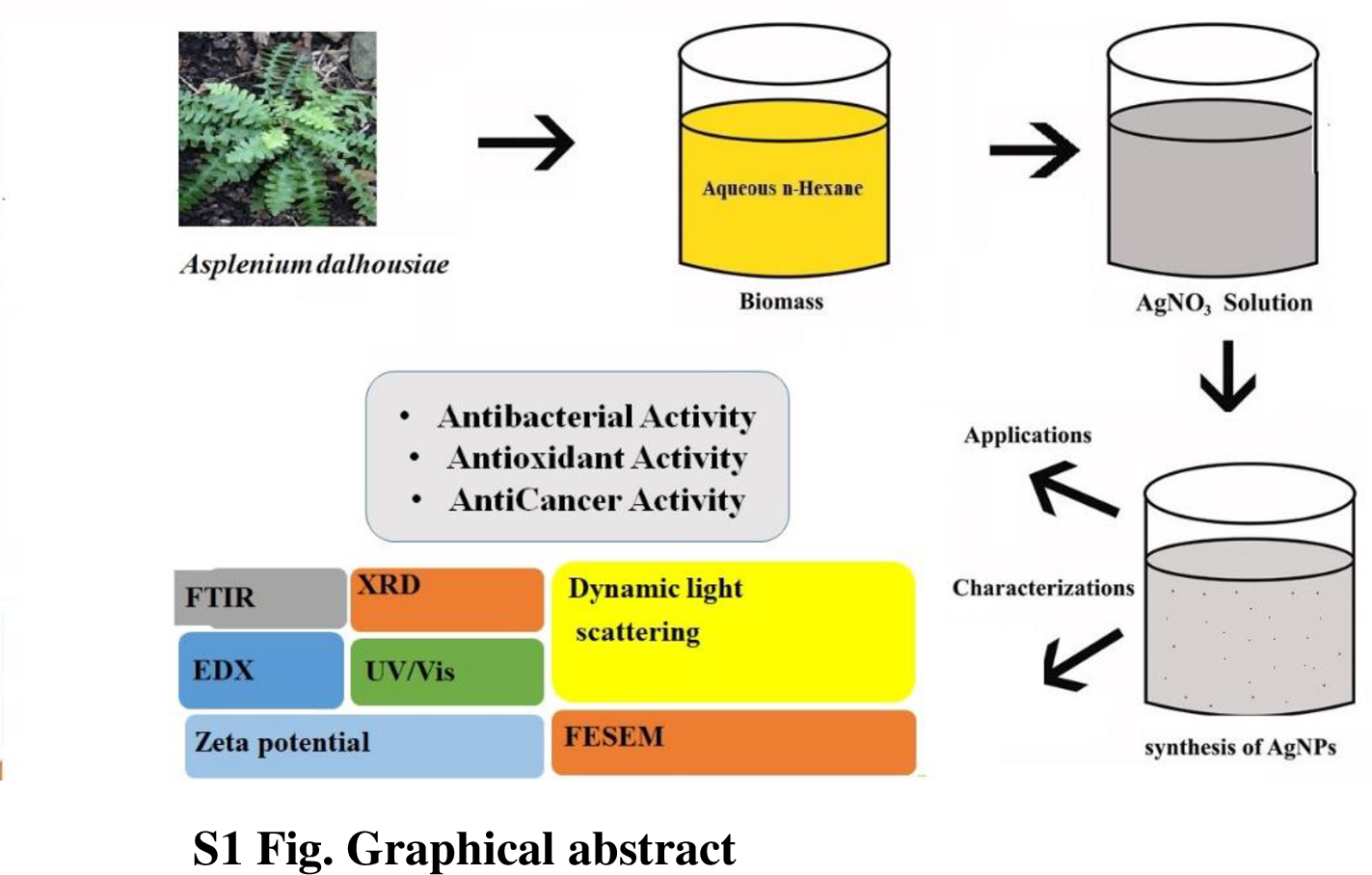

Supplement: S1 Fig — (TIF) [file pone.0325533.s001.tif]

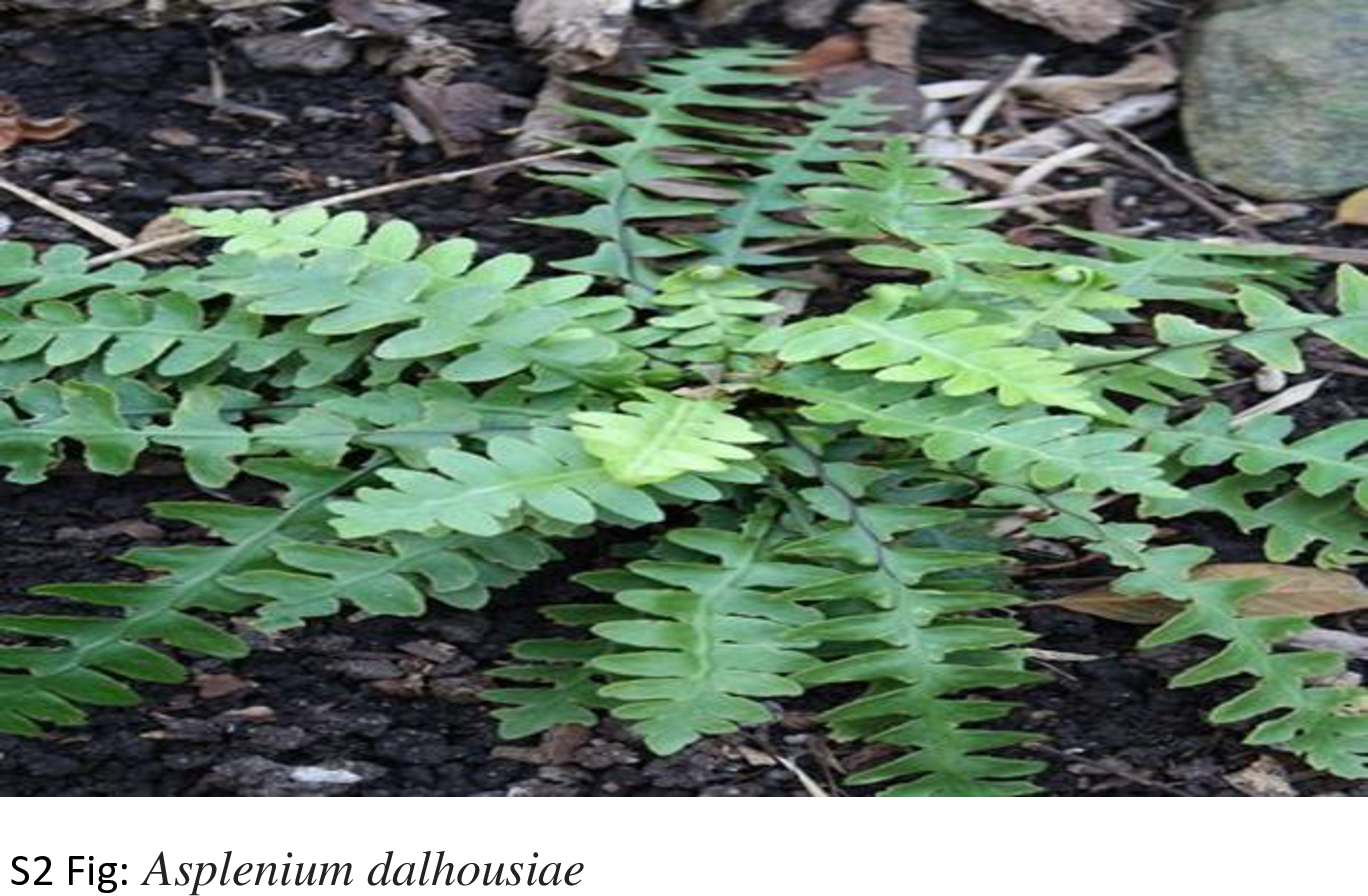

Supplement: S2 Fig — (TIF) [file pone.0325533.s002.tif]

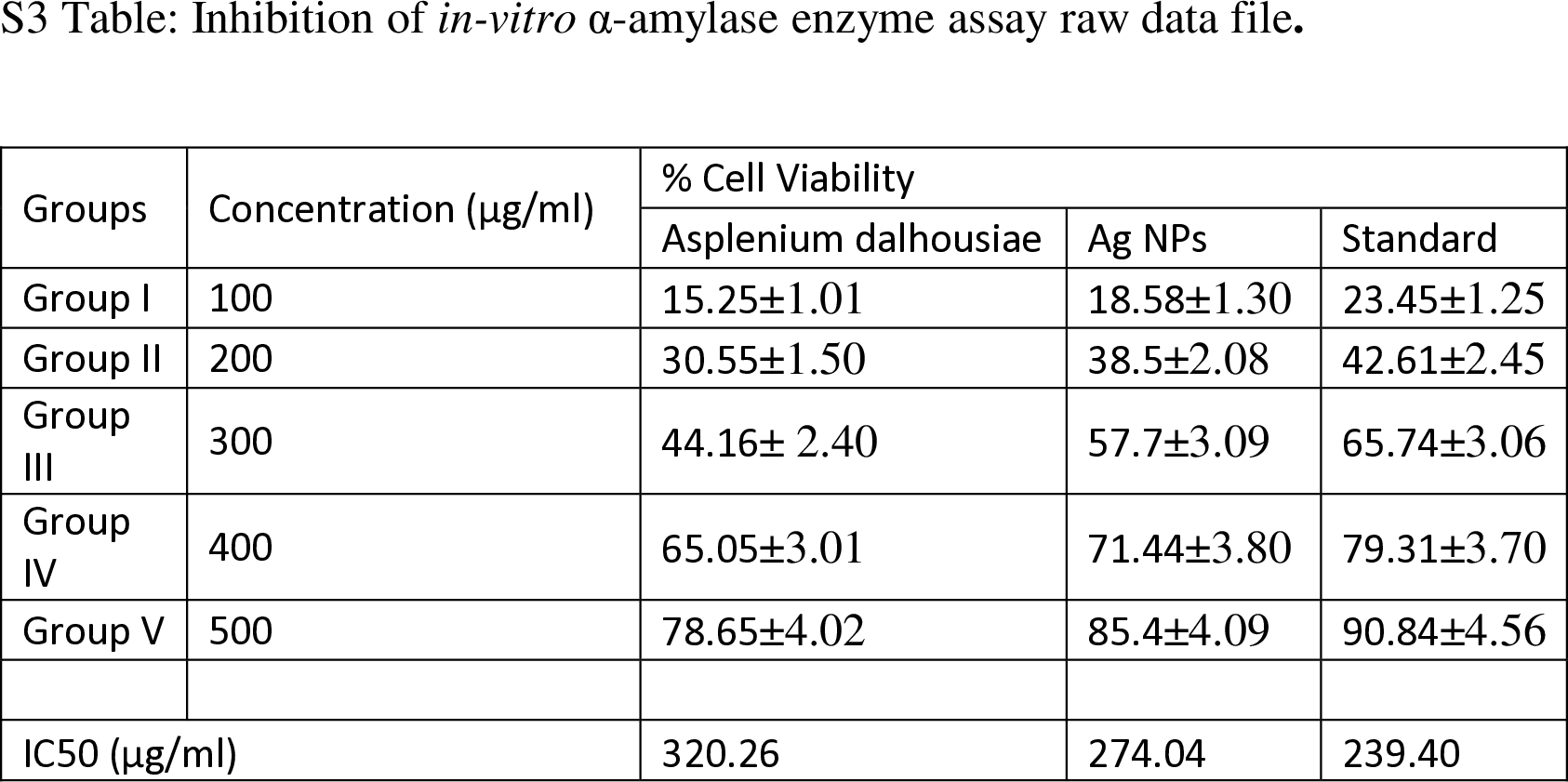

Supplement: S3 Fig — (TIF) [file pone.0325533.s003.tif]

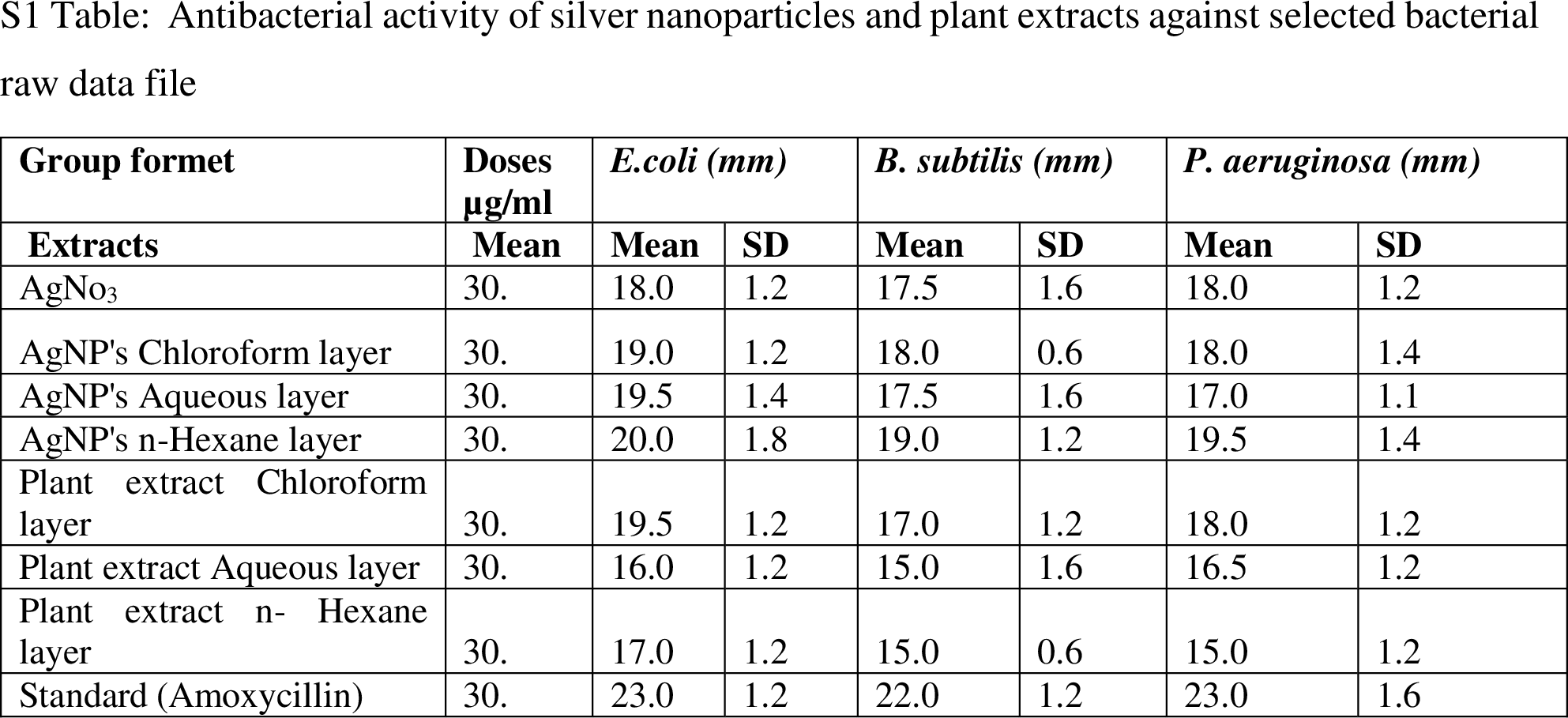

Supplement: S1 Table — (TIF) [file pone.0325533.s004.tif]

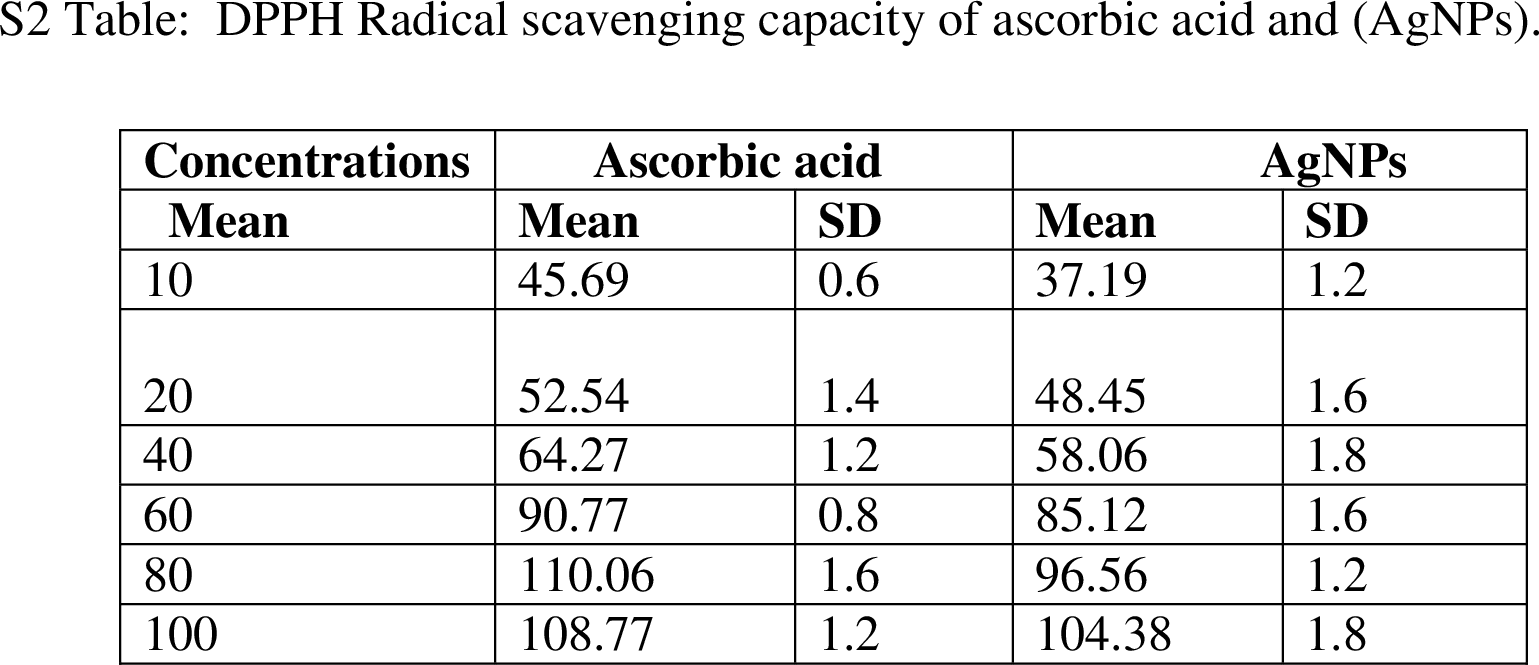

Supplement: S2 Table — (TIF) [file pone.0325533.s005.tif]
