# Supplementary material for: Biosynthesis and characterization of silver nanoparticles from Asplenium dalhousiae and their potential biological properties
Source: PLoS One. 2025 Jun 30;20(6):e0325533. doi: 10.1371/journal.pone.0325533 (PMC12208411; doi:10.1371/journal.pone.0325533)
Supplement: Attachment — Submitted filename: PONE-D-24-42400_reviewer.pdf [file pone.0325533.s007.pdf]

PLOS ONE

Biosynthesis and Characterization of Silver Nanoparticles from Asplenium dalhousiae and their Potential Biological Properties

--Manuscript Draft--

**Manuscript Number:**	PONE-D-24-42400
**Article Type:**	Research Article
**Full Title:**	Biosynthesis and Characterization of Silver Nanoparticles from Asplenium dalhousiae and their Potential Biological Properties
**Short Title:**	Biosynthesis and Characterization of Silver Nanoparticles from Asplenium dalhousiae"
**Corresponding Author:**	saima maher, Ph.D.<br>Sardar Bahadur Khan Women's University<br>Quetta, Balochistan PAKISTAN
**Keywords:**	Nanoparticles, Asplenium dalhousiae, Antibacterial, Antioxidant, α-amylase inhibitory,
**Abstract:**	This study investigates the synthesis of silver nanoparticles (AgNPs) using the medicinal plant Asplenium dalhousiae, exploring its bioactive chemical constituents for their potential as reducing agents. Aqueous, chloroform, and n-hexane extracts of the plant leaves were utilized in the nanoparticle synthesis process. The successful formation of AgNPs was confirmed through UV-visible spectroscopy, which exhibited absorption peaks at approximately 420 nm, 443 nm, and 439 nm. Fourier-transform infrared (FTIR) spectroscopy was employed to identify functional groups in the plant extracts that facilitate reduction. The morphological and structural characteristics of the synthesized nanoparticles were analyzed using scanning electron microscopy (SEM) and X-ray diffraction (XRD), revealing predominantly spherical AgNPs with a crystalline structure and an average diameter of 46.98 ± 12.45 nm. Stability assessments through zeta potential (ZP) and dynamic light scattering (DLS) measurements yielded values of -44.5 mV and 231.8 nm, respectively, indicating favorable physical stability. The antibacterial efficacy of the synthesized AgNPs was evaluated against Bacillus subtilis and Escherichia coli, demonstrating significant inhibition at a concentration of 30 µg/ml. The highest zones of inhibition recorded were 23.0 ± 1.2 mm for E. coli, 22.0 ± 1.2 mm for B. subtilis, and 23 ± 1.6 mm for Pseudomonas aeruginosa. Additionally, the α-amylase inhibitory activity of Asplenium dalhousiae leaf extract exhibited a concentration-dependent response, with a maximum inhibition percentage of 90.84% at a concentration of 500 µg/ml. Antioxidant potential was assessed using the DPPH method, confirming that the synthesized AgNPs possess significant antioxidant properties alongside their antibacterial activity. The antibacterial activity of Asplenium dalhousiae extract and synthesized AgNPs exhibited highest activity at the concentration of 30 µg/ml. The Escherichia coli (23.0 ± 1.2 mm) and P. aeroginosa (23 ± 1.6 mm) found the maximum zone of inhibition, compare to B. subtilis (22.0 ± 1.2 mm), The silver nanoparticles (AgNPs) from Asplenium dalhousiae confirmed significant α-amylase inhibition, with 85.04% inhibition at 500 µg/ml, as Acarbose (90.84%) and greater the leaf extract (78.65%). The aqueous and n-hexane silver nanoparticles (AgNPs) showed strong cytotoxic activity with low IC50 values, particularly in A2780 cells (15.76 µg/ml and 9.11 µg/ml, respectively), while the plant methanolic extract and CHCl3 AgNPs exhibited much higher IC50 values, indicating moderate to low activity. These findings highlight the promising role of integrating natural products with modern nanotechnology in developing novel therapeutic agents, particularly for cancer treatment. This innovative approach not only repurposes traditional medicinal plants but also enhances the functional capabilities of nanomaterials in medical applications, paving the way for improved treatment outcomes in patients with ovarian cancer and other diseases.
**Order of Authors:**	Shafia Parveen, M.Phil
	Shazia Iqbal, Ph.D.
	saima maher, Ph.D.
	Paul Wilson, Ph.D.
	Habib Nasir, PhD

Powered by Editorial Manager® and ProduXion Manager® from Aries Systems Corporation

	Shazia Shazia Nisar, PhD
	Muhammad Imran, PhD
	Musarat Riaz, PhD
	Rabail Urooj, PhD
	Mah Ganj Bakhshi Bakhshi, M.Phil
	Maria Essa, M.Phil
	Farah Mukhtar, PhD
	Attya Bhatti, PhD
	Hussnain A Janjua, PhD
	Amir Faisal, PhD
	Arsalan Saleem, M.Phil
Additional Information:	
Question	Response
**Financial Disclosure**<br><br>Enter a financial disclosure statement that describes the sources of funding for the work included in this submission. Review the submission guidelines for detailed requirements. View published research articles from *PLOS ONE* for specific examples.<br><br>This statement is required for submission and **will appear in the published article** if the submission is accepted. Please make sure it is accurate.<br><br>**Funded studies**<br>Enter a statement with the following details:<br>• Initials of the authors who received each award<br>• Grant numbers awarded to each author<br>• The full name of each funder<br>• URL of each funder website<br>• Did the sponsors or funders play any role in the study design, data collection and analysis, decision to publish, or preparation of the manuscript?<br><br>Did you receive funding for this work?	The author(s) received no specific funding for this work.
**Competing Interests**<br><br>Use the instructions below to enter a	The authors have declared that no competing interests exist.

competing interest statement for this submission. On behalf of all authors, disclose any competing interests that could be perceived to bias this work—acknowledging all financial support and any other relevant financial or non-financial competing interests.<br><br>This statement is required for submission and **will appear in the published article** if the submission is accepted. Please make sure it is accurate and that any funding sources listed in your Funding Information later in the submission form are also declared in your Financial Disclosure statement.<br><br>View published research articles from *PLOS ONE* for specific examples.<br><br>**NO authors have competing interests**<br><br>Enter: *The authors have declared that no competing interests exist.*<br><br>**Authors with competing interests**<br><br>Enter competing interest details beginning with this statement:<br><br>*I have read the journal's policy and the authors of this manuscript have the following competing interests: [insert competing interests here]*<br><br>* typeset	
**Ethics Statement**<br><br>Enter an ethics statement for this submission. This statement is required if the study involved:<br><br>• Human participants<br>• Human specimens or tissue<br>• Vertebrate animals or cephalopods<br>• Vertebrate embryos or tissues<br>• Field research	This all work perform in the research Laboratories

Powered by Editorial Manager® and ProduXion Manager® from Aries Systems Corporation

Write "N/A" if the submission does not require an ethics statement.

General guidance is provided below. Consult the submission guidelines for detailed instructions. **Make sure that all information entered here is included in the Methods section of the manuscript.**

Format for specific study types

Human Subject Research (involving human participants and/or tissue)

- Give the name of the institutional review board or ethics committee that approved the study
- Include the approval number and/or a statement indicating approval of this research
- Indicate the form of consent obtained (written/oral) or the reason that consent was not obtained (e.g. the data were analyzed anonymously)

Animal Research (involving vertebrate animals, embryos or tissues)

- Provide the name of the Institutional Animal Care and Use Committee (IACUC) or other relevant ethics board that reviewed the study protocol, and indicate whether they approved this research or granted a formal waiver of ethical approval
- Include an approval number if one was obtained
- If the study involved *non-human primates*, add *additional details* about animal welfare and steps taken to ameliorate suffering
- If anesthesia, euthanasia, or any kind of animal sacrifice is part of the study, include briefly which substances and/or methods were applied

Field Research

Include the following details if this study involves the collection of plant, animal, or other materials from a natural setting:

- Field permit number
- Name of the institution or relevant body that granted permission

**Data Availability**<br><br>Authors are required to make all data underlying the findings described fully available, without restriction, and from the time of publication. PLOS allows rare exceptions to address legal and ethical concerns. See the PLOS Data Policy and FAQ for detailed information.<br><br>A Data Availability Statement describing where the data can be found is required at submission. Your answers to this question constitute the Data Availability Statement and **will be published in the article**, if accepted.<br><br>**Important:** Stating 'data available on request from the author' is not sufficient. If your data are only available upon request, select 'No' for the first question and explain your exceptional situation in the text box.<br><br>Do the authors confirm that all data underlying the findings described in their manuscript are fully available without restriction?	Yes - all data are fully available without restriction
**Describe where the data may be found in full sentences. If you are copying our sample text, replace any instances of XXX with the appropriate details.**<br><br>• If the data are **held or will be held in a public repository**, include URLs, accession numbers or DOIs. If this information will only be available after acceptance, indicate this by ticking the box below. For example: *All XXX files are available from the XXX database (accession number(s) XXX, XXX.*).<br>• If the data are all contained **within the manuscript and/or Supporting Information files**, enter the following: *All relevant data are within the manuscript and its Supporting Information files.*<br>• If neither of these applies but you are able to provide **details of access elsewhere**, with or without limitations, please do so. For example:	All relevant data are within the manuscript and its Supporting Information files. If additional details or access are needed, please contact me directly.

Powered by Editorial Manager® and ProduXion Manager® from Aries Systems Corporation

Data cannot be shared publicly because of [XXX]. Data are available from the XXX Institutional Data Access / Ethics Committee (contact via XXX) for researchers who meet the criteria for access to confidential data.<br><br>*The data underlying the results presented in the study are available from (include the name of the third party and contact information or URL).*<br>• This text is appropriate if the data are owned by a third party and authors do not have permission to share the data.<br><br>* typeset	
Additional data availability information:	Tick here if your circumstances are not covered by the questions above and you need the journal's help to make your data available.; Tick here if the URLs/accession numbers/DOIs will be available only after acceptance of the manuscript for publication so that we can ensure their inclusion before publication.

Cover Letter

Click here to access/download;Manuscript;resubmit Final paper. PLos one.doc

Biosynthesis and Characterization of Silver Nanoparticles from *Asplenium dalhousiae* and their Potential Biological Properties

Shafia Parveen,[1] Shazia Iqbal, [2] Saima Maher,*[1] Paul Wilson,[3] Habib Nasir,[4] Shazia Nisar,[5] Muhammad Imran,[6] Musarat Riaz,[1]Rabail Urooj,[7] Mah Ganj Bakhshi,[1] Maria Essa,[1] Farah Mukhtar,[1] Attya Bhatti,[8] Hussnain A Janjua[8], Amir Faisal,[9] Arsalan Saleem,[10]

[1]Department of Chemistry, Sardar Bahadur Khan Women University Quetta.
[2]Department of Chemistry, Baluchistan University of Information Technology, Engineering and Management Sciences.
[3]Department of Chemistry, Warwick University, United Kingdom
[4] Department of Chemistry, School of Natural Sciences, National University of Sciences and Technology, H-12 Islamabad 44000, Pakistan.
[5] Department of Chemistry, University of Karachi, Pakistan,
[6]Department of Chemistry, Faculty of Science, King Khalid University, P.O. Box 9004, Abha 61413, Saudi Arabia.
[7]Department of Environmental Science, Sardar Bahadur Khan Women University Quetta
[8] Department of Industrial Biotechnology, Atta-ur-Rahman School of Applied Biosciences (ASAB), National University of Sciences and Technology, H-12 Islamabad 44000, Pakistan.
[9] Department of Biology, Lahore University of Management Sciences, D.H.A. Lahore Cantt. 54792, Pakistan.
[10]Department of Biological Sciences, COMSATS University, Islamabad 45550, Pakistan

Corresponding author: saimamaher@yahoo.com

Abstract

This study investigates the synthesis of silver nanoparticles (AgNPs) using the medicinal plant *Asplenium dalhousiae*, exploring its bioactive chemical constituents for their potential as reducing agents. Aqueous, chloroform, and *n*-hexane extracts of the plant leaves were utilized in the nanoparticle synthesis process. The successful formation of AgNPs was confirmed through UV-visible spectroscopy, which exhibited absorption peaks at approximately 420 nm, 443 nm, and 439 nm. Fourier-transform infrared (FTIR) spectroscopy was employed to identify functional groups in the plant extracts that facilitate reduction. The morphological and structural characteristics of the synthesized nanoparticles were analyzed using scanning electron microscopy (SEM) and X-ray diffraction (XRD), revealing predominantly spherical AgNPs with a crystalline structure and an average diameter of 46.98 ± 12.45 nm. Stability assessments through zeta potential (ZP) and dynamic light scattering (DLS) measurements yielded values of -44.5 mV and 231.8 nm, respectively, indicating favorable physical stability. The antibacterial efficacy of the synthesized AgNPs was evaluated against *Bacillus subtilis* and *Escherichia coli*, demonstrating significant inhibition at a concentration of 30 µg/ml. The highest zones of inhibition recorded were 23.0 ± 1.2 mm for *E. coli*, 22.0 ± 1.2 mm for *B. subtilis*, and 23 ± 1.6

mm for *Pseudomonas aeruginosa*. Additionally, the α-amylase inhibitory activity of *Asplenium dalhousiae* leaf extract exhibited a concentration-dependent response, with a maximum inhibition percentage of 90.84% at a concentration of 500 µg/ml. Antioxidant potential was assessed using the DPPH method, confirming that the synthesized AgNPs possess significant antioxidant properties alongside their antibacterial activity. The antibacterial activity of *Asplenium dalhousiae* extract and synthesized AgNPs exhibited highest activity at the concentration of 30 µg/ml. The *Escherichia coli* (23.0 ± 1.2 mm) and *P. aeroginosa* (23 ± 1.6 mm) found the maximum zone of inhibition, compare to *B. subtilis* (22.0 ± 1.2 mm), The silver nanoparticles (AgNPs) from *Asplenium dalhousiae* confirmed significant α-amylase inhibition, with 85.04% inhibition at 500 µg/ml, as Acarbose (90.84%) and greater the leaf extract (78.65%). The aqueous and n-hexane silver nanoparticles (AgNPs) showed strong cytotoxic activity with low IC50 values, particularly in A2780 cells (15.76 µg/ml and 9.11 µg/ml, respectively), while the plant methanolic extract and CHCl3 AgNPs exhibited much higher IC50 values, indicating moderate to low activity. These findings highlight the promising role of integrating natural products with modern nanotechnology in developing novel therapeutic agents, particularly for cancer treatment. This innovative approach not only repurposes traditional medicinal plants but also enhances the functional capabilities of nanomaterials in medical applications, paving the way for improved treatment outcomes in patients with ovarian cancer and other diseases.

Keywords: Nanoparticles, *Asplenium dalhousiae*, Antibacterial, Antioxidant, α-amylase inhibitory,

Introduction

Nanotechnology, a rapidly advancing interdisciplinary field including chemistry, biomedical science, material science, diagnostics, therapeutics, and medicine, comprises the production and application of nanoscale materials with various properties [1]. Its key significance is highlighted by its broad uses, predominantly catalyzing organic toxin reduction in environmental perceptions [2,3,4]. Furthermore, nanotechnologies have vital biological properties, such as anti-bacterial [5,6]. Anti-oxidant [7,8], and anti-cancer activities [9]. Many inorganic metallic elements, comprising iron, copper, zinc, gold, silver, magnesium, and titanium, have been utilized in the biological, physical, and chemical synthesis of nanomaterials for a several determinations. Chemical methods are involved the use of harmful chemicals and energy intensive procedures, raising concerns about their environmental effect and the formation of toxic by-products. In compare, green synthesis methods deal a more sustainable resolution by utilizing bio-based materials, such as microorganisms, plants, as eco-friendly sources for nanoparticle synthesis.

Many studies have confirmed that green synthesis not only reduces environmental harm but also effectively produces nanoparticles with desirable characteristics. Silver nanoparticles (SNPs) play a important role in advanced research due to their nano medicinal prospective. [10,11]. The green method in nanotechnology presents several advantages, encompassing cost-effectiveness, environmental affability, high yield, and precipitate synthesis [12]. In this method, the synthesis of silver nanoparticles (SNPs) apply extracts from various plant parts [13,14,15,16,17]. SNPs are known for their biocompatibility and are important in the medical and pharmaceutical domains [18]. such as antibacterial activities [19,20], antioxidant qualities [21,22], immunological advantages [23], and anticancer activity, have been identified by researchers. Silver nanoparticles have been proposed recently for the development of novel anticancer drugs due to their broad spectrum of biological activity [[9], [10], [11]]. Nanoparticles are far more dangerous to cancerous cells than bulk materials. The potential use of nanoparticles as therapeutic agents for cancer cell detection and management are rapidly growing. Moreover, drugs can be designed to release in a controlled manner at a specific site as nanoparticles. AgNPs have garnered important attention for their potential anticancer properties, due to their capability to inhibit the production of malignant cells. This inhibition is supposed to stem from their interference with various signaling pathways involved in cancer development and metastasis. Research indicates that AgNPs can exert cytotoxic effects on cancer cells, thereby hindering tumor growth and progression. In addition to their anticancer capabilities, medicinal plants have gained prominence for their therapeutic benefits, mostly in regions like Pakistan, where traditional medicine depend on greatly on plant-based remedies. The adverse effects associated with synthetic pharmaceuticals such as hypertension, hormonal imbalances, and weight gain have led to a resurgence in the use of natural products for medicinal purposes. A current study focused on the efficient exploration of *Asplenium dalhousiae*, a well-known medicinal plant. This research represents the first comprehensive investigation of this species, both within Pakistan and globally. highlighting its potential for synthesizing silver nanoparticles. The flowering period of *Asplenium dalhousiae* spans from July to December, thriving in moist environments typical of temperate and subtropical regions. The study developed extracts from *Asplenium dalhousiae* to synthesize AgNPs and subsequently evaluated their antibacterial and antioxidant properties, α-amylase enzyme assay, Anticancer activity. Despite the plant's recognized biological significance, its full potential remains underexplored. Previous reports have noted its antibacterial and antifungal activities, as well as anti-fertility effects observed in adult male rats. This pioneering research aims to expand knowledge regarding the biological applications of Asplenium dalhousiae, particularly in the synthesis of silver nanoparticles and their associated health benefits. This study contributes valuable insights into the sustainable production of

nanoparticles using natural resources, aligning with growing interests in green chemistry and eco-friendly practices in nanotechnology.

Material and Method

Sample collection, Identification and Analysis

The selected plant for this research, *Asplenium dalhousiae*, was collected from the Valley of Channat Bagh site in Azad Kashmir. Taxonomic identification was carried out by Dr. Amir of NARC Islamabad. The collected plant material was washed with distill water and then shade-drying, followed by uniform grinding the powder material was kept in refrigerator for further procedure. The subsequent scientific inquiries transpired within the well-equipped laboratories of the School of Natural Sciences (SNS), NUST. This academic institution played a pivotal role in facilitating various facets of the research. Characterization procedures took place in the SEME unit of the Energy department at SNS, while antibacterial assessments were carried out at IESE NUST and ASAB NUST.

Preparation of plant extracts *(Asplenium dalhousiae)*

The 500g of powdered dried plant material soaked in 100% methanol for 15 days at normal temperature [30]. The procedure was followed by filtration with help of 125 mm pore size Whatman filter paper. The filtrate was placed in rotatory evaporator to evaporate at 45^{0}C. The gummy crude extract were dissolved and suspended in distilled water (140 ml) and successively portioned with different organic solvent such as n-Hexane (3×200ml), $CHCl_3$ (3×200ml) and a water layer (3×200ml).

Preparation of Ag-NPs

The AgNO3 solution was prepared using a accurate measurement of 100mg/100ml in deionized water. The solution was subjected in oil bath for 24 hrs. The prepared solution was centerifugated at 4000 rpm for 25min to collect the silver nanoparticles. The prepared silver nanoparticles were washed with distill water three times and vacuum dried for a night at 60 ^{0}C. The sample was re-suspended with distilled water for characterization.

Experimental and Characterization of Silver Nanoparticles

UV-Vis spectroscopy plays an important role for examining the reduction of silver ions to silver nanoparticles. The UV spectra of *Asplenium dalhousiae* plant extract were taken at different

concentrations the range was 250 mL to 650 mL in 10 mL $AgNO_3$ solution. UV-2800, BMS model was used in this research. The spectra of AgNps were recorded between 300 and 800 nm. The UV-Vis spectrum demonstrates the surface Plasmon resonance (SPR) absorption band of AgNps nanoparticles, showing a gradual decrease in absorbance with a shift in wavelength and changes in band width. This finding underscores the significance of the reduction of silver ions to silver nanoparticles and characterizing the silver optical properties of silver nanoparticles and structural features. **Fourier Transform Infrared (FTIR),** is used to recognize the functional group existent in plant. This technique, highlighting the role of bioactive plants chemicals in reduction and capping processes. The IR spectra were recorded with support of Perklin Elmer spectrum 100 models. **X-ray diffraction** is a very advantageous technique to determine the crystalline nature structure and characterization of synthesized nanoparticles through X-ray diffractogram. The instrumental model was used XRD; D8 Advance; Bruker, Billerica, MA, USA. **Scanning Electron Microscopy (SEM),** the morphology and size of synthesized silver nanoparticles (Ag-NPs) were examined using the JEOL-6490A-JSM (SEM). **Energy dispersion X-ray (EDX)** was employed to acquire elemental composition of synthesized silver nanoparticles.

Preparation of Asplenium dalhousiae Extract and Ag-NPs for Antibacterial Activity

The plant extract was prepared, including n-Hexane, chloroform and, water extract, along with $AgNO_3$ and Ag-NPs aqueous, chloroform, and *n*-Hexane layers, were weighed at 10mg/10ml and dissolved in sterile distilled water for Ag-NPs synthesis, 1mM silver nitrate solution was dissolve with plant extract to make a solution of 100 ml, and centrifuged at 4,000 rpm for 25min. The collected obtained pellets were used. The disc diffusion method was applied for antibacterial activity. [31]. Amoxicillin+Clavuanic acid was used as standard solution. The nutrient agar was prepared by suspending 28.0 grams in 1000 ml of distilled water and sterilizing it by autoclaving at 15 Ibs pressure (121°C) for 15 minutes. The agar was then mixed well and poured into prepared sterile petri plates. *Asplenium dalhousiae* leaves extracts and its synthesized silver nanoparticles were studied. The Gram-positive bacteria and Gram-negative bacteria strains were used in the biological assays. Nutrient agar was spread on sterilizes petri plates suspended in about 10ml of physiological saline in a Roux bottle and was incubated at 37ºC for 24 hours. The preparation of antibiotic sensitivity discs involves using Whatman No. 1 filter paper to create discs approximately 1mm in diameter. These discs are then sterilized by placing them in hot air. After sterilization, each disc is loaded with 30µl of the sample extract, silver nanoparticles (AgNPs), and a standard solution such as Amoxicillin+Clavuanic acid (30mg/ml distilled water). Subsequently, the loaded discs are refrigerated for 24 hours before being used for experiments.

Microorganisms

The antimicrobial assay of Ag nanoparticles was examined against The following bacteria were tested the Gram-negative *Escherichia coli* (ATCC 25922) and *Pseudomonas aeruginosa* (ATCC 27853), as well as the Gram-positive *Bacillus subtilis* (ATCC 13048). Nutrient agar was used and containing 50 ml of NA /PDA medium for bacteria. The potato dextrose agar was dried for 10 mints. The selected bacterial strains were inoculated in nutrient broth for the growth of microorganisms.

Evaluation of Anti-oxidant activity by DPPH assay

The antioxidant assay of plant three extracts a (*n*-hexane, chloroform and aqueous, extracts) and synthesized nanoparticles were evaluated by methanolic DPPH assay. This assay is constructed on the reduction of a DPPH in the presence of a reducing agent or antioxidant, the result of this reaction is the development of a stable non-radical DPPH-H molecule. The Ag-NPs solution was prepared in methanol at different concentrations the range were 10–100 µg/ml, and 200µl from all dilution of silver nanoparticle solutions were added to 0.8 µl of DPPH solution. The samples were incubated in the dark for 30 minutes at room temperature, and the absorbance of each solution was read at 517 nm. Ascorbic acid was used as a positive control, and the antioxidant capacity to scavenge the DPPH radical was calculated as a percentage using the formula:

DPPH scavenging capacity (%) = [(A0 – A1) / A0] × 100

Where A0 is the absorbance of the control

A1 is the absorbance of the sample [1].

In-vitro α-amylase enzyme assay

The inhibition of *in-vitro* α-amylase enzyme assay was performed by using *Asplenium dalhousiae* different extracts and silver nanoparticles (Ag-NPs) as well as standard (acarbose). The assay was constructed on the previously described method of (Bernfield), which measures the capability of the plant crude extracts to inhibit α-amylase in a dose-dependent manner [32] .The results showed that *Asplenium dalhousiae* extract and Ag-NPs showed concentration dependent inhibition. The plant extracts showing a higher α-amylase of inhibitory effect compared comparatively to AgNPs. The IC_{50} value` for the extract and synthesized Ag-NPs were estimated, and the results specified that the extract had a lower IC_{50} value.

Anticancer activity

Anticancer activity against HCT116 and A2780 cell lines indicates to the capability of a compound to inhibit the growth and propagation of cancer cells derived from these specific cell lines. The efficiency of the constituent in inhibiting cancer cell growth is then calculated by measuring parameters such as cell viability, proliferation, apoptosis induction, and cell cycle capture. Substance representative anticancer activities against these cell lines have potential therapeutic implications for treating colorectal cancer (HCT116) and ovarian cancer (A2780), respectively. This activity is typically evaluated through *in vitro* assays where cancer cells are exposed to the substance of interest, such as the tested AgNPs, at various concentrations and exposure times.

Result and Discussion

UV-Vis spectroscopy

The green synthesis of silver nanoparticles using the plant extract of *Asplenium dalhousiae* resulted in a color change from pale yellow to orange-brown, representing the formation of silver nanoparticles the UV-Vis results showing absorption peaks at 423 nm (aqueous extract), 420 nm (chloroform extract), and 425 nm (n-hexane extract). These absorption peaks support with previous reported data [33], of silver nanoparticles at 400 nm. Particularly, the broadening of the peak detected in spectra of the *n*-hexane as the higher concentration of silver nanoparticles it was compared with the other two extracts. Many studies have been revealed the use of different plant extracts, such as *A. esculentus* flowers, *Euphorbia wallichii*, were used to synthesize silver nanoparticles efficiently [34,35]. Overall, the green synthesis of silver nanoparticles from *Asplenium dalhousiae* extract showcases a promising method for producing nanoparticles.

Fourier transforms infrared spectroscopy

Fourier transformed infrared spectroscopy is used to identified the function group present in plant. It also very valuable to designates about molecular structure and type of chemical bonding which are responsible for the reduction of silver ion. FT-IR study on plant extracts and AgNP samples indicate the presence of different functional groups in plant. The strong broad band around 3,400 cm^{-1} indicates the presence of alcoholic, phenolic, and carboxylic groups, predominantly O-H stretching of hydroxyl groups, N-H stretching vibrations of amines and amides, and water molecules. The C-N stretching vibration of aromatic amines is seen at 1,145 cm^{-1}, whereas the primary and secondary amines and amides are showed by bands between 900–

600 cm^{-1}. Ketones, aldehydes, quinines, and esters are suggested by peaks between 1,700 and 1,600 cm^{-1} (C=O vibration). Additionally, C-H stretching of alkanes is observed at 2,920 cm^{-1}, C=C vibration of aromatic structures at 1,630 cm^{-1}, and C-O stretching of phenolic groups at 1,245 cm^{-1}. A broad band at 1,040 cm^{-1} indicates aromatic ethers and polysaccharides (C-O-C stretch). The FT-IR results confirm that the surface of AgNPs is covered by organic bioactive compounds consequent from the plant extracts *Asplenium dalhousiae* used in the study.

XRD analysis

The X-ray diffraction (XRD) analysis of the synthesized silver nanoparticles from *Asplenium dalhousiae* confirmed a crystalline nature, with the main phase being AgNPs and no evident impurities identified. The size of the Ag-NPs was determined to be 20nm using the Debye-Scherrer formula, which considers factors like X-ray wavelength, peak width, and Bragg angle. The XRD showed sharp peaks at 2θ values of 37.93°, 45.5°, 64.40°, and 77.08° corresponding to the crystallographic planes of face-centered cubic (fcc) silver crystals. [36]. the study confirmed that the silver nanoparticles were well-defined and had a consistent diffraction profile across all samples.

The Scanning Electron Microscope (SEM)

The Scanning Electron Microscope (SEM) study of the synthesized silver nanoparticles consequent from plant extracts provided important insights into their morphology and structure. This high-resolution imaging technique allowed for the detailed examination of the nanoparticle surfaces, revealing their size, shape, and distribution. The AgNPs synthesized in *n*-hexane showed better results compared to aqueous and chloroform extract. The particle size ranged from 1-100nm, with the SEM images displaying mostly spherical particles below 100 nm in size.

Energy Dispersive X-ray (EDX)

The EDX spectrum showed a strong peak corresponding to silver metal, confirming the presence of silver nanoparticles in the samples. The spectrum provided valuable information about the elemental composition of the nanoparticles, which is vital for understanding their properties and potential applications. EDX indicated the existence of silver (94%) and oxides, (6%) confirming the purity of nanoparticles.

Antibacterial Activity of ApNPs and Statistical Analysis

The silver nanoparticles (AgNPs) showed anti-bacterial efficiency against both Gram-positive and Gram-negative bacteria. The statistical analysis involved expressing the results as Mean ±

SD it is used to determine significant differences in each samples, a one-way analysis of variance (ANOVA) was conducted, followed by the Duncan's test for multiple comparisons, as outlined by Harvey and Paige (1998). [37]. The statistical analysis was carried out using Graph Pad Prism Software, version 5. A significance level of $p < 0.05$ was considered, indicating that results with a p-value below this threshold were deemed statistically significant. This approach provided a comprehensive evaluation of the antibacterial activity of Ag-NPs and their effectiveness against different bacterial strains.

The antibacterial activity of *Asplenium dalhousiae* extract and synthesized AgNPs exhibited highest activity at the concentration of 30 µg/ml. The *Escherichia coli* (23.0 ± 1.2 mm) and *P. aeroginosa* (23 ± 1.6 mm) found the maximum zone of inhibition, compare to *B. subtilis* (22.0 ± 1.2 mm), and the synthesized AgNPs from the plant extract also exhibited the antibacterial activity against both Gram-positive and negative bacteria, the antibacterial activity were performed on dose-dependent. In highest concentration of AgNPs demonstrated the significance inhibitory effect on the growth of bacterial organisms.

2,2-diphenyl-1-picrylhydrazyl (DPPH) radical Assay

DPPH is a highly stable and very well-known free radical. It is based on the reduction of accepting hydrogen or electron from donors. The DPPH reducing capability of the AgNPs was evaluated by detecting color change. The DPPH scavenging assay demonstrated effective inhibition activity of AgNPs when related with the standard (ascorbic acid). The ability of plant extracts to scavenge the 2, 2-diphenyl-1-picrylhydrazyl (DPPH) radical. The absorbance of the resultant solution was measured at a wavelength of 517 nm, the previously defined protocol was adopted with slightly modified [38]. The standard ascorbic acid was used as a positive control for comparison with the plant extract. The % of DPPH scavenging capability was measured using the following formula:

DPPH scavenging capacity (in percentage) = $[A^0 – A1/A^0] \times 100$

Where:

(A_0) represents the absorbance of the control (ascorbic acid).

(A_1) represents the absorbance of the sample (AgNPs)

The results of this study specify that *Asplenium dalhousiae* extracts possess prominent antioxidant activity, as confirmed by their capability to scavenge DPPH radicals. The difference in IC_{50} values of extracts highlights the importance of polarity bases solvent in extracting bioactive chemical compounds with antioxidant ability. The highest DPPH scavenging activity of the crude methanol extract suggests that this solvent is particularly effective in extracting antioxidant compounds from *Asplenium dalhousiae*. But the aqueous extract showed lower

activity. The observed differences in IC_{50} values among the solvent extracts could be attributed to variations in the types and concentrations of bioactive compounds present in each extract. Further investigation into the chemical composition of these extracts could provide insights into the specific antioxidant compounds responsible for their activity. These findings suggest that the antioxidant potential of *Asplenium dalhousiae* and emphasize the significance of solvent selection in optimizing the extraction of bioactive compounds for potential therapeutic applications.

Inhibition of in-vitro α-amylase enzyme assay

Alpha amylase inhibitors attach to the alpha bonds of polysaccharides like glycogen and starch, preventing their breakdown into glucose and maltose. These inhibitors play a vital role in several applications, and management human diseases. They work by reducing endogenous alpha amylase activity, thereby impeding the conversion of complex carbohydrates and disaccharides into absorbable monosaccharides. Clinical trials have shown that these inhibitors delay the digestion of complex carbohydrates and disaccharides, inhibiting α-glucosidases and impacting the absorption of glucose. Polyphenols found in dietary sources act as natural inhibitors of α-amylase and α-glucosidase, slowing down glucose absorption by interfering with these key enzymes involved in carbohydrate digestion

The α-amylase inhibitors found in *Asplenium dalhousiae* leaf extract and silver nanoparticles (AgNPs) exhibit concentration-dependent inhibition of α-amylase activity. At 100 µg/ml concentration, the inhibition percentages were 15.25% for the leaf extract, 18.58% for AgNPs, and 23.45% for Acarbose. At a higher concentration of 500 µg/ml, the inhibition percentages increased significantly to 78.65% for the leaf extract, 85.04% for AgNPs, and 90.84% for Acarbose. The half inhibition concentration (IC_{50}) values for the α-amylase inhibition activity of Asplenium dalhousiae leaf extract, AgNPs, and Acarbose were 320.26 µg/ml for the leaf extract, 274.04 µg/ml for AgNPs, and 239.40 µg/ml for Acarbose. Notably, AgNPs demonstrated a substantial *in vitro* antidiabetic effect against α-amylase activity, with results comparable to the standard drug Acarboseare and then the leaf extract

Anticancer Activity

The AgNPs were tested for their anticancer activity against HCT116 and A2780 cell lines with the lowest IC_{50} concentration at 5, 10 and 30 µg/ml for various exposure times of 24, 48 and 72 h. Generally, green synthesized silver nanoparticles manifested the significant anticancer activity with toxic manner depending on the agents responsible for reducing and capping the silver nitrate into silver [39, 40]. Propidium iodide (PI) is normally used along with Annexin V to evaluate cell viability of apoptosis and necrosis, on the bases of differences in plasma membrane

integrity and permeability. propidium iodide (PI) gives information to the researchers about identify different types of cell death either necrosis or apoptosis. It is depending on two components, annexin V, DNA-binding dye molecule PI, the annexin V, is a protein and binds with phospholipids and it is called phosphatidylserines, which generally occur in inner, cytoplasm fronting leaflet of a cell's membrane, but become "flipped" to the outer leaflet during the early stages of apoptosis. The DNA-binding dye molecule PI, which can only enter cells when their membranes are ruptured a characteristic of both necrosis and late apoptosis. [41]. The *Asplenium dalhousiae* leaf extracts and silver nanoparticles (AgNPs) established a significant inhibition of capability and proliferation in both A2780 and HCT116 cells. The experimental results, stated as the mean standard deviation of three independent experiments, were examined using one-way ANOVA in the Origin program version 8.5. The inhibition data against HCT116 and A2780 cells for different samples, including, and $CHCl_3$ extracts were presented in Table 3. Notably, The anticancer activity at a concentration of 30 µg/ml. For HCT116 cells, the cell viability percentages were 25.5% (CF), 27.7% (HF), and 19.8% (AF), all classified as active. In A2780 cells, the viability percentages were 23.2% (CF), 25.3% (HF), and 18.5% (AF), indicating active inhibition across all samples. all samples were classified as "Active" in terms of their inhibition against the cell lines, indicating a substantial effect on cell viability and proliferation.

The data suggests that the combination of *Asplenium dalhousiae* leaf methanolic extracts and synthesized silver nanoparticles (AgNPs of Aqueous, hexane and $CHCl_3$) exhibits potent apoptotic activity in human ovarian cancer cells. This combined treatment demonstrated a higher cytotoxic effect on ovarian cancer cells compared to the effect induced by AgNPs alone. The synergistic action of *Asplenium dalhousiae* leaf extracts and AgNPs presents a promising approach for enhancing the apoptotic activity in ovarian cancer cells, potentially offering a more effective treatment strategy. In the investigation of alternative treatments for human ovarian cancer, the application of *Asplenium dalhousiae* leaf extracts in conjunction with silver nanoparticles (AgNPs) has emerged as a notable area of study. The focus of this discussion is the observed potent apoptotic activity induced by this combined treatment in ovarian cancer cells, which underscores a significant advancement in the realm of cancer therapy. The study reveals that *Asplenium dalhousiae* leaf extracts are combined with AgNPs, the fabrication exhibits a

remarkable ability to induce programmed cell death, or apoptosis, in ovarian cancer cells. This is particularly noteworthy because apoptosis is a crucial mechanism for eliminating cancer cells in a controlled manner, thereby preventing their proliferation. The fact that this combined treatment outperforms the application of AgNPs alone in inducing cytotoxic effects offers a promising avenue for enhancing the efficacy of cancer treatments. However, the novel finding that the addition of *Asplenium dalhousiae* leaf extract to AgNPs significantly increases the cytotoxic effect against ovarian cancer cells suggests that compounds within the leaf extract may play a pivotal role in enhancing the nanoparticles' ability to target and kill cancer cells. The IC_{50} values for HCT116 cells were 21.11 µg/ml for aqueous AgNPs (Sem 0.93), 45.98 µg/ml for n-hexane AgNPs (Sem 29.56), and 605.2 µg/ml for CHCl3 AgNPs (Sem 258.3). In A2780 cells, the IC_{50} values were 15.76 µg/ml for aqueous AgNPs (Sem 7.75), 9.11 µg/ml for n-hexane AgNPs (Sem 2.55), and 247.66 µg/ml for CHCl3 AgNPs (Sem 188.64). The plant methanolic extract displayed IC_{50} values of 603.25 µg/ml (Sem 45.35) for HCT116 and 34.97 µg/ml (Sem 27.38) for A2780. Overall, the results indicate significant anticancer activity, particularly from the aqueous and n-hexane AgNPs. This synergistic effect, where the combined treatment is more effective than the sum of its parts, indicates that the *Asplenium dalhousiae* leaf extract could be modulating the biological response of the cancer cells to AgNPs, possibly by making them more susceptible to apoptosis. Such an interaction between a natural plant extract and nanoparticles opens up new paths for the development of cancer therapies that are both more efficient and potentially less harmful to normal cells, given the targeted nature of the treatment.

Conclusion

This study presents an innovative approach to the green synthesis of silver nanoparticles (AgNPs) using *Asplenium dalhousiae* leaf extract, which acts as both a reducing and stabilizing agent in the reaction with silver nitrate salt. This method is noted for its eco-friendly and non-toxic nature, diverging from more traditional chemical synthesis methods that often involve hazardous substances. The synthesized nanoparticles were confirmed to be of pure silver through UV-VIS and EDX analyses, with sizes ranging from 1 to 100 nm as shown by SEM analysis. This size range is particularly relevant for biomedical applications, where smaller particles can exhibit unique properties beneficial for therapy and diagnostics. The use of distilled water, n-Hexane, and Chloroform as solvents in the synthesis process was explored, aiming to understand the effect of different solvents on the properties of the synthesized nanoparticles. The significance of this approach lies in its contribution to the field of green nanotechnology, providing a sustainable method to produce nanoparticles that are potentially less harmful to the

environment and living organisms. *Asplenium dalhousiae* leaf extract not only plays a critical role in the synthesis of AgNPs but also exhibits substantial antioxidant properties. This is demonstrated by its ability to scavenge DPPH radicals, a common assay used to measure antioxidant capacity. This antioxidant activity is significant as oxidative stress is a known contributor to various diseases, including cancer, and antioxidants can help mitigate these effects. Furthermore, the study investigates into the biological activities of the *Asplenium dalhousiae* leaf extract and synthesized AgNPs, particularly focusing on their enzymatic inhibition and anticancer properties. The α-amylase inhibitory effect, which exhibits a concentration-dependent manner, highlights the potential therapeutic applications in managing conditions such as diabetes, where slowing down the breakdown of starch can be beneficial. The most striking finding of this research is the enhanced cytotoxic effect against human ovarian cancer cells when combining *Asplenium dalhousiae* leaf extract with synthesized AgNPs. This suggests that specific compounds within the leaf extract may synergistically interact with the silver nanoparticles, enhancing their ability to target and kill cancer cells. This observation is crucial as it opens new avenues for cancer treatment, where the goal is to develop therapies that are not only effective but also selective, and minimizing harm to healthy cells. This study not only advances the field of green nanotechnology by demonstrating an eco-friendly synthesis of silver nanoparticles but also illustrates the multifaceted potential of *Asplenium dalhousiae* leaf extract in therapeutic applications, ranging from antioxidant and enzymatic inhibition to potent anticancer activities. The integration of plant extracts with nanotechnology, as shown in this study, could lead to the development of novel therapeutic agents that are both effective and environmentally sustainable.

Declaration of Competing Interest

The authors declare no apparent competing financial interests or personal relationships that could have affected the reported work.

Data availability

Data will be made available on request.

Acknowledgements

The authors express their appreciation to the Deanship of Scientific Research at King Khalid University, Saudi Arabia, for financial support for research group program under grant number RGP-2/232/45. The authors also express gratitude to Higher Education Commission (HEC) for their financial support through the PC-1 scholarship for NUST, Islamabad, and the IRSIP

scholarship for Warwick University, United Kingdom. for providing the necessary research facilities to complete this task.

References

1. Rajasekharreddy, P., & Rani, U. P. (2014). Biofabrication of Ag nanoparticles using *Sterculia foetida* L. seed extract and their toxic potential against mosquito vectors and HeLa cancer cells. *Materials Science and Engineering: C*, 39, 203-212. https://doi.org/10.1016/j.msec.2014.03.003.
2. Pradhan, N., Pal, A., & Pal, T. (2002). Silver nanoparticle catalyzed reduction of aromatic nitro compounds. *Colloids and Surfaces A: Physicochemical and Engineering Aspects*, 196(2-3), 247-257. https://doi.org/10.1016/S0927-7757(01)01040-8
3. Patel, A. C., Li, S., Wang, C., Zhang, W., & Wei, Y. (2007). Electrospinning of porous silica nanofibers containing silver nanoparticles for catalytic applications. *Chemistry of Materials*, 19(6), 1231-1238. https://doi.org/10.1021/cm061331z
4. Veisi, H., Moradi, S. B., Saljooqi, A., & Safarimehr, P. (2019). Silver nanoparticle-decorated on tannic acid-modified magnetite nanoparticles (Fe_3O_4-TA/Ag) for highly active catalytic reduction of 4-nitrophenol, Rhodamine B, and Methylene blue. *Materials Science and Engineering: C*, 100, 445-452. https://doi.org/10.1016/j.msec.2019.03.03
5. Das, G., Patra, J. K., Debnath, T., Ansari, A., & Shin, H. S. (2019). Investigation of antioxidant, antibacterial, antidiabetic, and cytotoxicity potential of silver nanoparticles synthesized using the outer peel extract of *Ananas comosus* (L.). *PLOS ONE*, 14(8). https://doi.org/10.1371/journal.pone.0220950
6. Patil, M. P., Singh, R. D., Koli, P. B., Patil, K. T., Jagdale, B. S., Tipare, A. R., & Kim, G. D. (2018). Antibacterial potential of silver nanoparticles synthesized using *Madhuca longifolia* flower extract as a green resource. *Microbial Pathogenesis*, 121, 184-189. https://doi.org/10.1016/j.micpath.2018.05.040
7. Tanase, A., Berta, L., Coman, N. A., Roșca, I., Man, A., Toma, F., & Mare, A. (2019). Investigation of in vitro antioxidant and antibacterial potential of silver nanoparticles obtained by biosynthesis using beech bark extract. *Antioxidants*, 8(10), 459. https://doi.org/10.3390/antiox8100459
8. Chokshi, K., Pancha, I., Ghosh, T., Paliwal, C., Maurya, R., Ghosh, A., & Mishra, S. (2016). Green synthesis, characterization, and antioxidant potential of silver nanoparticles biosynthesized from de-oiled biomass of thermotolerant oleaginous microalgae *Acutodesmus dimorphus*. *RSC Advances*, 6(76), 72269-72274. https://doi.org/10.1039/C6RA15322D.
9. Erdogan, O., Abbak, M., Demirbolat, G. M., Birtekocak, F., Aksel, M., Pasa, S., & Cevik, O. (2019). Green synthesis of silver nanoparticles via *Cynara scolymus* leaf extracts: The characterization, anticancer potential with photodynamic therapy in MCF7 cells. *PLOS ONE*, 14(6). https://doi.org/10.1371/journal.pone.0216496.
10. Ahmad, A., Wei, Y., Ullah, S., Shah, S. I., Nasir, F., Shah, A., Iqbal, Z., Tahir, K., Khan, U. A., & Yuan, Q. (2017). Synthesis of phytochemicals-stabilized gold nanoparticles and their biological activities against bacteria and *Leishmania*. *Microbial Pathogenesis*, 110, 304-312. https://doi.org/10.1016/j.micpath.2017.07.009
11. Kolya, H., Maiti, P., Pandey, A., & Tripathy, T. (2015). Green synthesis of silver nanoparticles with antimicrobial and azo dye (Congo red) degradation properties using *Amaranthus gangeticus* Linn leaf extract. *Journal of Analytical Science and Technology*, 6(1), 33. https://doi.org/10.1186/s40543-015-0074-1.

12. Shehzad, A., Qureshi, M., Jabeen, S., Ahmad, R., Alabdalall, A. H., Aljafary, M. A., & Al-Suhaimi, E. (2018). Synthesis, characterization, and antibacterial activity of silver nanoparticles using *Rhazya stricta*. *PeerJ*, 6, e6086. https://doi.org/10.7717/peerj.6086
13. Li, S., Shen, Y., Xie, A., Yu, X., Qiu, L., Zhang, L., & Zhang, Q. (2007). Green synthesis of silver nanoparticles using *Capsicum annuum* L. extract. *Green Chemistry*, 9(8), 852-858. https://doi.org/10.1039/B615357G.
14. Hemmati, S., Rashtiani, A., Zangeneh, M. M., Mohammadi, P., Zangeneh, A., & Veisi, H. (2019). Green synthesis and characterization of silver nanoparticles using *Fritillaria* flower extract and their antibacterial activity against some human pathogens. *Polyhedron*, 158, 8-14. https://doi.org/10.1016/j.poly.2018.10.049.
15. Bhagat, M., Anand, R., Datt, R., Gupta, V., & Arya, S. (2019). Green synthesis of silver nanoparticles using aqueous extract of *Rosa brunonii* Lindl and their morphological, biological, and photocatalytic characterizations. *Journal of Inorganic and Organometallic Polymers and Materials*, 29(3), 1039-1047. https://doi.org/10.1007/s10904-018-0994-5
16. Lengke, M. F., Fleet, M. E., & Southam, G. (2007). Biosynthesis of silver nanoparticles by filamentous cyanobacteria from a silver (I) nitrate complex. *Langmuir*, 23(5), 2694-2699. https://doi.org/10.1021/la0613124.
17. El-Rafie, M. H., Mohamed, A. A., Shaheen, T. I., & Hebeish, A. (2010). Antimicrobial effect of silver nanoparticles produced by fungal process on cotton fabrics. *Carbohydrate Polymers*, *80*(3), 779-782. https://doi.org/10.1016/j.carbpol.2009.12.028
18. Logeswari, P., Silambarasan, S., & Abraham, J. (2015). Synthesis of silver nanoparticles using plant extract and analysis of their antimicrobial property. *Journal of Saudi Chemical Society*, *19*(3), 311-317. https://doi.org/10.1016/j.jscs.2012.04.007
19. Anbu, P., Gopinath, S. C. B., Yun, H. S., & Lee, C. G. (2019). Temperature-dependent green biosynthesis and characterization of silver nanoparticles using balloon flower plants and their antibacterial potential. *Journal of Molecular Structure, 1177*, 302-309. https://doi.org/10.1016/j.molstruc.2018.09.075
20. Anwar, A., Perveen, S., Ahmed, S., Siddiqui, R., Shah, M. R., & Khan, N. A. (2019). Silver nanoparticle conjugation with thiopyridine exhibited potent antibacterial activity against *Escherichia coli* and further enhanced by copper capping. *Jundishapur Journal of Microbiology*, *12*(3), 74455. https://doi.org/10.5812/jjm.74455
21. Yousaf, A., Zafar, A., Ali, M., Bukhary, S. M., Manzoor, Y., Tariq, T., & Zehra, S. S. (2019). Intrinsic bio-enhancer entities of *Fagonia cretica* for synthesis of silver nanoparticles involves anti-urease, anti-oxidant, and anti-tyrosinase activity. *Advances in Bioscience and Biotechnology, 10*(12), 455-468. https://doi.org/10.4236/abb.2019.1012032
22. Mohanta, Y. K., Biswas, K., Panda, S. K., Bandyopadhyay, J., De, D., Jayabalan, R., & Mohanta, T. K. (2017). Phyto-assisted synthesis of bio-functionalized silver nanoparticles and their potential antioxidant, antimicrobial, and wound healing activities. *IET Nanobiotechnology, 11*(8), 1027-1034. https://doi.org/10.1049/iet-nbt.2017.0017
23. Kiruba Daniel, S. G. C., Tharmaraj, V., Sironmani, T. A., & Pitchumani, K. (2010). Toxicity and immunological activity of silver nanoparticles. *Applied Clay Science, 48*(4), 547-551. https://doi.org/10.1016/j.clay.2010.03.001
24. Zhang, K., Liu, X., Ravi, S. O. A. S., Ramachandran, A., Ibrahim, I. A., Nassir, A. M., & Yao, J. (2019). Synthesis of silver nanoparticles (AgNPs) from leaf extract of *Salvia miltiorrhiza* and its anticancer potential in human prostate cancer LNCaP cell lines. *Artificial Cells, Nanomedicine, and Biotechnology, 47*(1), 2846-2854. https://doi.org/10.1080/21691401.2019.1638792
25. Banerjee, K., Choudhury, S. D. P., Ghosh, S., Baral, R., & Choudhuri, S. K. (2017). A novel approach of synthesizing and evaluating the anticancer potential of silver oxide nanoparticles in vitro. *Chemotherapy, 62*(5), 279-289. https://doi.org/10.1159/000453446

26. Thirumurugan, G., Shaheedha, S. M., & Dhanaraju, M. D. (2009). In vitro evaluation of antibacterial activity of silver nanoparticles synthesized by using *Phytophthora infestans*. *International Journal of ChemTech Research, 1*(3), 714-716.
27. McNamara, J. O. (2001). Drugs effective in the therapy of the epilepsies. New York: McGraw-Hill.
28. Bisht, A. S. (2015). Pharmacognostical and phytochemical evaluation of whole plant of *Asplenium dalhousiae* hook (fern). *World Journal of Pharmaceutical Research, 4*(9), 2186-2191.
29. Banerjee, K., Choudhury, S. D. P., Ghosh, S., Baral, R., & Choudhuri, S. K. (2017). A novel approach of synthesizing and evaluating the anticancer potential of silver oxide nanoparticles in vitro. *Chemotherapy, 62*(5), 279-289. https://doi.org/10.1159/000453446
30. Maher, S., Zamina, B., Riaz, M., Riaz, S., N. K., Imran, M., Fahmid, S., Ishtiaq, H., & Parveen, S. (2023). Green synthesis of *Withania coagulans* extract-mediated zinc oxide nanoparticles as photocatalysts and biological agents. *ACS Omega, 8*(49), 46715-46727. https://doi.org/10.1021/acsomega.3c05947
31. Hu, G., Liang, G., Zhang, W., Jin, W., Zhang, Y., Chen, Q., & Zhang, W. (2018). Silver nanoparticles with low cytotoxicity: controlled synthesis and surface modification with histidine. *Journal of Materials Science, 53*(7), 4768-4780. https://doi.org/10.1007/s10853-017-1940-6
32. Oboh, G., Akinyemi, A. J., & Ademiluyi, A. O. (2012). Inhibition of α-amylase and α-glucosidase activities by ethanolic extract of *Telfairia occidentalis* (fluted pumpkin) leaf. *Asian Pacific Journal of Tropical Biomedicine, 2*(9), 733–738. https://doi.org/10.1016/S2221-1691(12)60219-6
33. Nasir, G. A., Mohammed, A. K., & Samir, H. F. (2016). Biosynthesis and characterization of silver nanoparticles using olive leaves extract and sorbitol. *Iraqi Journal of Biotechnology, 15*(1), 22–32.
34. Devanesan, S., & AlSalhi, S. M. (2021). Green synthesis of silver nanoparticles using the flower extract of *Abelmoschus esculentus* for cytotoxicity and antimicrobial studies. *International Journal of Nanomedicine, 14*, 3343-3356. https://doi.org/10.2147/IJN.S307676
35. Ghani, S., Rafiee, B., Bahrami, S., Mokhtari, A., Aghamiri, S., & Yarian, F. (2022). Green synthesis of silver nanoparticles using the plant extracts of *Vitex agnus-castus L*: An eco-friendly approach to overcome antibiotic resistance. *International Journal of Preventive Medicine, 13*, 133. https://doi.org/10.4103/ijpvm.ijpvm14022
36. Mehta, B. K., Chhajlani, M., & Shrivastava, B. D. (2017). Green synthesis of silver nanoparticles and their characterization by XRD. *Frontiers of Physics and Plasma Science Journal of Physics, 836*(1), 012050. https://doi.org/10.1088/1742-6596/836/1/012050
37. Bewick, V., Cheek, L., & Ball, J. (2004). Statistics review 9: One-way analysis of variance. *Critical Care, 8*(2), 130-136. https://doi.org/10.1186/cc2836
38. Wickramaratne, M. N., Gunatilake, L. P., Anuradha, N. G. D., Godavillathanna, A. N., Perera, M. G. N., & Nicholas, I. (2015). Antioxidant activity and antibacterial activity of *Walidda antidysenterica*. *Journal of Pharmacognosy and Phytochemistry, 4*(2), 121-126.
39. Mittal, K., Tripathy, D., Choudhary, A., Aili, P. K., Chatterjee, A., & Singh, I. P. (2015). Bio-synthesis of silver nanoparticles using *Potentilla fulgens* Wall. ex Hook. and its therapeutic evaluation as anticancer and antimicrobial agent. *Materials Science and Engineering: C, 53*, 120-127. https://doi.org/

Click here to access/download;Manuscript;Final paper. PLos one.doc

Biosynthesis and Characterization of Silver Nanoparticles from *Asplenium dalhousiae* and their Potential Biological Properties

Shafia Parveen,[1] Shazia Iqbal,[2] Saima Maher,*[1] Paul Wilson,[3] Habib Nasir,[4] Shazia Nisar,[5] Muhammad Imran,[6] Musarat Riaz,[1] Rabail Urooj,[7] Mah Ganj Bakhshi,[1] Maria Essa,[1] Farah Mukhtar,[1] Attya Bhatti,[8] Hussnain A Janjua[8], Amir Faisal,[9] Arsalan Saleem,[10]

[1]Department of Chemistry, Sardar Bahadur Khan Women University Quetta.
[2]Department of Chemistry, Baluchistan University of Information Technology, Engineering and Management Sciences.
[3]Department of Chemistry, Warwick University, United Kingdom
[4] Department of Chemistry, School of Natural Sciences, National University of Sciences and Technology, H-12 Islamabad 44000, Pakistan.
[5] Department of Chemistry, University of Karachi, Pakistan,
[6]Department of Chemistry, Faculty of Science, King Khalid University, P.O. Box 9004, Abha 61413, Saudi Arabia.
[7]Department of Environmental Science, Sardar Bahadur Khan Women University Quetta
[8] Department of Industrial Biotechnology, Atta-ur-Rahman School of Applied Biosciences (ASAB), National University of Sciences and Technology, H-12 Islamabad 44000, Pakistan.
[9] Department of Biology, Lahore University of Management Sciences, D.H.A. Lahore Cantt. 54792, Pakistan.
[10]Department of Biological Sciences, COMSATS University, Islamabad 45550, Pakistan

Corresponding author: saimamaher@yahoo.com

Abstract

This study investigates the synthesis of silver nanoparticles (AgNPs) using the medicinal plant *Asplenium dalhousiae*, exploring its bioactive chemical constituents for their potential as reducing agents. Aqueous, chloroform, and *n*-hexane extracts of the plant leaves were utilized in the nanoparticle synthesis process. The successful formation of AgNPs was confirmed through UV-visible spectroscopy, which exhibited absorption peaks at approximately 420 nm, 443 nm, and 439 nm. Fourier-transform infrared (FTIR) spectroscopy was employed to identify functional groups in the plant extracts that facilitate reduction. The morphological and structural characteristics of the synthesized nanoparticles were analyzed using scanning electron microscopy (SEM) and X-ray diffraction (XRD), revealing predominantly spherical AgNPs with a crystalline structure and an average diameter of 46.98 ± 12.45 nm. Stability assessments through zeta potential (ZP) and dynamic light scattering (DLS) measurements yielded values of -44.5 mV and 231.8 nm, respectively, indicating favorable physical stability. The antibacterial efficacy of the synthesized AgNPs was evaluated against *Bacillus subtilis* and *Escherichia coli*, demonstrating significant inhibition at a concentration of 30 µg/ml. The highest zones of inhibition recorded were 23.0 ± 1.2 mm for *E. coli*, 22.0 ± 1.2 mm for *B. subtilis*, and 23 ± 1.6 mm for *Pseudomonas aeruginosa*. Additionally, the α-amylase inhibitory activity of *Asplenium dalhousiae* leaf extract exhibited a concentration-dependent response, with a maximum inhibition percentage of 90.84% at a concentration of 500 µg/ml. Antioxidant potential was assessed using the DPPH method, confirming that the synthesized AgNPs possess significant antioxidant properties alongside their antibacterial activity. The antibacterial activity of

Asplenium dalhousiae extract and synthesized AgNPs exhibited highest activity at the concentration of 30 µg/ml. The *Escherichia coli* (23.0 ± 1.2 mm) and *P. aeroginosa* (23 ± 1.6 mm) found the maximum zone of inhibition, compare to *B. subtilis* (22.0 ± 1.2 mm), The silver nanoparticles (AgNPs) from *Asplenium dalhousiae* confirmed significant α-amylase inhibition, with 85.04% inhibition at 500 µg/ml, as Acarbose (90.84%) and greater the leaf extract (78.65%). The aqueous and n-hexane silver nanoparticles (AgNPs) showed strong cytotoxic activity with low IC50 values, particularly in A2780 cells (15.76 µg/ml and 9.11 µg/ml, respectively), while the plant methanolic extract and CHCl3 AgNPs exhibited much higher IC50 values, indicating moderate to low activity. These findings highlight the promising role of integrating natural products with modern nanotechnology in developing novel therapeutic agents, particularly for cancer treatment. This innovative approach not only repurposes traditional medicinal plants but also enhances the functional capabilities of nanomaterials in medical applications, paving the way for improved treatment outcomes in patients with ovarian cancer and other diseases.

Keywords: Nanoparticles, *Asplenium dalhousiae*, Antibacterial, Antioxidant, α-amylase inhibitory,

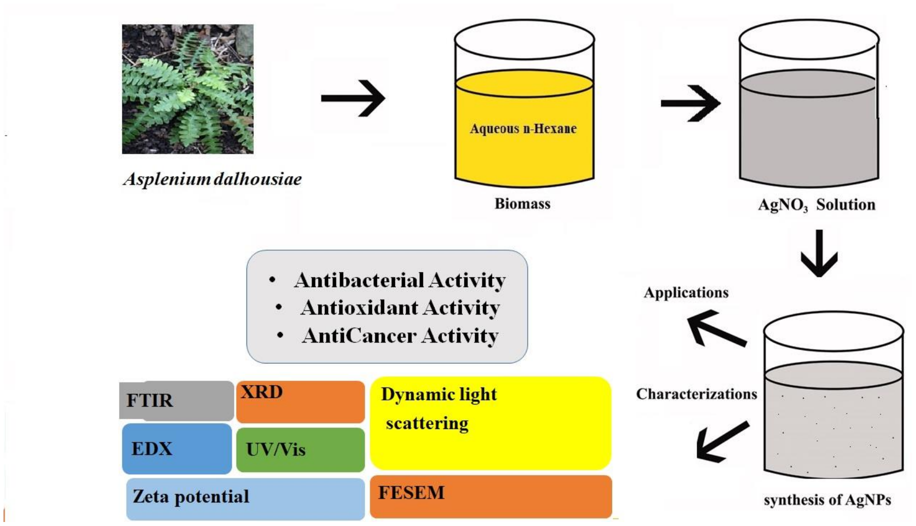

Graphical abstract.

Introduction

Nanotechnology, a rapidly advancing interdisciplinary field including chemistry, biomedical science, material science, diagnostics, therapeutics, and medicine, comprises the production and application of nanoscale materials with various properties [1]. Its key significance is highlighted by its broad uses, predominantly catalyzing organic toxin reduction in environmental perceptions [2,3,4]. Furthermore, nanotechnologies have vital biological properties, such as anti-bacterial [5,6]. Anti-oxidant [7,8], and anti-cancer activities [9]. Many inorganic metallic elements, comprising iron, copper, zinc, gold, silver, magnesium, and titanium, have been utilized in the biological, physical, and chemical synthesis of nanomaterials for a several determinations. Chemical methods are involved the use of harmful chemicals and energy intensive procedures, raising concerns about their environmental effect and the formation of toxic by-products. In compare, green synthesis methods deal a more sustainable resolution by utilizing bio-based materials, such as microorganisms, plants, as eco-friendly sources for nanoparticle synthesis. Many studies have confirmed that green synthesis not only reduces environmental harm but also

effectively produces nanoparticles with desirable characteristics. Silver nanoparticles (SNPs) play a important role in advanced research due to their nano medicinal prospective. [10,11]. The green method in nanotechnology presents several advantages, encompassing cost-effectiveness, environmental affability, high yield, and precipitate synthesis [12]. In this method, the synthesis of silver nanoparticles (SNPs) apply extracts from various plant parts [13,14,15,16,17]. SNPs are known for their biocompatibility and are important in the medical and pharmaceutical domains [18]. such as antibacterial activities [19,20], antioxidant qualities [21,22], immunological advantages [23], and anticancer activity, have been identified by researchers. Silver nanoparticles have been proposed recently for the development of novel anticancer drugs due to their broad spectrum of biological activity [[9], [10], [11]]. Nanoparticles are far more dangerous to cancerous cells than bulk materials. The potential use of nanoparticles as therapeutic agents for cancer cell detection and management are rapidly growing. Moreover, drugs can be designed to release in a controlled manner at a specific site as nanoparticles. AgNPs have garnered important attention for their potential anticancer properties, due to their capability to inhibit the production of malignant cells. This inhibition is supposed to stem from their interference with various signaling pathways involved in cancer development and metastasis. Research indicates that AgNPs can exert cytotoxic effects on cancer cells, thereby hindering tumor growth and progression. In addition to their anticancer capabilities, medicinal plants have gained prominence for their therapeutic benefits, mostly in regions like Pakistan, where traditional medicine depend on greatly on plant-based remedies. The adverse effects associated with synthetic pharmaceuticals such as hypertension, hormonal imbalances, and weight gain have led to a resurgence in the use of natural products for medicinal purposes. A current study focused on the efficient exploration of *Asplenium dalhousiae*, a well-known medicinal plant. This research represents the first comprehensive investigation of this species, both within Pakistan and globally. highlighting its potential for synthesizing silver nanoparticles. The flowering period of *Asplenium dalhousiae* spans from July to December, thriving in moist environments typical of temperate and subtropical regions. The study developed extracts from *Asplenium dalhousiae* to synthesize AgNPs and subsequently evaluated their antibacterial and antioxidant properties, α-amylase enzyme assay, Anticancer activity. Despite the plant's recognized biological significance, its full potential remains underexplored. Previous reports have noted its antibacterial and antifungal activities, as well as anti-fertility effects observed in adult male rats. This pioneering research aims to expand knowledge regarding the biological applications of Asplenium dalhousiae, particularly in the synthesis of silver nanoparticles and their associated health benefits. This study contributes valuable insights into the sustainable production of nanoparticles using natural resources, aligning with growing interests in green chemistry and eco-friendly practices in nanotechnology.

Material and Method

Sample collection, Identification and Analysis

The selected plant for this research, *Asplenium dalhousiae,* was collected from the Valley of Channat Bagh site in Azad Kashmir. Taxonomic identification was carried out by Dr. Amir of NARC Islamabad. The collected plant material was washed with distill water and then shade-drying, followed by uniform grinding the powder material was kept in refrigerator for further procedure. The subsequent scientific inquiries transpired within the well-equipped laboratories of the School of Natural Sciences (SNS), NUST. This academic institution played a pivotal role in facilitating various facets of the research. Characterization procedures took place in the SEME

unit of the Energy department at SNS, while antibacterial assessments were carried out at IESE NUST and ASAB NUST.

Preparation of plant extracts *(Asplenium dalhousiae)*

The 500g of powdered dried plant material soaked in 100% methanol for 15 days at normal temperature [30]. The procedure was followed by filtration with help of 125 mm pore size Whatman filter paper. The filtrate was placed in rotatory evaporator to evaporate at 45^0C. The gummy crude extract were dissolved and suspended in distilled water (140 ml) and successively portioned with different organic solvent such as n-Hexane (3×200ml), $CHCl_3$ (3×200ml) and a water layer (3×200ml).

Preparation of Ag-NPs

The AgNO3 solution was prepared using a accurate measurement of 100mg/100ml in deionized water. The solution was subjected in oil bath for 24 hrs. The prepared solution was centerifugated at 4000 rpm for 25min to collect the silver nanoparticles. The prepared silver nanoparticles were washed with distill water three times and vacuum dried for a night at 60 ^{0}C. The sample was re-suspended with distilled water for characterization.

Experimental and Characterization of Silver Nanoparticles

UV-Vis spectroscopy plays an important role for examining the reduction of silver ions to silver nanoparticles. The UV spectra of *Asplenium dalhousiae* plant extract were taken at different concentrations the range was 250 mL to 650 mL in 10 mL AgNO3 solution. UV-2800, BMS model was used in this research. The spectra of AgNps were recorded between 300 and 800 nm. The UV-Vis spectrum demonstrates the surface Plasmon resonance (SPR) absorption band of AgNps nanoparticles, showing a gradual decrease in absorbance with a shift in wavelength and changes in band width. This finding underscores the significance of the reduction of silver ions to silver nanoparticles and characterizing the silver optical properties of silver nanoparticles and structural features. **Fourier Transform Infrared (FTIR),** is used to recognize the functional group existent in plant. This technique, highlighting the role of bioactive plants chemicals in reduction and capping processes. The IR spectra were recorded with support of Perklin Elmer spectrum 100 models. **X-ray diffraction** is a very advantageous technique to determine the crystalline nature structure and characterization of synthesized nanoparticles through X-ray diffractogram. The instrumental model was used XRD; D8 Advance; Bruker, Billerica, MA, USA. **Scanning Electron Microscopy (SEM),** the morphology and size of synthesized silver nanoparticles (Ag-NPs) were examined using the JEOL-6490A-JSM (SEM). **Energy dispersion**

X-ray (EDX) was employed to acquire elemental composition of synthesized silver nanoparticles.

Preparation of Asplenium dalhousiae Extract and Ag-NPs for Antibacterial Activity

The plant extract was prepared, including n-Hexane, chloroform and, water extract, along with AgNO3 and Ag-NPs aqueous, chloroform, and *n*-Hexane layers, were weighed at 10mg/10ml and dissolved in sterile distilled water for Ag-NPs synthesis, 1mM silver nitrate solution was dissolve with plant extract to make a solution of 100 ml, and centrifuged at 4,000 rpm for 25min. The collected obtained pellets were used. The disc diffusion method was applied for antibacterial activity. [31]. Amoxicillin+Clavuanic acid was used as standard solution. The nutrient agar was prepared by suspending 28.0 grams in 1000 ml of distilled water and sterilizing it by autoclaving at 15 Ibs pressure (121°C) for 15 minutes. The agar was then mixed well and poured into prepared sterile petri plates. *Asplenium dalhousiae* leaves extracts and its synthesized silver nanoparticles were studied. The Gram-positive bacteria and Gram-negative bacteria strains were used in the biological assays. Nutrient agar was spread on sterilizes petri plates suspended in about 10ml of physiological saline in a Roux bottle and was incubated at 37ºC for 24 hours. The preparation of antibiotic sensitivity discs involves using Whatman No. 1 filter paper to create discs approximately 1mm in diameter. These discs are then sterilized by placing them in hot air. After sterilization, each disc is loaded with 30µl of the sample extract, silver nanoparticles (AgNPs), and a standard solution such as Amoxicillin+Clavuanic acid (30mg/ml distilled water). Subsequently, the loaded discs are refrigerated for 24 hours before being used for experiments.

Microorganisms

The antimicrobial assay of Ag nanoparticles was examined against The following bacteria were tested the Gram-negative *Escherichia coli* (ATCC 25922) and *Pseudomonas aeruginosa* (ATCC 27853), as well as the Gram-positive *Bacillus subtilis* (ATCC 13048). Nutrient agar was used and containing 50 ml of NA /PDA medium for bacteria. The potato dextrose agar was dried for 10 mints. The selected bacterial strains were inoculated in nutrient broth for the growth of microorganisms.

Evaluation of Anti-oxidant activity by DPPH assay

The antioxidant assay of plant three extracts a (*n*-hexane, chloroform and aqueous, extracts) and synthesized nanoparticles were evaluated by methanolic DPPH assay. This assay is constructed on the reduction of a DPPH in the presence of a reducing agent or antioxidant, the result of this reaction is the development of a stable non-radical DPPH-H molecule. The Ag-NPs solution was prepared in methanol at different concentrations the range were 10–100 µg/ml, and 200µl from

all dilution of silver nanoparticle solutions were added to 0.8 µl of DPPH solution. The samples were incubated in the dark for 30 minutes at room temperature, and the absorbance of each solution was read at 517 nm. Ascorbic acid was used as a positive control, and the antioxidant capacity to scavenge the DPPH radical was calculated as a percentage using the formula:

DPPH scavenging capacity (%) = [(A0 – A1) / A0] × 100

Where A0 is the absorbance of the control

A1 is the absorbance of the sample [1].

In-vitro α-amylase enzyme assay

The inhibition of *in-vitro* α-amylase enzyme assay was performed by using *Asplenium dalhousiae* different extracts and silver nanoparticles (Ag-NPs) as well as standard (acarbose). The assay was constructed on the previously described method of (Bernfield), which measures the capability of the plant crude extracts to inhibit α-amylase in a dose-dependent manner [32] .The results showed that *Asplenium dalhousiae* extract and Ag-NPs showed concentration dependent inhibition. The plant extracts showing a higher α-amylase of inhibitory effect compared comparatively to AgNPs. The IC_{50} value` for the extract and synthesized Ag-NPs were estimated, and the results specified that the extract had a lower IC_{50} value.

Anticancer activity

Anticancer activity against HCT116 and A2780 cell lines indicates to the capability of a compound to inhibit the growth and propagation of cancer cells derived from these specific cell lines. The efficiency of the constituent in inhibiting cancer cell growth is then calculated by measuring parameters such as cell viability, proliferation, apoptosis induction, and cell cycle capture. Substance representative anticancer activities against these cell lines have potential therapeutic implications for treating colorectal cancer (HCT116) and ovarian cancer (A2780), respectively. This activity is typically evaluated through *in vitro* assays where cancer cells are exposed to the substance of interest, such as the tested AgNPs, at various concentrations and exposure times.

Result and Discussion

UV-Vis spectroscopy

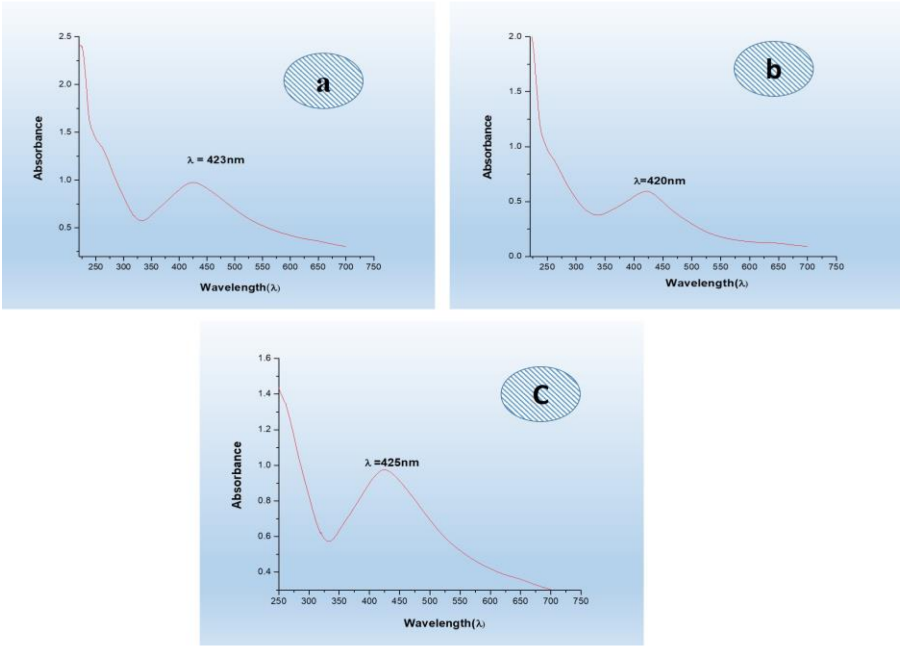

Fig 1. UV-Vis spectrograph of *Asplenium dalhousiae* in three selected layers.

The green synthesis of silver nanoparticles using the plant extract of *Asplenium dalhousiae* resulted in a color change from pale yellow to orange-brown, representing the formation of silver nanoparticles the UV-Vis results showing absorption peaks at 423 nm (aqueous extract), 420 nm (chloroform extract), and 425 nm (n-hexane extract). These absorption peaks support with previous reported data [33], of silver nanoparticles at 400 nm. Particularly, the broadening of the peak detected in spectra of the *n*-hexane as the higher concentration of silver nanoparticles it was compared with the other two extracts. Many studies have been revealed the use of different plant extracts, such as *A. esculentus* flowers, *Euphorbia wallichii*, were used to synthesize silver nanoparticles efficiently [34,35]. Overall, the green synthesis of silver nanoparticles from *Asplenium dalhousiae* extract showcases a promising method for producing nanoparticles.

Fourier transforms infrared spectroscopy

Fourier transformed infrared spectroscopy is used to identified the function group present in plant. It also very valuable to designates about molecular structure and type of chemical bonding which are responsible for the reduction of silver ion.

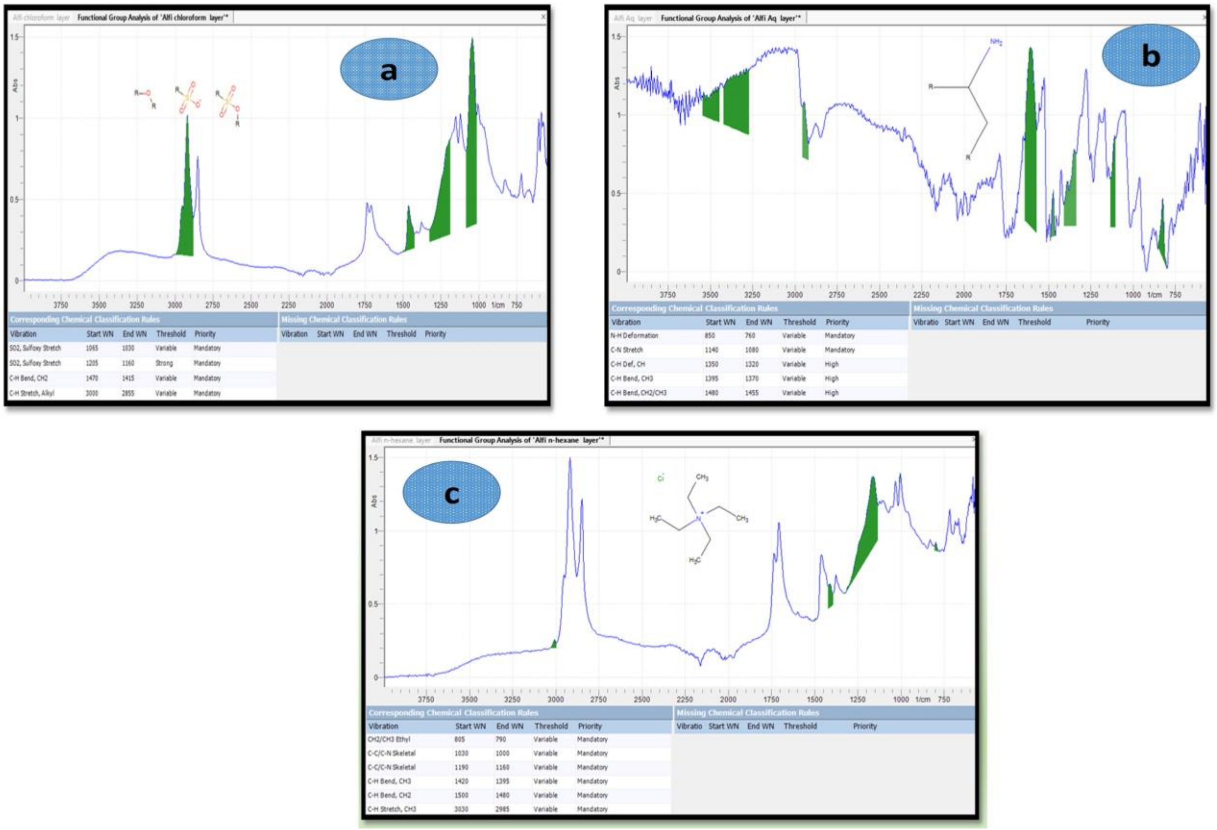

Fig 2: FTIR Spectrum of *Asplenium dalhousiae* in three selected layer. a) aqueous b) chloroform c) *n*-hexane extracts.

FT-IR study on plant extracts and AgNP samples indicate the presence of different functional groups in plant. The strong broad band around 3,400 cm^{-1} indicates the presence of alcoholic, phenolic, and carboxylic groups, predominantly O-H stretching of hydroxyl groups, N-H stretching vibrations of amines and amides, and water molecules. The C-N stretching vibration of aromatic amines is seen at 1,145 cm^{-1}, whereas the primary and secondary amines and amides are showed by bands between 900–600 cm^{-1}. Ketones, aldehydes, quinines, and esters are suggested by peaks between 1,700 and 1,600 cm^{-1} (C=O vibration). Additionally, C-H stretching of alkanes is observed at 2,920 cm^{-1}, C=C vibration of aromatic structures at 1,630 cm^{-1}, and C-O stretching of phenolic groups at 1,245 cm^{-1}. A broad band at 1,040 cm^{-1} indicates aromatic ethers and polysaccharides (C-O-C stretch). The FT-IR results confirm that the surface of AgNPs is covered by organic bioactive compounds consequent from the plant extracts *Asplenium dalhousiae* used in the study.

XRD analysis

The X-ray diffraction (XRD) analysis of the synthesized silver nanoparticles from *Asplenium dalhousiae* confirmed a crystalline nature, with the main phase being AgNPs and no evident impurities identified. The size of the Ag-NPs was determined to be 20nm using the Debye-Scherrer formula, which considers factors like X-ray wavelength, peak width, and Bragg angle. The XRD showed sharp peaks at 2θ values of 37.93°, 45.5°, 64.40°, and 77.08° corresponding to the crystallographic planes of face-centered cubic (fcc) silver crystals. [36]. the study confirmed that the silver nanoparticles were well-defined and had a consistent diffraction profile across all samples.

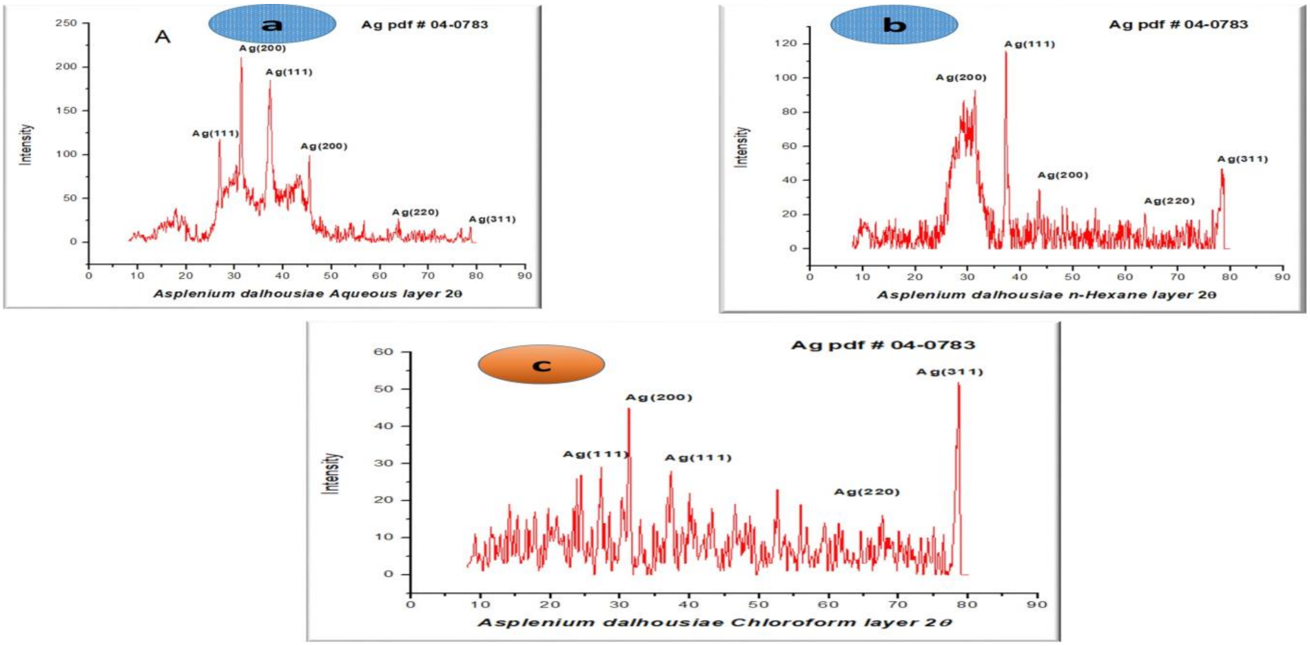

Fig 3. XRD result obtained from dried powdered of *Asplenium dalhousiae* plant extract, aqueous layer, b) *n*-Hexane layer, c) chloroform layer.

The Scanning Electron Microscope (SEM)

The Scanning Electron Microscope (SEM) study of the synthesized silver nanoparticles consequent from plant extracts provided important insights into their morphology and structure. This high-resolution imaging technique allowed for the detailed examination of the nanoparticle surfaces, revealing their size, shape, and distribution. The AgNPs synthesized in *n*-hexane showed better results compared to aqueous and chloroform extract. The particle size ranged from 1-100nm, with the SEM images displaying mostly spherical particles below 100 nm in size.

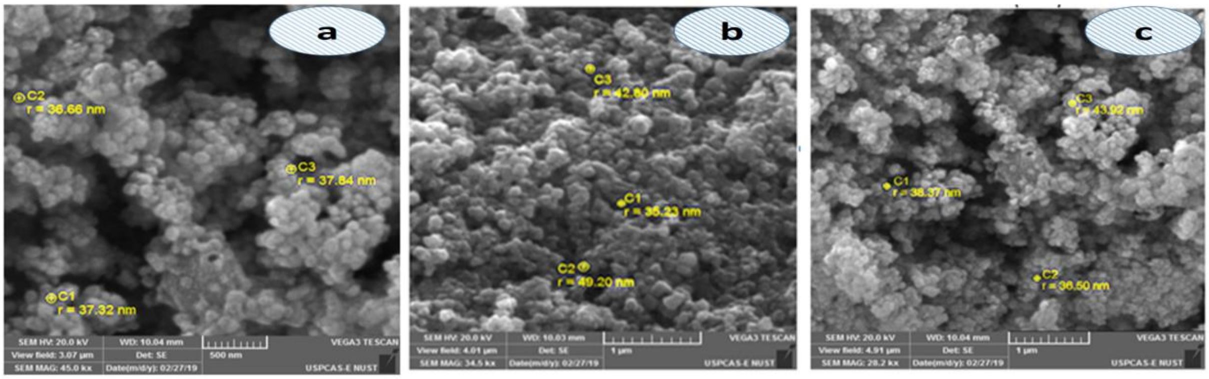

Fig 4. The SEM images of AgNps (*Asplenium dalhousiae*) plant extract, a) aqueous layer, b) *n*-Hexane layer, c) chloroform layer.

Energy Dispersive X-ray (EDX)

The EDX spectrum showed a strong peak corresponding to silver metal, confirming the presence of silver nanoparticles in the samples. The spectrum provided valuable information about the elemental composition of the nanoparticles, which is vital for understanding their properties and potential applications. EDX indicated the existence of silver (94%) and oxides, (6%) confirming the purity of nanoparticles.

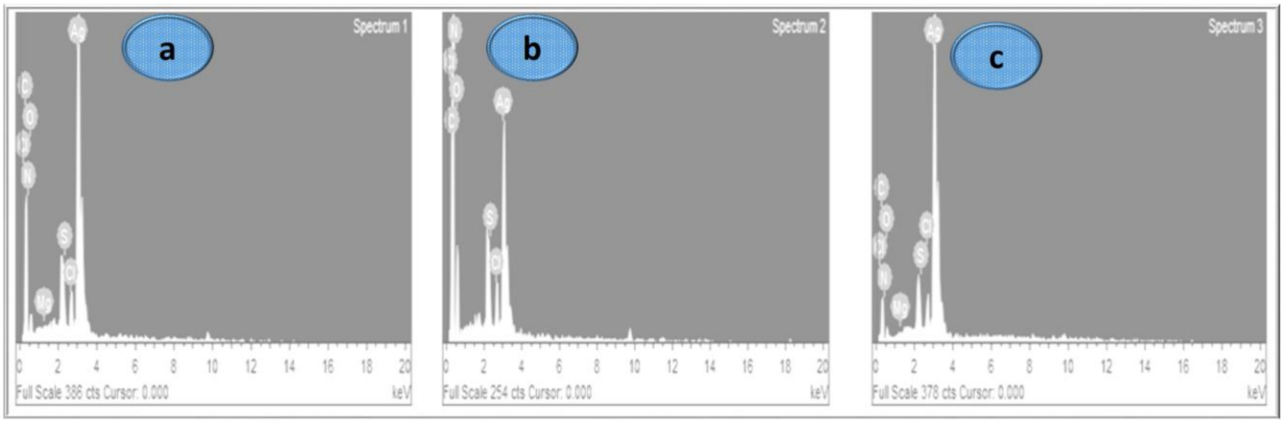

Fig 5. EDX of AgNps (A*splenium dalhousiae*) plant extract a) Aqueous layer, b) chloroform layer, c) *n*-hexane layer.

Antibacterial Activity of ApNPs and Statistical Analysis

The silver nanoparticles (AgNPs) showed anti-bacterial efficiency against both Gram-positive and Gram-negative bacteria. The statistical analysis involved expressing the results as Mean ± SD it is used to determine significant differences in each samples, a one-way analysis of variance (ANOVA) was conducted, followed by the Duncan's test for multiple comparisons, as outlined by Harvey and Paige (1998). [37]

The statistical analysis was carried out using Graph Pad Prism Software, version 5. A significance level of $p < 0.05$ was considered, indicating that results with a p-value below this

threshold were deemed statistically significant. This approach provided a comprehensive evaluation of the antibacterial activity of Ag-NPs and their effectiveness against different bacterial strains.

The antibacterial activity of *Asplenium dalhousiae* extract and synthesized AgNPs exhibited highest activity at the concentration of 30 µg/ml. The *Escherichia coli* (23.0 ± 1.2 mm) and *P. aeroginosa* (23 ± 1.6 mm) found the maximum zone of inhibition, compare to *B. subtilis* (22.0 ± 1.2 mm), and the synthesized AgNPs from the plant extract also exhibited the antibacterial activity against both Gram-positive and negative bacteria, the antibacterial activity were performed on dose-dependent. In highest concentration of AgNPs demonstrated the significance inhibitory effect on the growth of bacterial organisms.

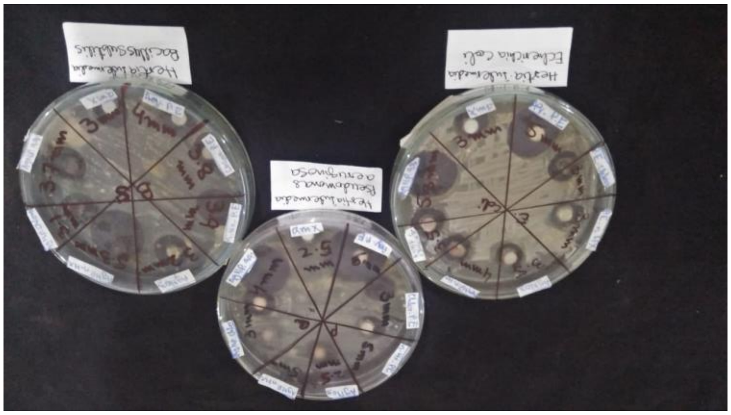

Fig 6: Zone of inhibition of Antibacterial Activity.

Table 1: Antibacterial activity of silver nanoparticles and plant extracts against selected bacterial

**Group format**	**Doses µg/ml**	***E. coli* (mm)**		***B. subtilis* (mm)**		***P. aeruginosa* (mm)**	
**Extracts**	**Mean**	**Mean**	**SD**	**Mean**	**SD**	**Mean**	**SD**
$AgNO_3$	30.	18.0	1.2	17.5	1.6	18.0	1.2
AgNP of $CHCl_3$ extract	30.	19.0	1.2	18.0	0.6	18.0	1.4
AgNP Aqueous extract	30.	19.5	1.4	17.5	1.6	17.0	1.1
AgNP *n*-Hexane layer	30.	20.0	1.8	19.0	1.2	19.5	1.4
Plant extract of $CHCl_3$ extract	30.	19.5	1	17.0	1.2	18.0	1.2
Plant extract of Aqueous layer	30.	16.0	1.2	15.0	1.6	16.5	1.2
Plant extract of *n*-Hexane layer	30.	17.0	1.2	15.0	0.6	15.0	1.2
Standard (Amoxycillin)	30.	23.0	1.2	22.0	1.2	23.0	1.6

strains.

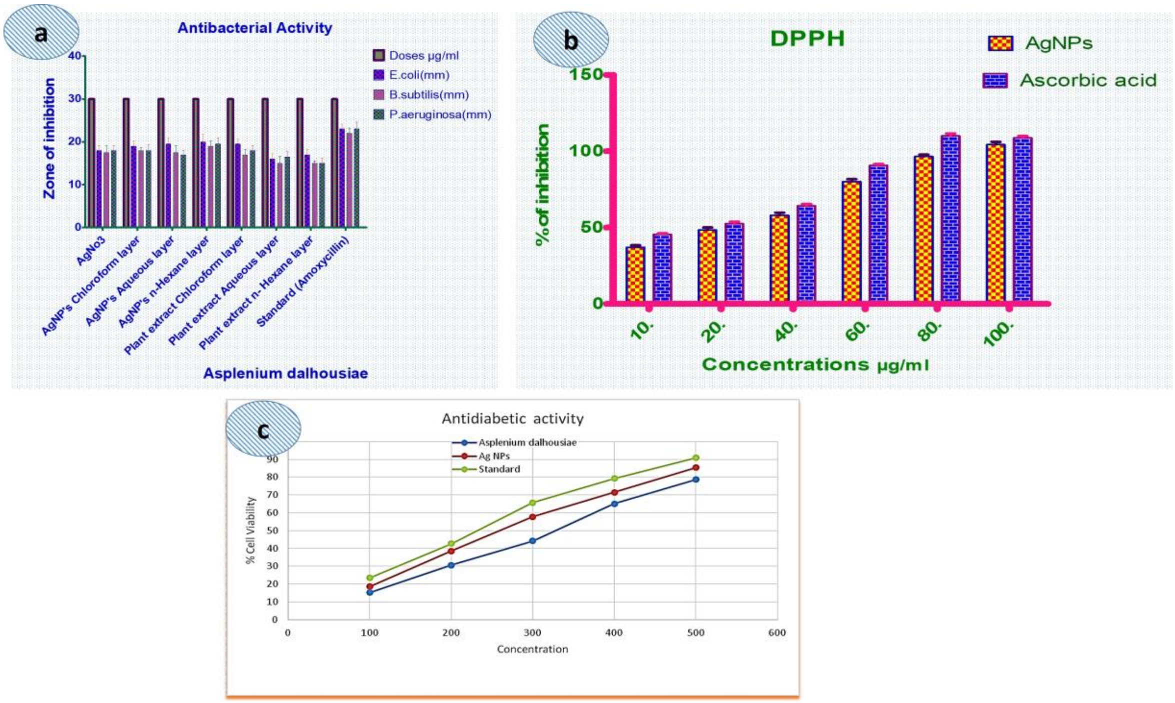

Fig 7: Graphical representation of DPPH, anti-bacterial, and anti-diabetic activity.

2,2-diphenyl-1-picrylhydrazyl (DPPH) radical Assay

DPPH is a highly stable and very well-known free radical. It is based on the reduction of accepting hydrogen or electron from donors. The DPPH reducing capability of the AgNPs was evaluated by detecting color change. The DPPH scavenging assay demonstrated effective inhibition activity of AgNPs when related with the standard (ascorbic acid). The ability of plant extracts to scavenge the 2, 2-diphenyl-1-picrylhydrazyl (DPPH) radical. The absorbance of the resultant solution was measured at a wavelength of 517 nm, the previously defined protocol was adopted with slightly modified [38]. The standard ascorbic acid was used as a positive control for comparison with the plant extract. The % of DPPH scavenging capability was measured using the following formula:

DPPH scavenging capacity (in percentage) = $[A^0 – A1/A^0] \times 100$

Where:

(A_0) represents the absorbance of the control (ascorbic acid).

(A_1) represents the absorbance of the sample (AgNPs)

Table 2: DPPH Radical Scavenging Capacity of Ascorbic Acid and (AgNPs)

**Concentrations**	**Ascorbic Acid (Mean ± SD)**	**AgNPs Mean ± SD**
10	45.69±0.6	37.19±1.2
20	52.54±1.4	48.45±1.6
40	64.27±1.2	58.06±1.8

60	90.77±0.8	85.12±1.6
80	110.06±1.6	96.56±1.2
100	108.77±1.2	104.38±1.8

Table 3: DPPH radical scavenging capacities of ascorbic acid and AgNPs.

**S. No**	**Concentration (μg/ml)**	**% Cell Viability**		
		**AgNPs**	**Ascorbic Acid**	**SD**
1	10	37.19	45.69	1.2
2	20	48.45	52.54	1.6
3	40	58.06	64.27	1.8
4	60	85.12	90.77	1.6
5	80	96.56	110.06	1.2
6	100	104.38	118.77	1.8
IC50 (μg/ml)		23.64374724	17.78071696	56.795

The results of this study specify that *Asplenium dalhousiae* extracts possess prominent antioxidant activity, as confirmed by their capability to scavenge DPPH radicals. The difference in IC_{50} values of extracts highlights the importance of polarity bases solvent in extracting bioactive chemical compounds with antioxidant ability. The highest DPPH scavenging activity of the crude methanol extract suggests that this solvent is particularly effective in extracting antioxidant compounds from *Asplenium dalhousiae*. But the aqueous extract showed lower activity. The observed differences in IC_{50} values among the solvent extracts could be attributed to variations in the types and concentrations of bioactive compounds present in each extract. Further investigation into the chemical composition of these extracts could provide insights into the specific antioxidant compounds responsible for their activity. These findings suggest that the antioxidant potential of *Asplenium dalhousiae* and emphasize the significance of solvent selection in optimizing the extraction of bioactive compounds for potential therapeutic applications.

Inhibition of in-vitro α-amylase enzyme assay

Alpha amylase inhibitors attach to the alpha bonds of polysaccharides like glycogen and starch, preventing their breakdown into glucose and maltose. These inhibitors play a vital role in several applications, and management human diseases. They work by reducing endogenous alpha amylase activity, thereby impeding the conversion of complex carbohydrates and disaccharides into absorbable monosaccharides. Clinical trials have shown that these inhibitors delay the digestion of complex carbohydrates and disaccharides, inhibiting α-glucosidases and impacting the absorption of glucose. Polyphenols found in dietary sources act as natural inhibitors of α-

amylase and α-glucosidase, slowing down glucose absorption by interfering with these key enzymes involved in carbohydrate digestion

Table 4: Inhibition of *in-vitro* α-amylase enzyme assay.

Groups	Concentration (µg/ml)	% Cell Viability		
		Asplenium dalhousiae	**Ag NPs**	**Standard**
Group I	100	15.25±1.01	18.58±1.30	23.45±1.25
Group II	200	30.55±1.50	38.5±2.08	42.61±2.45
Group III	300	44.16± 2.40	57.7±3.09	65.74±3.06
Group IV	400	65.05±3.01	71.44±3.80	79.31±3.70
Group V	500	78.65±4.02	85.4±4.09	90.84±4.56
IC50 (µg/ml)		320.26	274.04	239.40

The α-amylase inhibitors found in *Asplenium dalhousiae* leaf extract and silver nanoparticles (AgNPs) exhibit concentration-dependent inhibition of α-amylase activity. At 100 µg/ml concentration, the inhibition percentages were 15.25% for the leaf extract, 18.58% for AgNPs, and 23.45% for Acarbose. At a higher concentration of 500 µg/ml, the inhibition percentages increased significantly to 78.65% for the leaf extract, 85.04% for AgNPs, and 90.84% for Acarbose. The half inhibition concentration (IC_{50}) values for the α-amylase inhibition activity of Asplenium dalhousiae leaf extract, AgNPs, and Acarbose were 320.26 µg/ml for the leaf extract, 274.04 µg/ml for AgNPs, and 239.40 µg/ml for Acarbose. Notably, AgNPs demonstrated a substantial *in vitro* antidiabetic effect against α-amylase activity, with results comparable to the standard drug Acarboseare and then the leaf extract

Anticancer Activity

The AgNPs were tested for their anticancer activity against HCT116 and A2780 cell lines with the lowest IC_{50} concentration at 5, 10 and 30 µg/ml for various exposure times of 24, 48 and 72 h. Generally, green synthesized silver nanoparticles manifested the significant anticancer activity with toxic manner depending on the agents responsible for reducing and capping the silver nitrate into silver [39, 40].

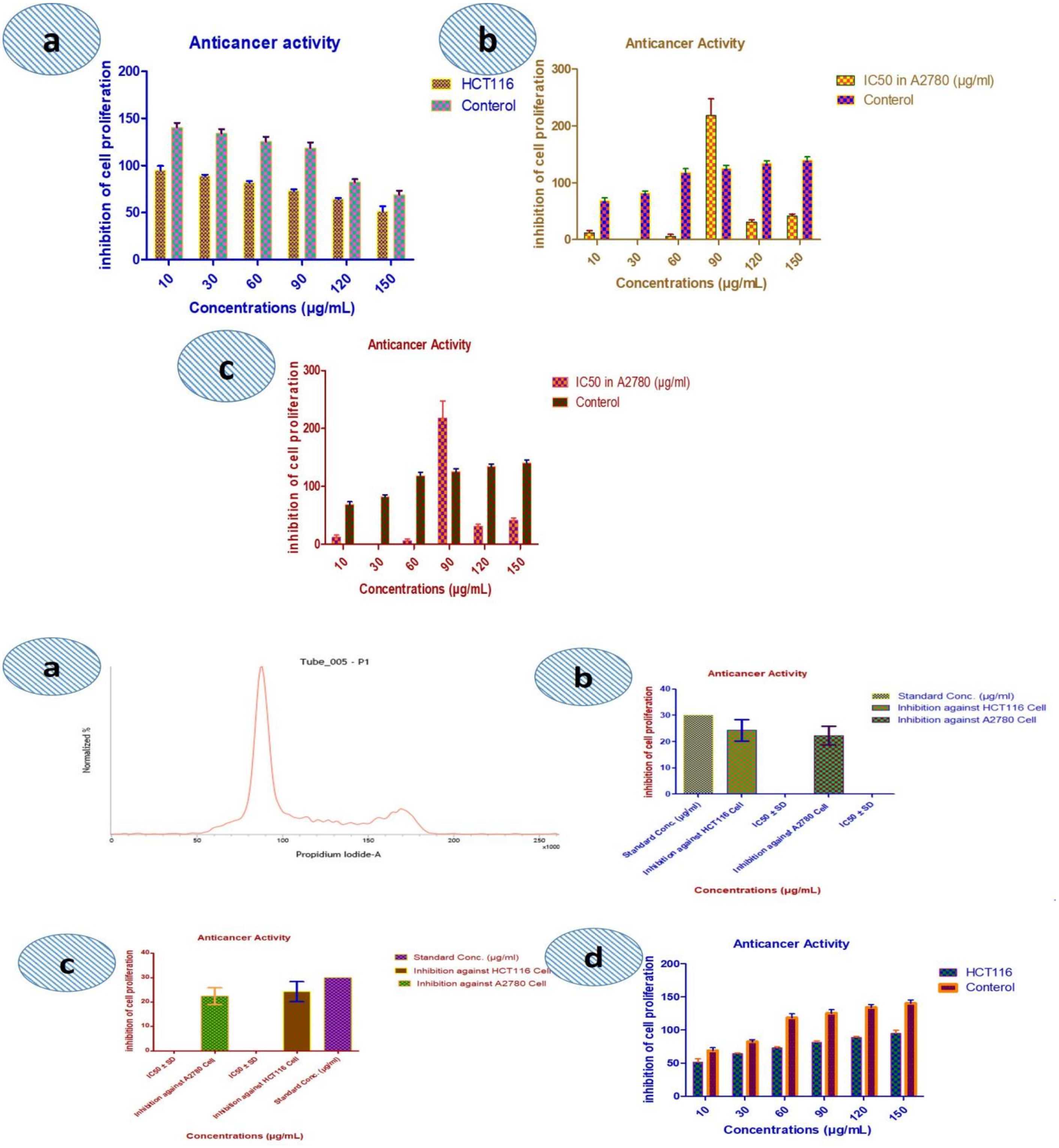

Fig 8: Graphical representation of the anticancer activity.

Propidium iodide (PI) is normally used along with Annexin V to evaluate cell viability of apoptosis and necrosis, on the bases of differences in plasma membrane integrity and permeability. propidium iodide (PI) gives information to the researchers about identify different types of cell death either necrosis or apoptosis. It is depending on two components, annexin V,

DNA-binding dye molecule PI, The annexin V, is a protein and binds with phospholipids and it is called phosphatidylserines, which generally occur in inner, cytoplasm fronting leaflet of a cell's membrane, but become "flipped" to the outer leaflet during the early stages of apoptosis. The DNA-binding dye molecule PI, which can only enter cells when their membranes are ruptured a characteristic of both necrosis and late apoptosis. [41].

Table 5: The anticancer activity of different samples against HCT116 and A2780 cancer cell line

**Sample I.D.** AgNPs	**Standard Conc. (µg/ml)**	**Inhibition against HCT116**		**Inhibition against A2780**	
		**Cell Viability (%)**	**IC_{50} ± SD**	**Cell Viability (%)**	**IC_{50} ± SD**
CF	30	25.5± 1.2	Active	23.2	Active
HF	30	27.7±0.6	Active	25.3	Active
AF	30	19.8±1.4	Active	18.5	Active

The *Asplenium dalhousiae* leaf extracts and silver nanoparticles (AgNPs) established a significant inhibition of capability and proliferation in both A2780 and HCT116 cells. The experimental results, stated as the mean standard deviation of three independent experiments, were examined using one-way ANOVA in the Origin program version 8.5. The inhibition data against HCT116 and A2780 cells for different samples, including, and $CHCl_3$ extracts were presented in Table 3. Notably, The anticancer activity at a concentration of 30 µg/ml. For HCT116 cells, the cell viability percentages were 25.5% (CF), 27.7% (HF), and 19.8% (AF), all classified as active. In A2780 cells, the viability percentages were 23.2% (CF), 25.3% (HF), and 18.5% (AF), indicating active inhibition across all samples. all samples were classified as "Active" in terms of their inhibition against the cell lines, indicating a substantial effect on cell viability and proliferation.

Table 6. *Asplenium dalhousiae* AgNPs inhibition against HCT116 and A2780.

**I.D No.**	**IC_{50} in HCT116 (µg/ml)**	**Sem**	**Replicate**	**IC_{50} in A2780 (µg/ml)**	**Sem**	**Replicate**
AgNP's aqueous	21.11	0.93	**2**	15.76	7.75	**2**
AgNP's, *n-h*exane,	45.98	29.56	**2**	9.11	2.55	**2**
AgNP's $CHCl_3$	605.2	258.3	**2**	247.66	188.64	**2**
Plant methanolic Extract	603.25	45.35	2	34.97	27.38	2
**Result**	Active					

The data suggests that the combination of *Asplenium dalhousiae* leaf methanolic extracts and synthesized silver nanoparticles (AgNPs of Aqueous, hexane and $CHCl_3$) exhibits potent apoptotic activity in human ovarian cancer cells. This combined treatment demonstrated a higher cytotoxic effect on ovarian cancer cells compared to the effect induced by AgNPs alone. The synergistic action of *Asplenium dalhousiae* leaf extracts and AgNPs presents a promising approach for enhancing the apoptotic activity in ovarian cancer cells, potentially offering a more effective treatment strategy. In the investigation of alternative treatments for human ovarian cancer, the application of *Asplenium dalhousiae* leaf extracts in conjunction with silver nanoparticles (AgNPs) has emerged as a notable area of study. The focus of this discussion is the observed potent apoptotic activity induced by this combined treatment in ovarian cancer cells, which underscores a significant advancement in the realm of cancer therapy. The study reveals that *Asplenium dalhousiae* leaf extracts are combined with AgNPs, the fabrication exhibits a remarkable ability to induce programmed cell death, or apoptosis, in ovarian cancer cells. This is particularly noteworthy because apoptosis is a crucial mechanism for eliminating cancer cells in a controlled manner, thereby preventing their proliferation. The fact that this combined treatment outperforms the application of AgNPs alone in inducing cytotoxic effects offers a promising avenue for enhancing the efficacy of cancer treatments. However, the novel finding that the addition of *Asplenium dalhousiae* leaf extract to AgNPs significantly increases the cytotoxic effect against ovarian cancer cells suggests that compounds within the leaf extract may play a pivotal role in enhancing the nanoparticles' ability to target and kill cancer cells. The IC_{50} values for HCT116 cells were 21.11 µg/ml for aqueous AgNPs (Sem 0.93), 45.98 µg/ml for n-hexane AgNPs (Sem 29.56), and 605.2 µg/ml for CHCl3 AgNPs (Sem 258.3). In A2780 cells, the IC_{50} values were 15.76 µg/ml for aqueous AgNPs (Sem 7.75), 9.11 µg/ml for n-hexane AgNPs (Sem 2.55), and 247.66 µg/ml for CHCl3 AgNPs (Sem 188.64). The plant methanolic extract displayed IC_{50} values of 603.25 µg/ml (Sem 45.35) for HCT116 and 34.97 µg/ml (Sem 27.38) for A2780. Overall, the results indicate significant anticancer activity, particularly from the aqueous and n-hexane AgNPs. This synergistic effect, where the combined treatment is more effective than the sum of its parts, indicates that the *Asplenium dalhousiae* leaf extract could be modulating the biological response of the cancer cells to AgNPs, possibly by making them more susceptible to apoptosis. Such an interaction between a natural plant extract and nanoparticles opens up new paths for the development of cancer therapies that are both more efficient and potentially less harmful to normal cells, given the targeted nature of the treatment.

Conclusion

This study presents an innovative approach to the green synthesis of silver nanoparticles (AgNPs) using *Asplenium dalhousiae* leaf extract, which acts as both a reducing and stabilizing agent in the reaction with silver nitrate salt. This method is noted for its eco-friendly and non-toxic nature, diverging from more traditional chemical synthesis methods that often involve hazardous substances. The synthesized nanoparticles were confirmed to be of pure silver through UV-VIS and EDX analyses, with sizes ranging from 1 to 100 nm as shown by SEM analysis. This size range is particularly relevant for biomedical applications, where smaller particles can exhibit unique properties beneficial for therapy and diagnostics. The use of distilled water, n-Hexane, and Chloroform as solvents in the synthesis process was explored, aiming to understand the effect of different solvents on the properties of the synthesized nanoparticles. The significance of this approach lies in its contribution to the field of green nanotechnology, providing a sustainable method to produce nanoparticles that are potentially less harmful to the environment and living organisms. *Asplenium dalhousiae* leaf extract not only plays a critical role in the synthesis of AgNPs but also exhibits substantial antioxidant properties. This is demonstrated by its ability to scavenge DPPH radicals, a common assay used to measure antioxidant capacity. This antioxidant activity is significant as oxidative stress is a known contributor to various diseases, including cancer, and antioxidants can help mitigate these effects. Furthermore, the study investigates into the biological activities of the *Asplenium dalhousiae* leaf extract and synthesized AgNPs, particularly focusing on their enzymatic inhibition and anticancer properties. The α-amylase inhibitory effect, which exhibits a concentration-dependent manner, highlights the potential therapeutic applications in managing conditions such as diabetes, where slowing down the breakdown of starch can be beneficial. The most striking finding of this research is the enhanced cytotoxic effect against human ovarian cancer cells when combining *Asplenium dalhousiae* leaf extract with synthesized AgNPs. This suggests that specific compounds within the leaf extract may synergistically interact with the silver nanoparticles, enhancing their ability to target and kill cancer cells. This observation is crucial as it opens new avenues for cancer treatment, where the goal is to develop therapies that are not only effective but also selective, and minimizing harm to healthy cells. This study not only advances the field of green nanotechnology by demonstrating an eco-friendly synthesis of silver nanoparticles but also illustrates the multifaceted potential of *Asplenium dalhousiae* leaf extract in therapeutic applications, ranging from antioxidant and enzymatic inhibition to potent anticancer activities. The integration of plant extracts with nanotechnology, as shown in this study, could lead to the development of novel therapeutic agents that are both effective and environmentally sustainable.

Declaration of Competing Interest

The authors declare no apparent competing financial interests or personal relationships that could have affected the reported work.

Data availability

Data will be made available on request.

Acknowledgements

The authors express their appreciation to the Deanship of Scientific Research at King Khalid University, Saudi Arabia, for financial support for research group program under grant number RGP-2/232/45. The authors also express gratitude to Higher Education Commission (HEC) for their financial support through the PC-1 scholarship for NUST, Islamabad, and the IRSIP scholarship for Warwick University, United Kingdom. for providing the necessary research facilities to complete this task.

References

1. Rajasekharreddy, P., & Rani, U. P. (2014). Biofabrication of Ag nanoparticles using *Sterculia foetida* L. seed extract and their toxic potential against mosquito vectors and HeLa cancer cells. *Materials Science and Engineering: C*, 39, 203-212. https://doi.org/10.1016/j.msec.2014.03.003.
2. Pradhan, N., Pal, A., & Pal, T. (2002). Silver nanoparticle catalyzed reduction of aromatic nitro compounds. *Colloids and Surfaces A: Physicochemical and Engineering Aspects*, 196(2-3), 247-257. https://doi.org/10.1016/S0927-7757(01)01040-8
3. Patel, A. C., Li, S., Wang, C., Zhang, W., & Wei, Y. (2007). Electrospinning of porous silica nanofibers containing silver nanoparticles for catalytic applications. *Chemistry of Materials*, 19(6), 1231-1238. https://doi.org/10.1021/cm061331z
4. Veisi, H., Moradi, S. B., Saljooqi, A., & Safarimehr, P. (2019). Silver nanoparticle-decorated on tannic acid-modified magnetite nanoparticles (Fe_3O_4-TA/Ag) for highly active catalytic reduction of 4-nitrophenol, Rhodamine B, and Methylene blue. *Materials Science and Engineering: C*, 100, 445-452. https://doi.org/10.1016/j.msec.2019.03.03
5. Das, G., Patra, J. K., Debnath, T., Ansari, A., & Shin, H. S. (2019). Investigation of antioxidant, antibacterial, antidiabetic, and cytotoxicity potential of silver nanoparticles synthesized using the outer peel extract of *Ananas comosus* (L.). *PLOS ONE*, 14(8). https://doi.org/10.1371/journal.pone.0220950
6. Patil, M. P., Singh, R. D., Koli, P. B., Patil, K. T., Jagdale, B. S., Tipare, A. R., & Kim, G. D. (2018). Antibacterial potential of silver nanoparticles synthesized using *Madhuca longifolia* flower extract as a green resource. *Microbial Pathogenesis*, 121, 184-189. https://doi.org/10.1016/j.micpath.2018.05.040
7. Tanase, A., Berta, L., Coman, N. A., Roșca, I., Man, A., Toma, F., & Mare, A. (2019). Investigation of in vitro antioxidant and antibacterial potential of silver nanoparticles obtained by biosynthesis using beech bark extract. *Antioxidants*, 8(10), 459. https://doi.org/10.3390/antiox8100459
8. Chokshi, K., Pancha, I., Ghosh, T., Paliwal, C., Maurya, R., Ghosh, A., & Mishra, S. (2016). Green synthesis, characterization, and antioxidant potential of silver nanoparticles biosynthesized from de-oiled biomass of thermotolerant oleaginous microalgae *Acutodesmus dimorphus*. *RSC Advances*, 6(76), 72269-72274. https://doi.org/10.1039/C6RA15322D.
9. Erdogan, O., Abbak, M., Demirbolat, G. M., Birtekocak, F., Aksel, M., Pasa, S., & Cevik, O. (2019). Green synthesis of silver nanoparticles via *Cynara scolymus* leaf extracts: The characterization, anticancer potential with photodynamic therapy in MCF7 cells. *PLOS ONE*, 14(6). https://doi.org/10.1371/journal.pone.0216496.
10. Ahmad, A., Wei, Y., Ullah, S., Shah, S. I., Nasir, F., Shah, A., Iqbal, Z., Tahir, K., Khan, U. A., & Yuan, Q. (2017). Synthesis of phytochemicals-stabilized gold nanoparticles and their biological activities against bacteria and *Leishmania*. *Microbial Pathogenesis*, 110, 304-312. https://doi.org/10.1016/j.micpath.2017.07.009

11. Kolya, H., Maiti, P., Pandey, A., & Tripathy, T. (2015). Green synthesis of silver nanoparticles with antimicrobial and azo dye (Congo red) degradation properties using *Amaranthus gangeticus* Linn leaf extract. *Journal of Analytical Science and Technology*, 6(1), 33. https://doi.org/10.1186/s40543-015-0074-1.
12. Shehzad, A., Qureshi, M., Jabeen, S., Ahmad, R., Alabdalall, A. H., Aljafary, M. A., & Al-Suhaimi, E. (2018). Synthesis, characterization, and antibacterial activity of silver nanoparticles using *Rhazya stricta*. *PeerJ*, 6, e6086. https://doi.org/10.7717/peerj.6086
13. Li, S., Shen, Y., Xie, A., Yu, X., Qiu, L., Zhang, L., & Zhang, Q. (2007). Green synthesis of silver nanoparticles using *Capsicum annuum* L. extract. *Green Chemistry*, 9(8), 852-858. https://doi.org/10.1039/B615357G.
14. Hemmati, S., Rashtiani, A., Zangeneh, M. M., Mohammadi, P., Zangeneh, A., & Veisi, H. (2019). Green synthesis and characterization of silver nanoparticles using *Fritillaria* flower extract and their antibacterial activity against some human pathogens. *Polyhedron*, 158, 8-14. https://doi.org/10.1016/j.poly.2018.10.049.
15. Bhagat, M., Anand, R., Datt, R., Gupta, V., & Arya, S. (2019). Green synthesis of silver nanoparticles using aqueous extract of *Rosa brunonii* Lindl and their morphological, biological, and photocatalytic characterizations. *Journal of Inorganic and Organometallic Polymers and Materials*, 29(3), 1039-1047. https://doi.org/10.1007/s10904-018-0994-5
16. Lengke, M. F., Fleet, M. E., & Southam, G. (2007). Biosynthesis of silver nanoparticles by filamentous cyanobacteria from a silver (I) nitrate complex. *Langmuir*, 23(5), 2694-2699. https://doi.org/10.1021/la0613124.
17. El-Rafie, M. H., Mohamed, A. A., Shaheen, T. I., & Hebeish, A. (2010). Antimicrobial effect of silver nanoparticles produced by fungal process on cotton fabrics. *Carbohydrate Polymers*, *80*(3), 779-782. https://doi.org/10.1016/j.carbpol.2009.12.028
18. Logeswari, P., Silambarasan, S., & Abraham, J. (2015). Synthesis of silver nanoparticles using plant extract and analysis of their antimicrobial property. *Journal of Saudi Chemical Society*, *19*(3), 311-317. https://doi.org/10.1016/j.jscs.2012.04.007
19. Anbu, P., Gopinath, S. C. B., Yun, H. S., & Lee, C. G. (2019). Temperature-dependent green biosynthesis and characterization of silver nanoparticles using balloon flower plants and their antibacterial potential. *Journal of Molecular Structure*, *1177*, 302-309. https://doi.org/10.1016/j.molstruc.2018.09.075
20. Anwar, A., Perveen, S., Ahmed, S., Siddiqui, R., Shah, M. R., & Khan, N. A. (2019). Silver nanoparticle conjugation with thiopyridine exhibited potent antibacterial activity against *Escherichia coli* and further enhanced by copper capping. *Jundishapur Journal of Microbiology*, *12*(3), 74455. https://doi.org/10.5812/jjm.74455
21. Yousaf, A., Zafar, A., Ali, M., Bukhary, S. M., Manzoor, Y., Tariq, T., & Zehra, S. S. (2019). Intrinsic bio-enhancer entities of *Fagonia cretica* for synthesis of silver nanoparticles involves anti-urease, anti-oxidant, and anti-tyrosinase activity. *Advances in Bioscience and Biotechnology*, *10*(12), 455-468. https://doi.org/10.4236/abb.2019.1012032
22. Mohanta, Y. K., Biswas, K., Panda, S. K., Bandyopadhyay, J., De, D., Jayabalan, R., & Mohanta, T. K. (2017). Phyto-assisted synthesis of bio-functionalized silver nanoparticles and their potential antioxidant, antimicrobial, and wound healing activities. *IET Nanobiotechnology*, *11*(8), 1027-1034. https://doi.org/10.1049/iet-nbt.2017.0017
23. Kiruba Daniel, S. G. C., Tharmaraj, V., Sironmani, T. A., & Pitchumani, K. (2010). Toxicity and immunological activity of silver nanoparticles. *Applied Clay Science*, *48*(4), 547-551. https://doi.org/10.1016/j.clay.2010.03.001
24. Zhang, K., Liu, X., Ravi, S. O. A. S., Ramachandran, A., Ibrahim, I. A., Nassir, A. M., & Yao, J. (2019). Synthesis of silver nanoparticles (AgNPs) from leaf extract of *Salvia miltiorrhiza* and its anticancer potential in human prostate cancer LNCaP cell lines.

Artificial Cells, Nanomedicine, and Biotechnology, 47(1), 2846-2854. https://doi.org/10.1080/21691401.2019.1638792

25. Banerjee, K., Choudhury, S. D. P., Ghosh, S., Baral, R., & Choudhuri, S. K. (2017). A novel approach of synthesizing and evaluating the anticancer potential of silver oxide nanoparticles in vitro. *Chemotherapy, 62*(5), 279-289. https://doi.org/10.1159/000453446
26. Thirumurugan, G., Shaheedha, S. M., & Dhanaraju, M. D. (2009). In vitro evaluation of antibacterial activity of silver nanoparticles synthesized by using *Phytophthora infestans*. *International Journal of ChemTech Research, 1*(3), 714-716.
27. McNamara, J. O. (2001). Drugs effective in the therapy of the epilepsies. New York: McGraw-Hill.
28. Bisht, A. S. (2015). Pharmacognostical and phytochemical evaluation of whole plant of *Asplenium dalhousiae* hook (fern). *World Journal of Pharmaceutical Research, 4*(9), 2186-2191.
29. Banerjee, K., Choudhury, S. D. P., Ghosh, S., Baral, R., & Choudhuri, S. K. (2017). A novel approach of synthesizing and evaluating the anticancer potential of silver oxide nanoparticles in vitro. *Chemotherapy, 62*(5), 279-289. https://doi.org/10.1159/000453446
30. Maher, S., Zamina, B., Riaz, M., Riaz, S., N. K., Imran, M., Fahmid, S., Ishtiaq, H., & Parveen, S. (2023). Green synthesis of *Withania coagulans* extract-mediated zinc oxide nanoparticles as photocatalysts and biological agents. *ACS Omega, 8*(49), 46715-46727. https://doi.org/10.1021/acsomega.3c05947
31. Hu, G., Liang, G., Zhang, W., Jin, W., Zhang, Y., Chen, Q., & Zhang, W. (2018). Silver nanoparticles with low cytotoxicity: controlled synthesis and surface modification with histidine. *Journal of Materials Science, 53*(7), 4768-4780. https://doi.org/10.1007/s10853-017-1940-6
32. Oboh, G., Akinyemi, A. J., & Ademiluyi, A. O. (2012). Inhibition of α-amylase and α-glucosidase activities by ethanolic extract of *Telfairia occidentalis* (fluted pumpkin) leaf. *Asian Pacific Journal of Tropical Biomedicine, 2*(9), 733–738. https://doi.org/10.1016/S2221-1691(12)60219-6
33. Nasir, G. A., Mohammed, A. K., & Samir, H. F. (2016). Biosynthesis and characterization of silver nanoparticles using olive leaves extract and sorbitol. *Iraqi Journal of Biotechnology, 15*(1), 22–32.
34. Devanesan, S., & AlSalhi, S. M. (2021). Green synthesis of silver nanoparticles using the flower extract of *Abelmoschus esculentus* for cytotoxicity and antimicrobial studies. *International Journal of Nanomedicine, 14*, 3343-3356. https://doi.org/10.2147/IJN.S307676
35. Ghani, S., Rafiee, B., Bahrami, S., Mokhtari, A., Aghamiri, S., & Yarian, F. (2022). Green synthesis of silver nanoparticles using the plant extracts of *Vitex agnus-castus L*: An eco-friendly approach to overcome antibiotic resistance. *International Journal of Preventive Medicine, 13*, 133. https://doi.org/10.4103/ijpvm.ijpvm14022
36. Mehta, B. K., Chhajlani, M., & Shrivastava, B. D. (2017). Green synthesis of silver nanoparticles and their characterization by XRD. *Frontiers of Physics and Plasma Science Journal of Physics, 836*(1), 012050. https://doi.org/10.1088/1742-6596/836/1/012050
37. Bewick, V., Cheek, L., & Ball, J. (2004). Statistics review 9: One-way analysis of variance. *Critical Care, 8*(2), 130-136. https://doi.org/10.1186/cc2836
38. Wickramaratne, M. N., Gunatilake, L. P., Anuradha, N. G. D., Godavillathanna, A. N., Perera, M. G. N., & Nicholas, I. (2015). Antioxidant activity and antibacterial activity of *Walidda antidysenterica*. *Journal of Pharmacognosy and Phytochemistry, 4*(2), 121-126.
39. Mittal, K., Tripathy, D., Choudhary, A., Aili, P. K., Chatterjee, A., & Singh, I. P. (2015). Bio-synthesis of silver nanoparticles using *Potentilla fulgens* Wall. ex Hook. and its therapeutic evaluation as anticancer and antimicrobial agent. *Materials Science and Engineering: C, 53*, 120-127. https://doi.org/

Figure

Click here to access/download;Figure;shafia XRD.jpg

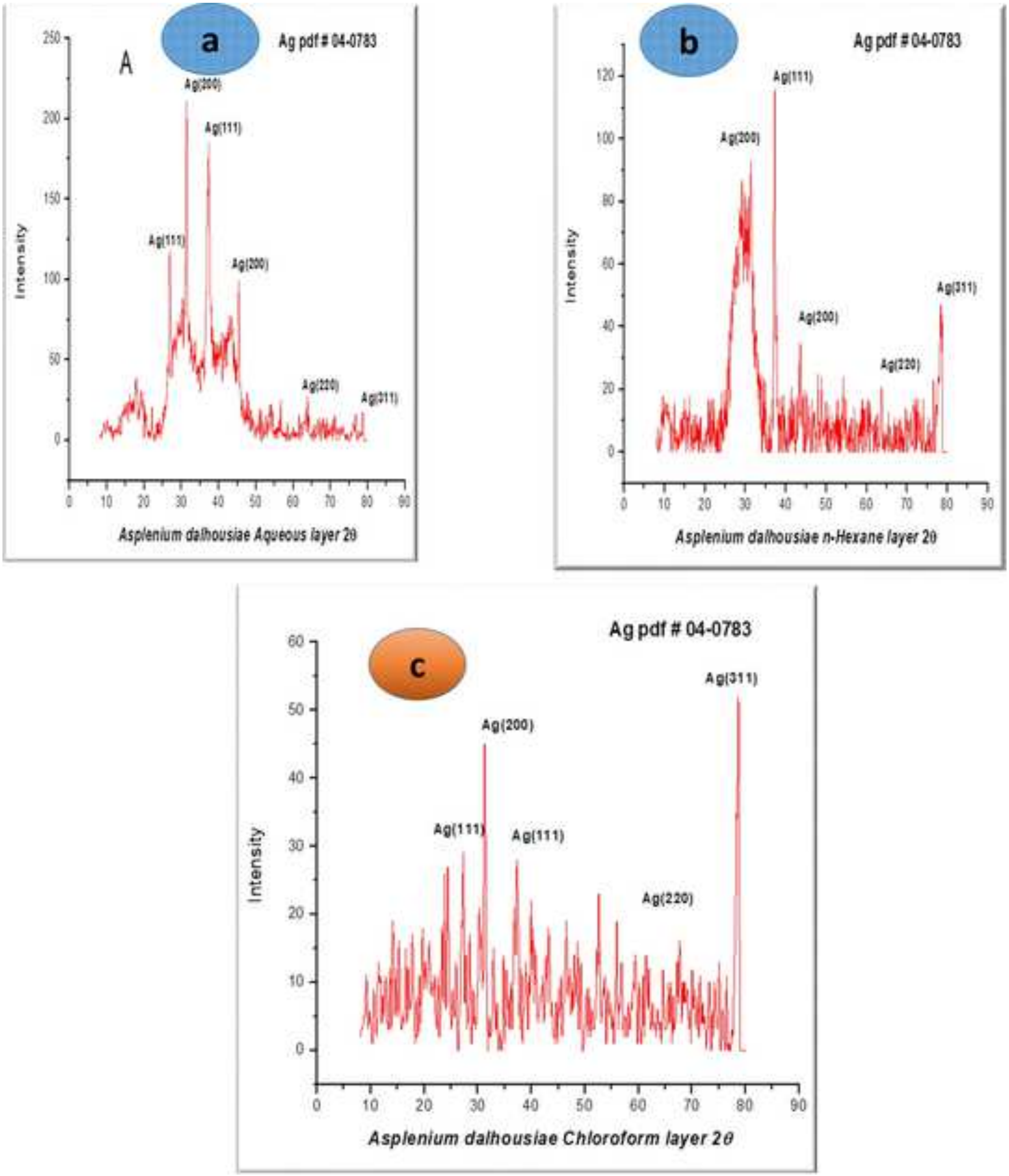

 Click here to access/download;Figure;graphical abstract.jpg

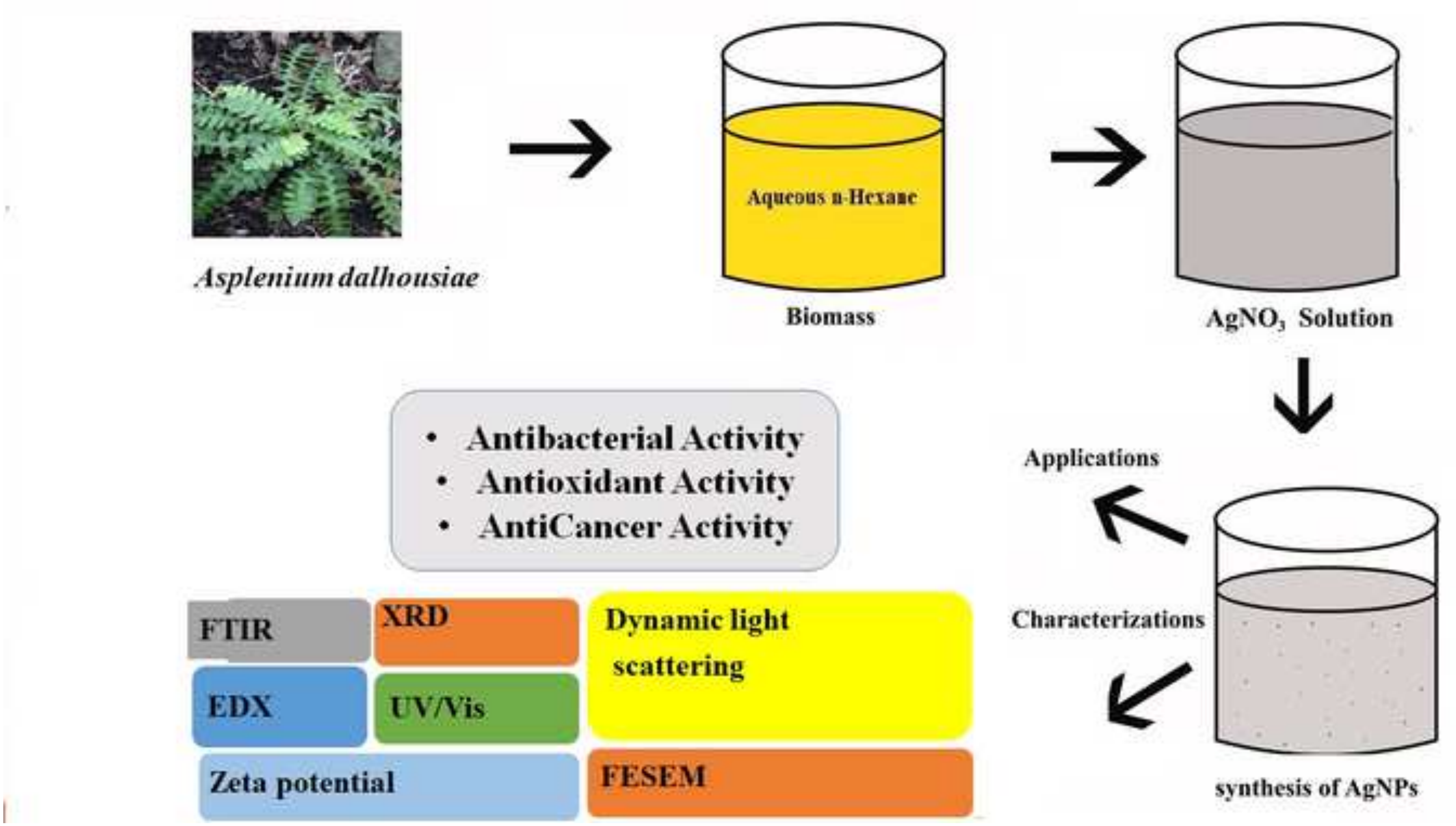

Figure
Click here to access/download;Figure;figures PLOS ONE.docx

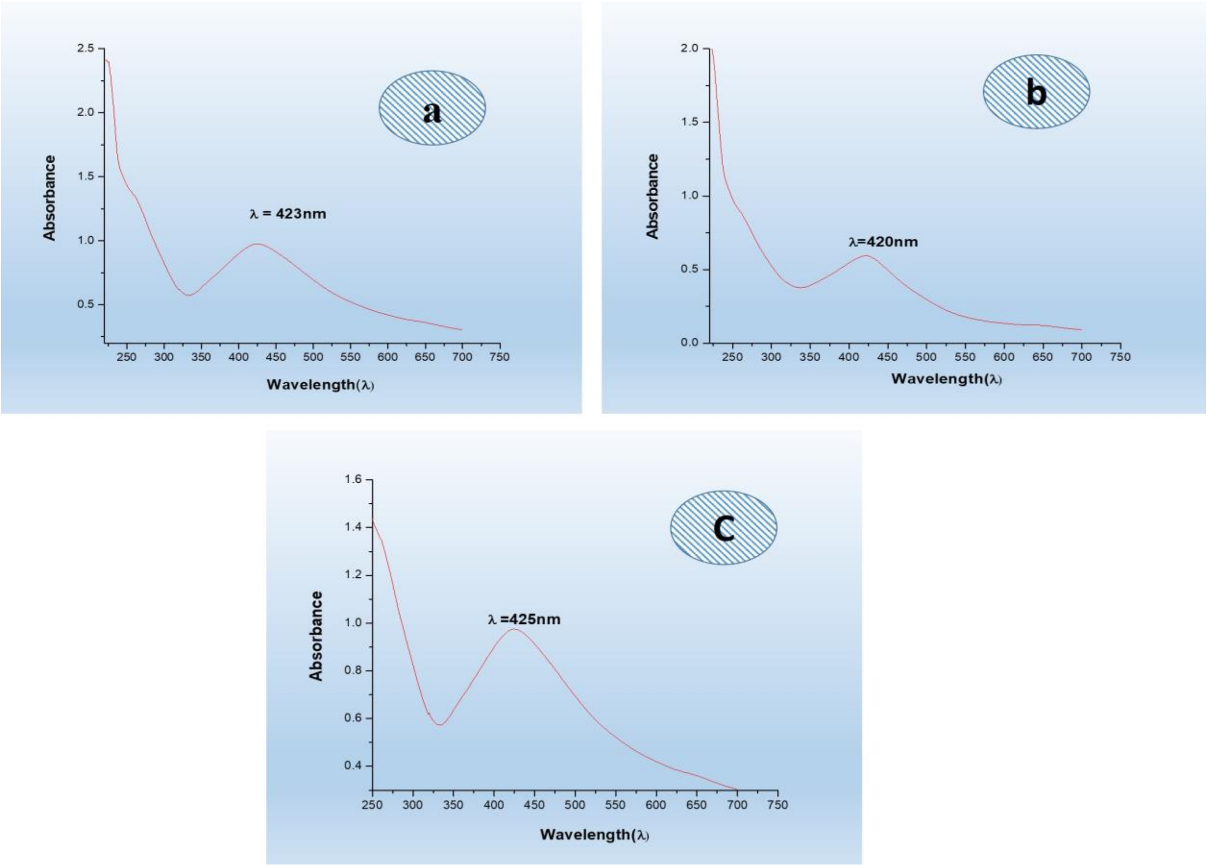

Fig 1. UV-Vis spectrograph of *Asplenium dalhousiae* in three selected layers.

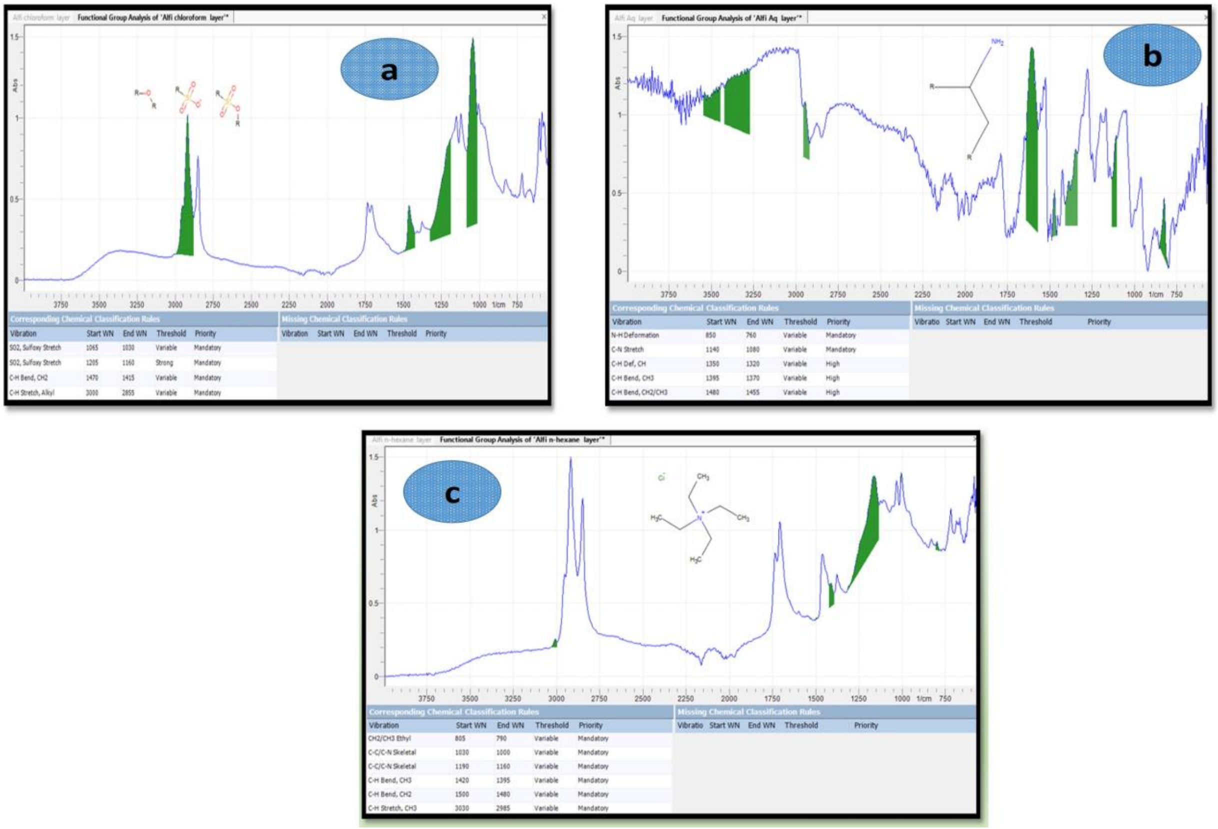

Fig 2: FTIR Spectrum of *Asplenium dalhousiae* in three selected layer. a) aqueous b) chloroform c) *n*-hexane extracts.

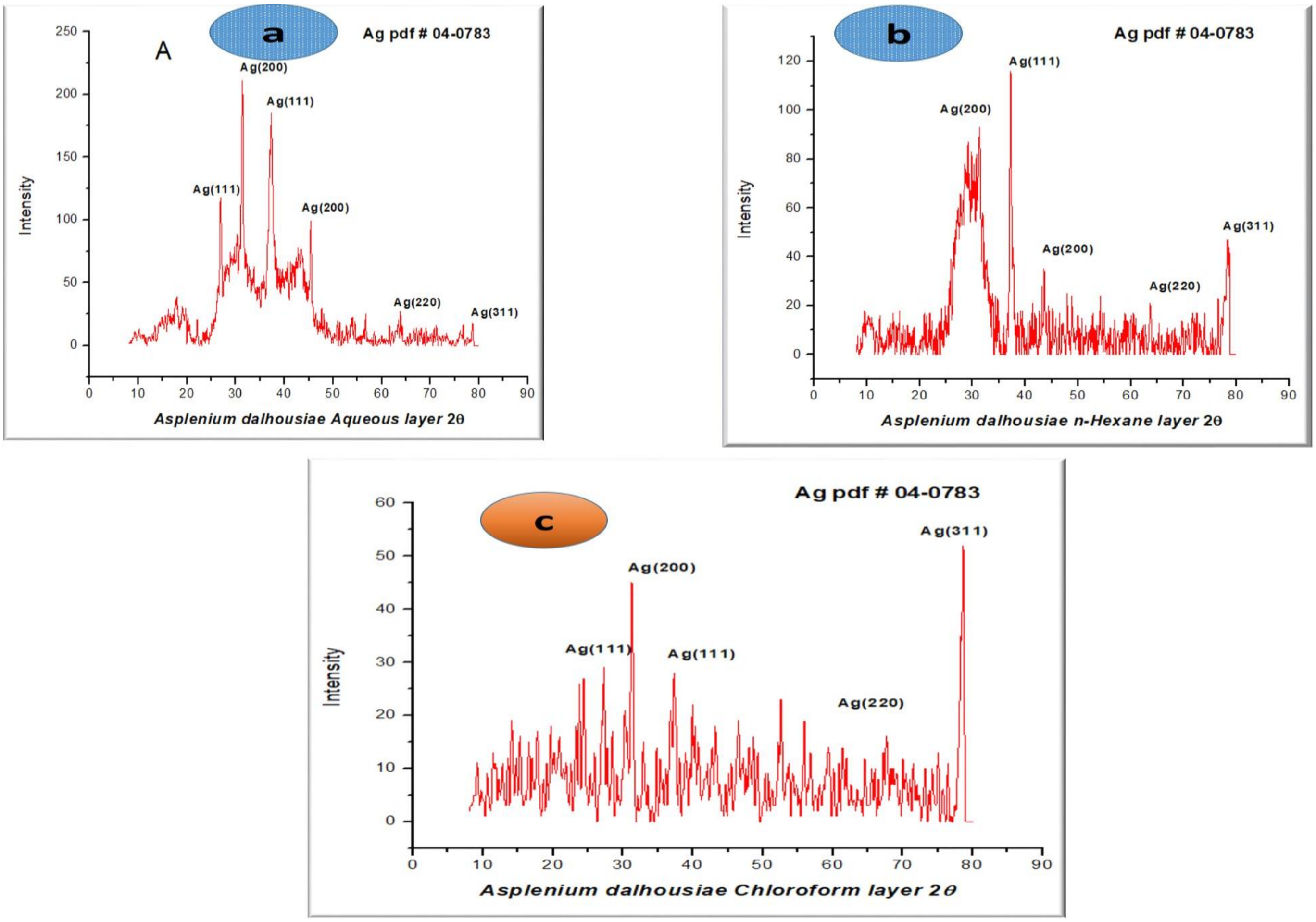

Fig 3. XRD result obtained from dried powdered of *Asplenium dalhousiae* plant extract, aqueous layer, b) *n*-Hexane layer, c) chloroform layer.

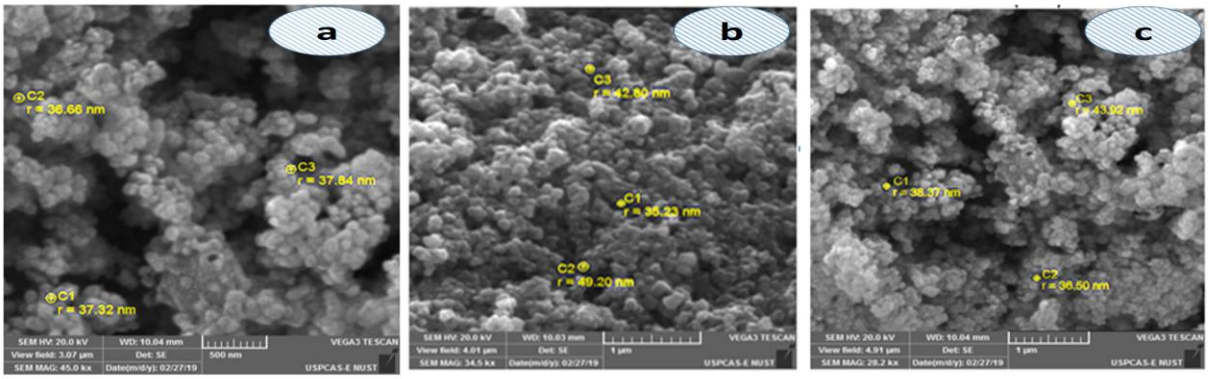

Fig 4. The SEM images of AgNps (*Asplenium dalhousiae*) plant extract, a) aqueous layer, b) *n*-Hexane layer, c) chloroform layer.

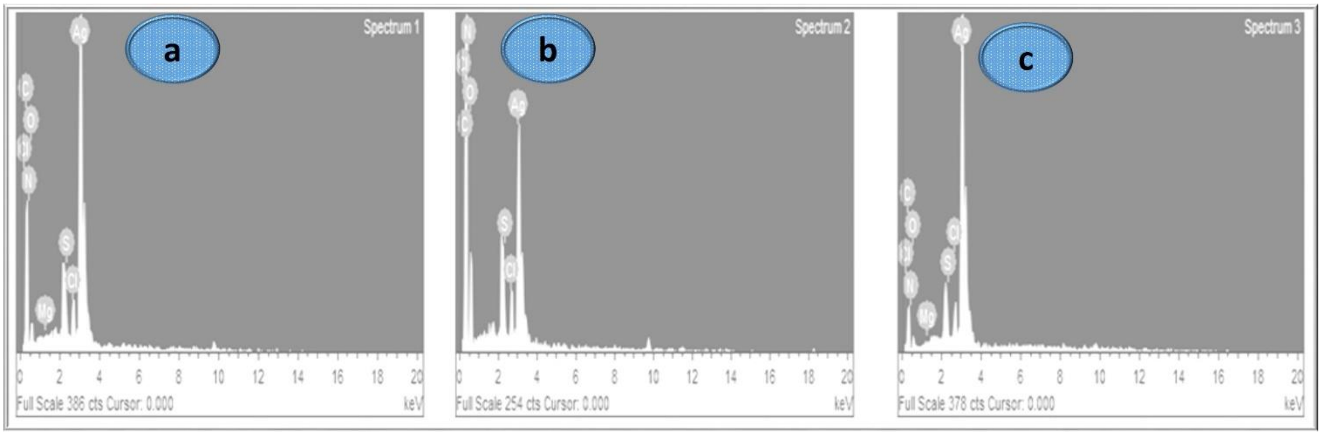

Fig 5. EDX of AgNps (A*splenium dalhousiae*) plant extract a) Aqueous layer, b) chloroform layer, c) *n*-hexane layer.

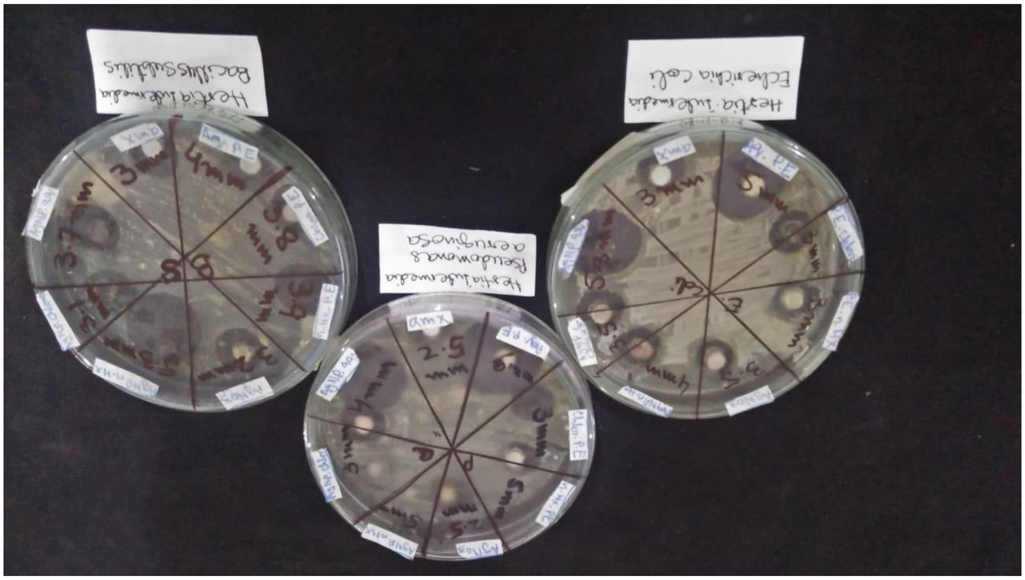

Fig 6: Zone of inhibition of Antibacterial Activity.

Figure Click here to access/download;Figure;table of Plos ONE.docx

Table 1: Antibacterial activity of silver nanoparticles and plant extracts against selected bacterial strains.

Group format	Doses µg/ml	*E. coli* (mm)		*B. subtilis* (mm)		*P. aeruginosa* (mm)	
**Extracts**	**Mean**	**Mean**	**SD**	**Mean**	**SD**	**Mean**	**SD**
$AgNO_3$	30.	18.0	1.2	17.5	1.6	18.0	1.2
AgNP of $CHCl_3$ extract	30.	19.0	1.2	18.0	0.6	18.0	1.4
AgNP Aqueous extract	30.	19.5	1.4	17.5	1.6	17.0	1.1
AgNP *n*-Hexane layer	30.	20.0	1.8	19.0	1.2	19.5	1.4
Plant extract of $CHCl_3$ extract	30.	19.5	1	17.0	1.2	18.0	1.2
Plant extract of Aqueous layer	30.	16.0	1.2	15.0	1.6	16.5	1.2
Plant extract of *n*-Hexane layer	30.	17.0	1.2	15.0	0.6	15.0	1.2
Standard (Amoxycillin)	30.	23.0	1.2	22.0	1.2	23.0	1.6

Table 2: DPPH Radical Scavenging Capacity of Ascorbic Acid and (AgNPs)

Concentrations	Ascorbic Acid (Mean ± SD)	AgNPs Mean ± SD
10	45.69±0.6	37.19±1.2
20	52.54±1.4	48.45±1.6
40	64.27±1.2	58.06±1.8
60	90.77±0.8	85.12±1.6
80	110.06±1.6	96.56±1.2
100	108.77±1.2	104.38±1.8

Table 3: DPPH radical scavenging capacities of ascorbic acid and AgNPs.

S. No	Concentration (µg/ml)	% Cell Viability		
		**AgNPs**	**Ascorbic Acid**	**SD**
1	10	37.19	45.69	1.2
2	20	48.45	52.54	1.6
3	40	58.06	64.27	1.8
4	60	85.12	90.77	1.6
5	80	96.56	110.06	1.2
6	100	104.38	118.77	1.8
IC50 (µg/ml)		23.64374724	17.78071696	56.795

Table 4: Inhibition of *in-vitro* α-amylase enzyme assay.

Groups	Concentration (µg/ml)	% Cell Viability		
		Asplenium dalhousiae	**Ag NPs**	**Standard**
Group I	100	15.25±1.01	18.58±1.30	23.45±1.25
Group II	200	30.55±1.50	38.5±2.08	42.61±2.45
Group III	300	44.16± 2.40	57.7±3.09	65.74±3.06
Group IV	400	65.05±3.01	71.44±3.80	79.31±3.70
Group V	500	78.65±4.02	85.4±4.09	90.84±4.56
IC50 (µg/ml)		320.26	274.04	239.40

Table 5: The anticancer activity of different samples against HCT116 and A2780 cancer cell line

**Sample I.D.** AgNPs	**Standard Conc. (µg/ml)**	**Inhibition against HCT116**		**Inhibition against A2780**	
		**Cell Viability (%)**	**IC_{50} ± SD**	**Cell Viability (%)**	**IC_{50} ± SD**
CF	30	25.5± 1.2	Active	23.2	Active
HF	30	27.7±0.6	Active	25.3	Active
AF	30	19.8±1.4	Active	18.5	Active

Table 6. *Asplenium dalhousiae* AgNPs inhibition against HCT116 and A2780.

**I.D No.**	**IC_{50} in HCT116 (µg/ml)**	**Sem**	**Replicate**	**IC_{50} in A2780 (µg/ml)**	**Sem**	**Replicate**
AgNP's aqueous	21.11	0.93	**2**	15.76	7.75	**2**
AgNP's, *n-h*exane,	45.98	29.56	**2**	9.11	2.55	**2**
AgNP's $CHCl_3$	605.2	258.3	**2**	247.66	188.64	**2**
Plant methanolic Extract	603.25	45.35	2	34.97	27.38	2
**Result**	Active					

Click here to access/download
Supporting Information
anticancer 1.jpg

Click here to access/download
Supporting Information
anticancer 2.jpg

Click here to access/download
Supporting Information
bac,dppha,dia.jpg

Click here to access/download
Supporting Information
Shafia IR.jpg

Click here to access/download
Supporting Information
Shafia UV.jpg

Click here to access/download
Supporting Information
shafia FESEM.jpg

Click here to access/download
Supporting Information
Shafia edx.jpg

Click here to access/download
Supporting Information
graphical abstract.docx
