# Supplementary material for: Biosynthesis and characterization of silver nanoparticles from Asplenium dalhousiae and their potential biological properties
Source: PLoS One. 2025 Jun 30;20(6):e0325533. doi: 10.1371/journal.pone.0325533 (PMC12208411; doi:10.1371/journal.pone.0325533)
Supplement: Attachment — Submitted filename: PONE-D-24-42400-Reviewer Comments-V1.docx [file pone.0325533.s008.docx]

Manuscript ID: PONE-D-24-42400

Biosynthesis and Characterization of Silver Nanoparticles from Asplenium dalhousiae and their Potential Biological Properties

This study explores the feasibility of green synthesis of AgNPs using plant extracts, with antibacterial, antioxidant, and cytotoxic activities from Asplenium dalhousiae plants. Although the study emphasizes the potential biological properties of Asplenium dalhousiae, but it does not adequately justify its selection over other well-established method for synthesis of AgNPs using plant extracts. Significant efforts are required to differentiate this work from the current published literature and to claim the novelty. In addition, two kind of manuscript file submitted in one file the first one and the second one which is the better version and the following correction are suggested for the second one.

In its present form, the manuscript is not recommended for publication unless the following issues are addressed:

1. The English language and grammar in the whole manuscript needs further improvement, particularly in sections such as Introduction, Materials and Methods.

2. The statements such as “paving the way for improved treatment outcomes in patients with ovarian cancer” mentioned in the end of the Abstract are speculative without preclinical or clinical validation. Cytotoxicity is an in-vitro property that does not equate with the therapeutic efficacy for in-vivo and should be compared with literature to substantiate differences.

3. Figures are poorly presented and should be arranged into collages for better readability and interpretation.

4. The study lacks mechanistic insights, such as identifying specific compounds in Asplenium dalhousiae responsible for nanoparticle synthesis and their specific role in reduction and stabilization.

5. The α-amylase inhibition and cytotoxic mechanisms are not explained well by authors.

6. The authors claim in the introduction section that the reported work is the "first comprehensive investigation" of Asplenium dalhousiae which is unsupported with the previously published and related research. Comparison with other studies on plant-mediated AgNP synthesis is required to highlight the novelty.

7. Characterization methods such as UV-Vis, FTIR, XRD, and SEM are discussed generally, without any specific experimental optimization or justifications, that could be associated with the changes made before and after synthesis in the results obtained through these characterizations.

8. Statistical analysis is missing and biological assay results should include error bars, p-values, and measures of significance.

9. The bio-reduction process needs deeper exploration, linking phytochemicals and functional groups identified in FTIR to biological activities.

10. Implications like scalability, toxicity, and economic feasibility of Asplenium dalhousiae-based AgNPs are not well addressed by the authors.

11. Redundancy is evident, with repeated discussions on green synthesis of AgNPs’ and its biomedical roles without adding new insights.

12. Oversimplified statements such as “Nanoparticles are far more dangerous to cancerous cells than bulk materials,” these statements show lack of explanation with supporting references.

13. The antioxidant assay concentration range (10–100 µg/ml) is vague and lacks justification linked to standard protocols with particular reference.

14. Broad peaks in XRD patterns require consideration of factors like crystallanity and particle size distribution with particular strain during interpretation.

15. Missing details, such as solvent partition criteria and centrifugation rationale, hindered reproducibility.

16. The reported particle size range in SEM is of 1–100 nm is excessively broad and should include specific details like average size, standard deviation, and size distribution.
