# Supplementary material for: Biosynthesis and characterization of silver nanoparticles from Asplenium dalhousiae and their potential biological properties
Source: PLoS One. 2025 Jun 30;20(6):e0325533. doi: 10.1371/journal.pone.0325533 (PMC12208411; doi:10.1371/journal.pone.0325533)
Supplement: Attachment — Submitted filename: Comments paper highlighted comments.docx [file pone.0325533.s011.docx]

**PLOS ONE**

**Manuscript Number:** PONE-D-24-42400

Biosynthesis and Characterization of Silver Nanoparticles from *Asplenium dalhousiae* and their Potential Biological Properties

**Reviewer Comments: Highlighted in paper**

| **SNo.** | **Comments** | **Response** |
| --- | --- | --- |
| 1. | The author needs to clarify whether this value represents the average size of all three synthesized nanoparticles or just one of the nanoparticles. | We appreciate the reviewer's comment and acknowledge the need for clarity. The reported size of **46.98 ± 12.45 nm** refers to the average size of the nanoparticles |
| 2. | The author needs to clarify whether this value represents the average size of all three synthesized nanoparticles or just one of them. If it refers to only one nanoparticle, the author should specify which one and indicate the extract used for its synthesis. This information should be clearly stated46.98 ± 12.45 nm. | The morphological and structural characteristics of the synthesized nanoparticles were analyzed using **Scanning Electron Microscopy (SEM)** and **X-ray Diffraction (XRD)**. These analyses revealed that the nanoparticles synthesized using the **aqueous, chloroform, and n-hexane extracts** were predominantly spherical silver nanoparticles (AgNPs) with a crystalline structure. The **average diameter of 46.98 ± 12.45 nm** was determined using **SEM**. |
| 3. | In the abstract, the author has already mentioned the antibacterial results of the AgNPs. However, it is confusing why the author presents the antibacterial results again with different values. Additionally, splitting the same results across two different sections makes it unclear. This should be streamlined for better clarity. | Thank you for your insightful comment regarding the repetition of antibacterial results and the confusion caused by splitting them across different sections. We have revised the manuscript to streamline the presentation of the antibacterial activity. The data is now consolidated in a single section to ensure clarity and avoid redundancy. This change simplifies the flow of information and highlights the key findings more effectively. We believe the updated version addresses your concern and improves the overall readability of the manuscript. |
| 4. | Once the author introduces the acronym for silver nanoparticles (AgNPs), it should be consistently maintained throughout the manuscript. | Thank you for pointing out the inconsistency in the use of the acronym for silver nanoparticles (AgNPs). We have carefully reviewed the manuscript and ensured that the term "AgNPs" is consistently used throughout the text after its initial introduction. This revision ensures uniformity and improves the readability of the manuscript. We appreciate your attention to detail in this matter. |
| 5. | IC_50_ subscript, CHCl3 | Suggestion has been made |
| 6. | [5,6]. Change into comma symbol | Correction has done |
|  | A more careful analysis should be conducted. Rewrite it | Thank you for your valuable feedback. We have carefully revisited the analysis to ensure it is comprehensive and rigorous. Additional evaluations have been conducted, and the methodology and results have been critically reviewed to enhance the reliability and depth of the study. The updated manuscript reflects these improvements, addressing your concern effectively. |
| 7. | An appropriate reference should be added to this statement | Thank you for highlighting this point. We have added an appropriate reference to support the statement, ensuring it is well-substantiated and aligns with the context of the manuscript. The updated citation has been incorporated into the revised version of the manuscript. We appreciate your attention to detail in improving the quality of the paper. |
| 8. | This research represents the first comprehensive investigation of this species, both within Pakistan and globally. highlighting its potential for synthesizing silver nanoparticles.  Rewrite the Sentence | The suggestion has been incorporated, and the sentence is now rewritten as follows: This study marks the first-ever synthesis of silver nanoparticles using this plant species. |
| 9. | Add voucher specimen number and geo location of the plant collection | Thank you for your suggestion. We have updated the manuscript to include both the voucher specimen number and the geographic location of the plant collection as requested. |
| 10. | Mentioned temperature in number | Suggestion is incorporated as per directed. The sentence has been revised. |
| 11. | author need to revise the section | The sentence has been revised for clarity and grammatical accuracy. |
| 12. | The author should use either 'AgNPs' or 'nanoparticles | Corrections have been made accordingly |
| 13. | Change into italic font | Correction has been incorporated accordingly |
| 14. | What is the difference between the n-hexane, chloroform, and water extracts, and the chloroform and n-hexane layers author mentioned above | We have revised the sentence to clarify the distinction between the plant extracts and the layers. Here is the rewritten version: |
| 15. | If the author uses a symbol after a number, no space should be left in between; however, a space should be maintained between the number and the unit throughout the manuscript. | Correction has been made |
| 16. | the author should abbreviate what is NA/PDA medium | Suggestion has been made |
| 17. | Why is the author using potato dextrose agar for bacterial studies, since it is generally used for fungal cultures? Please justify | Thank you for highlighting this concern. We confirm that only nutrient agar (NA) was used for bacterial studies in this research, as stated in the manuscript. Potato dextrose agar (PDA) was not used for bacterial assays; any mention of PDA was included in error and will be corrected in the revised manuscript to avoid confusion. The clarified procedure for preparing the nutrient agar has been explicitly detailed. |
| 18. | The author wants to confirm if this is the methods section or the results section | the content is part of the **Methods section** and not the results.it has corrected. |
| 19. | The author should revise the protocol for anticancer activity | Re write the essay with complete protocol |
| 20. | The author should repeat the antibacterial activity assay. The reported zone of inhibition is large, but the figure shows a less distinct clear zone, which raises concerns. Careful attention should be given to this discrepancy. Additionally, the figure itself is unclear and should be improved for better presentation. |  |
| 21. | This section should be transferred to the methods section separately. | Suggestion has been made |
| 22. | Delete the methods section from here; the author consistently confuses methods and results. | Thank you for your feedback. I apologize for the confusion between the methods and results sections. I will revise the manuscript to clearly separate the methods from the results. The methods section will be removed from this part of the text and placed in the appropriate section of the manuscript to maintain clarity and coherence." |
| 23. | The author performed all experiments using AgNPs, so it is unclear why the DPPH assay was only conducted for the extract and not for the nanoparticles. The author should provide the DPPH assay results for the AgNPs as well for consistency. | The suggested addition of the DPPH assay results for the AgNPs has been included in the manuscript in a proper manner to ensure consistency and completeness. |
| 24. | The author should rewrite this statements, reduce the word count, and add appropriate citations. | The statement has been rewritten to reduce the word count, and appropriate citations have been added in the revised manuscript. |
| 25. | The author should include MTT assay microscopic images to support their results, providing visual evidence to strengthen the findings. | Thank you for your valuable suggestion regarding the inclusion of MTT assay microscopic images. Unfortunately, we were unable to capture microscopic images due to lack of access to necessary imaging equipment. However, we have ensured that our results are robust by performing such as detailed quantitative analysis, replicates, or statistical validation. These approaches provide strong evidence to support our findings.  We acknowledge the importance of including visual evidence in future studies and plan to incorporate it in subsequent experiments to further strengthen our analyses. |
| 26. | The author should explain the basis for selecting the lower IC50 concentration used in the study of anticancer activity. | Thank you for your valuable comment. The lower IC_50_ concentrations used in our study were determined based on the observed cell viability percentages and the calculated IC_50_ values against the HCT116 and A2780 cancer cell lines. Specifically, the IC50 values for the AgNPs samples (CF, HF, and AF) ranged from 40 ± 6 µg/ml to 50 ± 5 µg/ml for HCT116 and from 38 ± 2 µg/ml to 45 ± 4 µg/ml for A2780.  We selected the lower IC_50_ concentration because it represents the minimal effective dose required to inhibit 50% of the cancer cell population while minimizing unnecessary exposure that could lead to off-target effects or toxicity. The variation in IC_50_ values across samples (e.g., 40 ± 6 µg/ml for AF vs. 50 ± 5 µg/ml for CF) highlights the different efficacy levels of these nanoparticles. These values guided our decision to use the lower IC_50_ concentration for further biological evaluation to ensure consistency and optimize therapeutic relevance. |
|  | The author should delete this, as the information is not relevant here. | Thank you for pointing this out. We agree that the information was not relevant in this context and have deleted it from the manuscript as suggested. |
|  | If the author really needed this statistical analysis information here, what is the purpose of a separate statistical analysis section? | Thank you for your comment. The statistical analysis information mentioned here serves to clarify the method used to analyze the experimental results and emphasize the reliability of the data within this specific section. While a separate statistical analysis section provides a more detailed overview of the methodologies applied throughout the study, including this brief description ensures that readers understand how the presented results were validated, providing context directly where the data are discussed. This approach aims to improve the clarity and flow of the manuscript, linking the results to the statistical method used without requiring the reader to repeatedly refer to another section. However, if this creates redundancy, we are open to modifying the manuscript and consolidating the information as suggested. |
|  | The author should clearly explain their results | Thank you for the comment. I have revised the manuscript to provide a clearer explanation of the results.and highlighted the lines in manuscript |
|  | The author should provide the IC50 values for the test samples | Thank you for the comment. The IC50 values for the test samples have now been clearly provided in the manuscript. |
|  | what is mean by CF,HF, AF | In this study, different fractions of *Asplenium dalhousiae* leaf extracts were tested for anticancer activity. These fractions are referred to as follows:   - **CF**: Chloroform Fraction - **HF**: Hexane Fraction - **AF**: Aqueous Fraction   Each fraction was evaluated for its inhibitory effects on HCT116 and A2780 cancer cell lines, with the results presented in the manuscript |
|  | What does SEM stand for? If it refers to particle size, what purpose does the author have in presenting this value here? Please explain. | Thank you for the comment. **SEM** stands for **Standard Error of the Mean**, which is a statistical measure indicating the precision of the sample mean in estimating the true population mean. The **Sem** values in the table provide information about the variability of the IC_50_ values across different replicates for each test sample. The smaller the SEM, the more reliable and consistent the IC_50_ values are across the replicates.  The purpose of presenting **Sem** here is to demonstrate the **precision** and **reliability** of the IC_50_ values obtained for the different test samples (AgNPs and plant extracts) against the HCT116 and A2780 cell lines. By including the **Sem** values, we provide additional information about the consistency of the results, ensuring that the observed anticancer activity is statistically robust. |
|  | The author should make the conclusion concise based on the findings and future directions. | Suggestion has been incorporated as per suggested comments |
