# Supplementary material for: Biosynthesis and characterization of silver nanoparticles from Asplenium dalhousiae and their potential biological properties
Source: PLoS One. 2025 Jun 30;20(6):e0325533. doi: 10.1371/journal.pone.0325533 (PMC12208411; doi:10.1371/journal.pone.0325533)
Supplement: Attachment — Submitted filename: response to reviewer.docx [file pone.0325533.s012.docx]

**PONE-D-24-42400**

**Biosynthesis and Characterization of Silver Nanoparticles from *Asplenium dalhousiae* and their Potential Biological Properties**

**PLOS ONE**

**Reviewer #1:**

**The effects of the AgNPs using *Asplenium dalhousiae* on their antibacterial, antioxidant, α-amylase inhibitory and cytotoxic activities.**

**General comments,**

|  | **Comments** | **Response** |
| --- | --- | --- |
| 1. | In the abstract, the author initially mentioned that antibacterial activity was tested against Bacillus subtilis and Escherichia coli. However, this information does not match the results section. The author should ensure consistency and correct this based on the original experiment. | Thank you for pointing out the inconsistency. We have reviewed the manuscript and corrected the abstract to ensure it accurately reflects the experimental details presented in the results section. The antibacterial activity was evaluated against *Escherichia coli*, *Bacillus subtilis*, and *Pseudomonas aeruginosa*, and the abstract has now been revised accordingly to include all three tested bacterial strains. |
| 2. | The author should clarify whether plant extracts (aqueous, chloroform, and n-hexane) were obtained through natural dissolution and provide a more detailed explanation of the extraction process using different solvents. | Thank you for your valuable suggestion. We have revised the manuscript to clarify the extraction procedure. The plant extracts (aqueous, chloroform, and n-hexane) were obtained through successive solvent extraction using a Soxhlet apparatus. Initially, the dried and powdered plant material was extracted with n-hexane, followed by chloroform and finally with deionized water to ensure polarity based fractionation. Each solvent was used for an appropriate duration to maximize yield, and the resulting extracts were concentrated under reduced pressure using a rotary evaporator. The detailed procedure has now been added to the Materials and Methods section. |
| 3. | The UV-Vis spectroscopy section should be rewritten separately, with some portions moved to the Results and Discussion section. | We have now separated the UV-Visible spectroscopy data and analysis into the Results and Discussion section for improved clarity and coherence. The synthesis description is now focused solely on the visual and procedural aspects, while spectral details and comparisons are discussed in the dedicated analysis section. References to previous literature have been retained and appropriately cited for comparison. |
| 4. | Additionally, the author should provide details of the instruments used, including the make and manufacturer, for all technical analyses. | We have now included the specific details of all instruments used in the characterization of the synthesized silver nanoparticles. The make, model, and manufacturer for each analytical technique (e.g., UV-Vis spectrophotometry, FTIR, SEM, XRD) have been clearly stated in the revised **Materials and Methods** section to improve clarity, transparency, and reproducibility. |
| 5. | The manuscript contains several typographical errors (e.g., punctuation mistakes in periods, commas, superscripts, and subscripts) and grammatical issues. These should be corrected, and the author should ensure a smooth flow of sentences. | We have carefully proofread the entire manuscript to correct all typographical and grammatical errors. We have also revised several sentences to improve clarity and ensure a smooth and logical flow throughout the text. The revised manuscript has been edited accordingly. |
| 6. | Preparation of *Asplenium dalhousiae* Extract and AgNPs: The term "AgNPs water" is unclear and should be clarified. | Thank you for your observation. To improve clarity and scientific precision, we have replaced the term “AgNPs water” with “aqueous colloidal solution of silver nanoparticles” in the revised manuscript. This change ensures accurate communication of the nature of the synthesized nanoparticles and aligns with standard terminology used in nanomaterials research. |
| 7. | The methodology for the preparation of Asplenium dalhousiae extract and AgNPs synthesis is confusing and should be revised for better clarity. | Thank you for your valuable suggestion. We have carefully revised the methodology section to improve clarity and ensure a logical flow of information. The updated paragraph describing the preparation of Asplenium dalhousiae extract and silver nanoparticle (AgNPs) synthesis is now more structured and scientifically precise. The revised text has been **highlighted** in the manuscript for easy identification. |
| 8. | The Statistical Analysis section should be placed at the end of the Methods section. | As advised, we have relocated the Statistical Analysis section to the end of the Materials and Methods section in the revised manuscript. |
| 9. | Some words in the manuscript are bolded without a clear reason. The author should clarify if there is a specific purpose for this formatting. | We have now removed the unnecessary bold formatting to ensure consistency and maintain a professional presentation throughout the manuscript. |
| 10. | During AgNP synthesis, the observed color change is inconsistent. Some sections describe a transition from colorless to reddish-brown, while others mention yellow to orange-brown. The author should carefully revise the manuscript to ensure consistency in experimental results and methods. | We have revised the manuscript to ensure consistency in reporting the color change. The correct transition has now been clearly stated as **yellow to orange-brown** throughout the manuscript to align with the experimental observations. |
| 11. | Figure 3 (FTIR Spectrum of Asplenium dalhousiae Plant Extract): The legend describes FTIR analysis of aqueous, chloroform, and n-hexane extracts. If the results refer to AgNPs rather than plant extracts, the legend should be revised accordingly. | We acknowledge the oversight in the original figure legend. The FTIR spectra presented in Figure 3 correspond to the silver nanoparticles (AgNPs) synthesized using Asplenium dalhousiae extracts, not the crude plant extracts themselves. We have revised the figure legend accordingly to accurately reflect the content: |
| 12. | Figure 4 (XRD Analysis of AgNPs): The author should maintain consistency in terminology when referring to plant extracts or layers. | The Correction has been incorporated throughout the manuscript |
| 13. | The figure and legend should be revised for uniformity. | We have made all the necessary revisions. |
| 14. | Table 1 (Antibacterial Activity of Silver Nanoparticles and Plant Extracts): The mean values and dose figures should be formatted uniformly. | I have ensured that the formatting of the **mean values** and **dose figures** in **Table 1** is consistent throughout. Specifically: |
| 15. | The author should verify whether Figure 7 represents the antidiabetic test results. | Thank you for the feedback. We have carefully reviewed Figure 7, and all activities (DPPH, antibacterial, and anti-diabetic) are clearly labeled with distinct colors, markers, and legends. The anti-diabetic test results are specifically marked and represented with appropriate axes and scale, ensuring clarity and easy differentiation from other activities. We have ensured that the figure accurately reflects the data and is clear for readers to interpret. |
| 16. | All figure legends should be revised, as they do not accurately describe the graphical data. The author is requested to use appropriate legends for each test result. | Thank you for your valuable feedback. We have revised all figure legends to ensure they accurately describe the graphical data. Each legend has been updated to clearly reflect the specific test results, and we have used appropriate terminology to describe the DPPH, antibacterial, and anti-diabetic activity data. We believe the revised legends now provide a clearer and more precise description of the experimental results. |
