# Supplementary material for: Biosynthesis and characterization of silver nanoparticles from Asplenium dalhousiae and their potential biological properties
Source: PLoS One. 2025 Jun 30;20(6):e0325533. doi: 10.1371/journal.pone.0325533 (PMC12208411; doi:10.1371/journal.pone.0325533)
Supplement: Peer review history

# Peer review: Decision Letter 0

**DOI:** 10.1371/journal.pone.0325533.r001

**Authors:** Chinnaperumal Kamaraj

Dear Dr. maher,

Thank you for submitting your manuscript to PLOS ONE. After careful consideration, we feel that it has merit but does not fully meet PLOS ONE’s publication criteria as it currently stands. Therefore, we invite you to submit a revised version of the manuscript that addresses the points raised during the review process.

==============================

I recommend that the authors make major revisions to address the comments raised by reviewers. The revised manuscript should be resubmitted for further evaluation. Please include a detailed response letter outlining how each of these comments has been addressed, along with any changes made to the manuscript.

Thank you for your submission.

==============================

Please submit your revised manuscript by Jan 12 2025 11:59PM. If you will need more time than this to complete your revisions, please reply to this message or contact the journal office at plosone@plos.org . When you're ready to submit your revision, log on to https://www.editorialmanager.com/pone/ and select the 'Submissions Needing Revision' folder to locate your manuscript file.

- A rebuttal letter that responds to each point raised by the academic editor and reviewer(s). You should upload this letter as a separate file labeled 'Response to Reviewers'.
- A marked-up copy of your manuscript that highlights changes made to the original version. You should upload this as a separate file labeled 'Revised Manuscript with Track Changes'.
- An unmarked version of your revised paper without tracked changes. You should upload this as a separate file labeled 'Manuscript'.

If you would like to make changes to your financial disclosure, please include your updated statement in your cover letter. Guidelines for resubmitting your figure files are available below the reviewer comments at the end of this letter.

If applicable, we recommend that you deposit your laboratory protocols in protocols.io to enhance the reproducibility of your results. Protocols.io assigns your protocol its own identifier (DOI) so that it can be cited independently in the future. For instructions see: https://journals.plos.org/plosone/s/submission-guidelines#loc-laboratory-protocols . Additionally, PLOS ONE offers an option for publishing peer-reviewed Lab Protocol articles, which describe protocols hosted on protocols.io. Read more information on sharing protocols at https://plos.org/protocols?utm_medium=editorial-email&utm_source=authorletters&utm_campaign=protocols .

We look forward to receiving your revised manuscript.

Kind regards,

Chinnaperumal Kamaraj, Ph.D

Academic Editor

PLOS ONE

Journal Requirements:

When submitting your revision, we need you to address these additional requirements.

1. Please ensure that your manuscript meets PLOS ONE's style requirements, including those for file naming. The PLOS ONE style templates can be found at https://journals.plos.org/plosone/s/file?id=wjVg/PLOSOne_formatting_sample_main_body.pdf and https://journals.plos.org/plosone/s/file?id=ba62/PLOSOne_formatting_sample_title_authors_affiliations.pdf

2. In your Methods section, please provide additional information regarding the permits you obtained for the work. Please ensure you have included the full name of the authority that approved the field site access and, if no permits were required, a brief statement explaining why.

3. We note that your Data Availability Statement is currently as follows: [All relevant data are within the manuscript and its Supporting Information files. If additional details or access are needed, please contact me directly.] Please confirm at this time whether or not your submission contains all raw data required to replicate the results of your study. Authors must share the “minimal data set” for their submission. PLOS defines the minimal data set to consist of the data required to replicate all study findings reported in the article, as well as related metadata and methods (https://journals.plos.org/plosone/s/data-availability#loc-minimal-data-set-definition). For example, authors should submit the following data: - The values behind the means, standard deviations and other measures reported; - The values used to build graphs; - The points extracted from images for analysis. Authors do not need to submit their entire data set if only a portion of the data was used in the reported study. If your submission does not contain these data, please either upload them as Supporting Information files or deposit them to a stable, public repository and provide us with the relevant URLs, DOIs, or accession numbers. For a list of recommended repositories, please see https://journals.plos.org/plosone/s/recommended-repositories. If there are ethical or legal restrictions on sharing a de-identified data set, please explain them in detail (e.g., data contain potentially sensitive information, data are owned by a third-party organization, etc.) and who has imposed them (e.g., an ethics committee). Please also provide contact information for a data access committee, ethics committee, or other institutional body to which data requests may be sent. If data are owned by a third party, please indicate how others may request data access.

4. Please ensure that you refer to Figures 1-8 in your text as, if accepted, production will need this reference to link the reader to the figure.

5. We note you have included a table to which you do not refer in the text of your manuscript. Please ensure that you refer to Tables1-6 in your text; if accepted, production will need this reference to link the reader to the Table.

Additional Editor Comments:

I recommend that the authors make major revisions to address the comments raised by reviewers. The revised manuscript should be resubmitted for further evaluation. Please include a detailed response letter outlining how each of these comments has been addressed, along with any changes made to the manuscript.

Thank you for your submission

[Note: HTML markup is below. Please do not edit.]

Reviewers' comments:

Reviewer's Responses to Questions

**Comments to the Author**

1. Is the manuscript technically sound, and do the data support the conclusions?

Reviewer #1: Partly

Reviewer #2: Partly

**********

2. Has the statistical analysis been performed appropriately and rigorously?

Reviewer #1: N/A

Reviewer #2: No

**********

3. Have the authors made all data underlying the findings in their manuscript fully available?

The PLOS Data policy

Reviewer #1: Yes

Reviewer #2: Yes

**********

4. Is the manuscript presented in an intelligible fashion and written in standard English?

Reviewer #1: No

Reviewer #2: No

**********

Reviewer #1: This manuscript describes the effects of the AgNPs using Asplenium dalhousiae on their antibacterial, antioxidant, α-amylase inhibitory and cytotoxic activities.

General comments,

The manuscript's structure is poorly organized; the author should revise it.

The author should maintain the same font throughout the manuscript.

The author should compare their results with previous studies to provide context and validate their findings. Additionally, the discussion section needs improvement to offer a more comprehensive analysis and interpretation of the results.

For the antibacterial activity, it is insufficient for the author to work with a single concentration, as this does not provide a comprehensive assessment of the antibacterial properties. Multiple concentrations should be tested to better evaluate the activity.

The author has presented their results in Figures 1, 2, 3, etc., without citing the figure numbers in the text. The author should properly cite the figure numbers in accordance with the corresponding results for better clarity and alignment.

More detailed table and figure captions should be given for self-understood.

The resolution of the characterization figures for the AgNPs needs to be improved to enhance clarity and allow for better interpretation of the results.

Table 3, the author should include the standard deviation (SD) value for each AgNPs and ascorbic acid in the same column, rather than in a separate column

In Table 4, it is unclear why the author has provided a 'groups' column for the concentration. This should be clarified or revised for better understanding. And delete the empty box in the table.

In Table 5, instead of 'Active,' the author should mention the value of the IC50.

Detailed comments have been noted on the manuscript.

Reviewer #2: Manuscript ID: PONE-D-24-42400

Biosynthesis and Characterization of Silver Nanoparticles from Asplenium dalhousiae and their Potential Biological Properties

This study explores the feasibility of green synthesis of AgNPs using plant extracts, with antibacterial, antioxidant, and cytotoxic activities from Asplenium dalhousiae plants. Although the study emphasizes the potential biological properties of Asplenium dalhousiae, but it does not adequately justify its selection over other well-established method for synthesis of AgNPs using plant extracts. Significant efforts are required to differentiate this work from the current published literature and to claim the novelty. In addition, two kind of manuscript file submitted in one file the first one and the second one which is the better version and the following correction are suggested for the second one.

In its present form, the manuscript is not recommended for publication unless the following issues are addressed:

1. The English language and grammar in the whole manuscript needs further improvement, particularly in sections such as Introduction, Materials and Methods.

2. The statements such as “paving the way for improved treatment outcomes in patients with ovarian cancer” mentioned in the end of the Abstract are speculative without preclinical or clinical validation. Cytotoxicity is an in-vitro property that does not equate with the therapeutic efficacy for in-vivo and should be compared with literature to substantiate differences.

3. Figures are poorly presented and should be arranged into collages for better readability and interpretation.

4. The study lacks mechanistic insights, such as identifying specific compounds in Asplenium dalhousiae responsible for nanoparticle synthesis and their specific role in reduction and stabilization.

5. The α-amylase inhibition and cytotoxic mechanisms are not explained well by authors.

6. The authors claim in the introduction section that the reported work is the "first comprehensive investigation" of Asplenium dalhousiae which is unsupported with the previously published and related research. Comparison with other studies on plant-mediated AgNP synthesis is required to highlight the novelty.

7. Characterization methods such as UV-Vis, FTIR, XRD, and SEM are discussed generally, without any specific experimental optimization or justifications, that could be associated with the changes made before and after synthesis in the results obtained through these characterizations.

8. Statistical analysis is missing and biological assay results should include error bars, p-values, and measures of significance.

9. The bio-reduction process needs deeper exploration, linking phytochemicals and functional groups identified in FTIR to biological activities.

10. Implications like scalability, toxicity, and economic feasibility of Asplenium dalhousiae-based AgNPs are not well addressed by the authors.

11. Redundancy is evident, with repeated discussions on green synthesis of AgNPs’ and its biomedical roles without adding new insights.

12. Oversimplified statements such as “Nanoparticles are far more dangerous to cancerous cells than bulk materials,” these statements show lack of explanation with supporting references.

13. The antioxidant assay concentration range (10–100 µg/ml) is vague and lacks justification linked to standard protocols with particular reference.

14. Broad peaks in XRD patterns require consideration of factors like crystallanity and particle size distribution with particular strain during interpretation.

15. Missing details, such as solvent partition criteria and centrifugation rationale, hindered reproducibility.

16. The reported particle size range in SEM is of 1–100 nm is excessively broad and should include specific details like average size, standard deviation, and size distribution.

**********

what does this mean? ). If published, this will include your full peer review and any attached files.

If you choose “no”, your identity will remain anonymous but your review may still be made public.

**Do you want your identity to be public for this peer review?** For information about this choice, including consent withdrawal, please see our Privacy Policy

Reviewer #1: **Yes: ** Krishnan Raguvaran

Reviewer #2: No

**********

[NOTE: If reviewer comments were submitted as an attachment file, they will be attached to this email and accessible via the submission site. Please log into your account, locate the manuscript record, and check for the action link "View Attachments". If this link does not appear, there are no attachment files.]

While revising your submission, please upload your figure files to the Preflight Analysis and Conversion Engine (PACE) digital diagnostic tool, https://pacev2.apexcovantage.com/ . PACE helps ensure that figures meet PLOS requirements. To use PACE, you must first register as a user. Registration is free. Then, login and navigate to the UPLOAD tab, where you will find detailed instructions on how to use the tool. If you encounter any issues or have any questions when using PACE, please email PLOS at figures@plos.org . Please note that Supporting Information files do not need this step.

Attachment Submitted filename: PONE-D-24-42400_reviewer.pdf

Attachment Submitted filename: PONE-D-24-42400-Reviewer Comments-V1.docx

# Author comment: Author response to Decision Letter 1

**DOI:** 10.1371/journal.pone.0325533.r002

14 Feb 2025

PONE-D-24-42400

Biosynthesis and Characterization of Silver Nanoparticles from Asplenium dalhousiae and their Potential Biological Properties

PLOS ONE

Reviewer #1:

The effects of the AgNPs using Asplenium dalhousiae on their antibacterial, antioxidant, α-amylase inhibitory and cytotoxic activities.

General comments,

Comments Response

1. The manuscript's structure is poorly organized; the author should revise it.

I appreciate your comment regarding the manuscript's structure. I will carefully revise and reorganize the content to improve its clarity and flow, ensuring that the manuscript is more coherent and well-structured."

2. The author should maintain the same font throughout the manuscript. Thank you for the feedback; I have ensured that the font is consistent throughout the manuscript.

3. The author should compare their results with previous studies to provide context and validate their findings. Additionally, the discussion section needs improvement to offer a more comprehensive analysis and interpretation of the results. Thank you for your suggestions. I will revise the discussion section to include comparisons with previous studies and provide a more detailed analysis and interpretation of the results.

4. For the antibacterial activity, it is insufficient for the author to work with a single concentration, as this does not provide a comprehensive assessment of the antibacterial properties. Multiple concentrations should be tested to better evaluate the activity.

Thank you very much for your insightful comment. We fully understand the importance of testing multiple concentrations to provide a comprehensive assessment of antibacterial activity. In this study, we chose to evaluate the antibacterial properties at a single concentration of 30 µg/ml due to practical constraints. This concentration was selected based on preliminary experiments that indicated significant antimicrobial activity across the different extracts and AgNPs. Additionally, different plant extracts, including those from CHCl3, aqueous, and n-hexane layers, were used to represent compounds of varying polarities. The choice of 30 µg/ml was intended to provide a consistent basis for comparing the activity of compounds with distinct polarity profiles.

We agree that evaluating antibacterial activity at multiple concentrations would offer a more thorough understanding of the dose-dependent effects. We intend to explore this in future studies when additional resources allow for a more detailed investigation into concentration-dependent activity. We appreciate your feedback and hope to address this aspect in our forthcoming research.

5. The author has presented their results in Figures 1, 2, 3, etc., without citing the figure numbers in the text. The author should properly cite the figure numbers in accordance with the corresponding results for better clarity and alignment We have carefully incorporated the suggested revisions into the manuscript, and all figures have been appropriately cited within the text.

6. More detailed table and figure captions should be given for self-understood.

Thank you for highlighting the need for more detailed captions for tables and figures. We have carefully revised all captions to make them more descriptive and self-explanatory. The updated captions now provide additional context, including details about the experimental setup, variables, units, and key observations, ensuring clarity for the reader without requiring reference to the main text. We believe these changes enhance the comprehensibility of the data presented."

7. The resolution of the characterization figures for the AgNPs needs to be improved to enhance clarity and allow for better interpretation of the results.

Thank you for your insightful comment regarding the resolution of the characterization figures for the AgNPs. We have revisited the figures and improved their resolution to enhance clarity and ensure that all details are visible and interpretable. The revised figures have been exported in high-resolution formats (300 DPI) to meet publication standards. We trust that the updated images now provide better visual quality for accurate interpretation of the results."

8. Table 3, the author should include the standard deviation (SD) value for each AgNPs and ascorbic acid in the same column, rather than in a separate column

We have revised the table to combine the '% Cell Viability' and corresponding Standard Deviation (SD) values into a single column for both AgNPs and Ascorbic Acid, as suggested.

9. In Table 4, it is unclear why the author has provided a 'groups' column for the concentration. This should be clarified or revised for better understanding. And delete the empty box in the table.

Thank you for your valuable comment regarding the 'Groups' column in Table 4. After considering your feedback, we have removed the 'Groups' column to eliminate any confusion. The table now presents the concentrations directly in the first column, making it clearer and more straightforward. Additionally, we have deleted the empty box as suggested. We believe this revision improves the presentation and readability of the data.

10. In Table 5, instead of 'Active,' the author should mention the value of the IC50.

Detailed comments have been noted on the manuscript.

Suggestion has been in corporate

Reviewer #2: Manuscript ID: PONE-D-24-42400

Biosynthesis and Characterization of Silver Nanoparticles from Asplenium dalhousiae and their Potential Biological Properties

S No. Comments Response

1. The English language and grammar in the whole manuscript needs further improvement, particularly in sections such as Introduction, Materials and Methods. We will thoroughly review the manuscript and make the necessary improvements to the English language and grammar, particularly in the Introduction and Materials and Methods sections, to ensure clarity and readability

2. The statements such as “paving the way for improved treatment outcomes in patients with ovarian cancer” mentioned in the end of the Abstract are speculative without preclinical or clinical validation. Cytotoxicity is an in-vitro property that does not equate with the therapeutic efficacy for in-vivo and should be compared with literature to substantiate differences.

We will revise the Abstract to clearly indicate that the findings are based on in-vitro studies and emphasize the need for further preclinical and clinical investigations. Additionally, we will compare our cytotoxicity results with relevant literature to provide context and highlight any significant differences."

3. Figures are poorly presented and should be arranged into collages for better readability and interpretation.

We will reorganize the figures into well-structured collages to enhance their readability and facilitate better interpretation. The revised figures will be designed to ensure clarity and improve the overall presentation of the data

4. The study lacks mechanistic insights, such as identifying specific compounds in Asplenium dalhousiae responsible for nanoparticle synthesis and their specific role in reduction and stabilization.

Thank you for your valuable suggestion. We acknowledge the importance of providing mechanistic insights. While this study focuses on demonstrating the synthesis and biological activities of nanoparticles, future work will aim to identify the specific compounds in Asplenium dalhousiae responsible for nanoparticle synthesis and elucidate their role in reduction and stabilization. We will also include a discussion in the manuscript to address this limitation and highlight the need for further investigation

5. The α-amylase inhibition and cytotoxic mechanisms are not explained well by authors.

Thank you for your valuable feedback. We acknowledge the need for a more detailed explanation of the α-amylase inhibition and cytotoxic mechanisms. We have added the mechanism in the revised manuscript,

6. The authors claim in the introduction section that the reported work is the "first comprehensive investigation" of Asplenium dalhousiae which is unsupported with the previously published and related research. Comparison with other studies on plant-mediated AgNP synthesis is required to highlight the novelty.

We have revised the introduction to acknowledge prior studies on plant-mediated AgNP synthesis and added comparisons with related research to highlight the novelty of using Asplenium dalhousiae for the first time in this context.

7. Characterization methods such as UV-Vis, FTIR, XRD, and SEM are discussed generally, without any specific experimental optimization or justifications, that could be associated with the changes made before and after synthesis in the results obtained through these characterizations.

We have updated the manuscript to include specific details on the experimental optimizations and justifications for using UV-Vis, FTIR, XRD, and SEM methods. Additionally, we have elaborated on the observed changes before and after synthesis, linking them to the results obtained from these characterizations.

8. Statistical analysis is missing and biological assay results should include error bars, p-values, and measures of significance.

We have included statistical analysis in the revised manuscript, incorporating error bars, p-values, and measures of significance for the biological assay results to ensure accurate interpretation and validation of the data.

9. The bio-reduction process needs deeper exploration, linking phytochemicals and functional groups identified in FTIR to biological activities. We have expanded the discussion on the bio-reduction process by linking the phytochemicals and functional groups identified in FTIR to their potential roles in the reduction and stabilization of AgNPs, as well as their contribution to the observed biological activities.

10. Implications like scalability, toxicity, and economic feasibility of Asplenium dalhousiae-based AgNPs are not well addressed by the authors. The scalability of Asplenium dalhousiae-based AgNPs production is achievable due to the abundance of the plant material and the simplicity of the green synthesis method, which avoids the use of expensive or hazardous chemicals. In terms of toxicity, the biocompatibility of the plant-derived nanoparticles has been discussed in the context of their reduced environmental impact and selective bioactivity, supported by their antioxidant and antibacterial properties. Additionally, the economic feasibility is highlighted by the low-cost extraction process and the use of renewable plant resources, making it a sustainable and cost-efficient alternative for nanoparticle synthesis. These aspects emphasize the practical advantages and potential for commercial application of the reported method.

11. Redundancy is evident, with repeated discussions on green synthesis of AgNPs’ and its biomedical roles without adding new insights.

We acknowledge the comment regarding redundancy in the discussion of green synthesis and biomedical roles of AgNPs. To address this, we have carefully revised the manuscript by consolidating repetitive content and emphasizing unique aspects of our study, such as the distinct contribution of Asplenium dalhousiae phytochemicals in nanoparticle synthesis and their specific biological activities. This revision ensures clarity and avoids unnecessary repetition.

12. Oversimplified statements such as “Nanoparticles are far more dangerous to cancerous cells than bulk materials,” these statements show lack of explanation with supporting references.

I agree that the statement lacks sufficient explanation and supporting references. The enhanced efficacy of nanoparticles over bulk materials, particularly in the context of cancer treatment, can be attributed to their unique physicochemical properties such as a high surface area-to-volume ratio, the ability to penetrate biological barriers, and targeted drug delivery. I will revise the statement to include these details and references to provide a more thorough explanation.

13. The antioxidant assay concentration range (10–100 µg/ml) is vague and lacks justification linked to standard protocols with particular reference.

14. Broad peaks in XRD patterns require consideration of factors like crystallanity and particle size distribution with particular strain during interpretation.

15. Missing details, such as solvent partition criteria and centrifugation rationale, hindered reproducibility.

16. The reported particle size range in SEM is of 1–100 nm is excessively broad and should include specific details like average size, standard deviation, and size distribution.

Attachment Submitted filename: Comments paper highlighted comments.docx

# Peer review: Decision Letter 1

**DOI:** 10.1371/journal.pone.0325533.r003

**Authors:** Chinnaperumal Kamaraj

Dear Dr. maher,

Thank you for submitting your manuscript to PLOS ONE. After careful consideration, we feel that it has merit but does not fully meet PLOS ONE’s publication criteria as it currently stands. Therefore, we invite you to submit a revised version of the manuscript that addresses the points raised during the review process.

==============================

Dear Dr. Saima Maher,

Thank you for submitting your manuscript titled "Biosynthesis and Characterization of Silver Nanoparticles from Asplenium dalhousiae and their Potential Biological Properties" to PLOS ONE. After a comprehensive review, we are happy to inform you that your manuscript is nearly ready for acceptance. However, in light of the reviewer feedback, a few minor revisions are necessary before we can proceed with finalizing the publication.

==============================

Please submit your revised manuscript by May 01 2025 11:59PM. If you will need more time than this to complete your revisions, please reply to this message or contact the journal office at plosone@plos.org . When you're ready to submit your revision, log on to https://www.editorialmanager.com/pone/ and select the 'Submissions Needing Revision' folder to locate your manuscript file.

- A rebuttal letter that responds to each point raised by the academic editor and reviewer(s). You should upload this letter as a separate file labeled 'Response to Reviewers'.
- A marked-up copy of your manuscript that highlights changes made to the original version. You should upload this as a separate file labeled 'Revised Manuscript with Track Changes'.
- An unmarked version of your revised paper without tracked changes. You should upload this as a separate file labeled 'Manuscript'.

If applicable, we recommend that you deposit your laboratory protocols in protocols.io to enhance the reproducibility of your results. Protocols.io assigns your protocol its own identifier (DOI) so that it can be cited independently in the future. For instructions see: https://journals.plos.org/plosone/s/submission-guidelines#loc-laboratory-protocols . Additionally, PLOS ONE offers an option for publishing peer-reviewed Lab Protocol articles, which describe protocols hosted on protocols.io. Read more information on sharing protocols at https://plos.org/protocols?utm_medium=editorial-email&utm_source=authorletters&utm_campaign=protocols .

We look forward to receiving your revised manuscript.

Kind regards,

Chinnaperumal Kamaraj, Ph.D

Academic Editor

PLOS ONE

Journal Requirements:

Please review your reference list to ensure that it is complete and correct. If you have cited papers that have been retracted, please include the rationale for doing so in the manuscript text, or remove these references and replace them with relevant current references. Any changes to the reference list should be mentioned in the rebuttal letter that accompanies your revised manuscript. If you need to cite a retracted article, indicate the article’s retracted status in the References list and also include a citation and full reference for the retraction notice.

Additional Editor Comments:

Dear Dr. Saima Maher,

Thank you for submitting your manuscript titled "Biosynthesis and Characterization of Silver Nanoparticles from Asplenium dalhousiae and their Potential Biological Properties" to PLOS ONE. After a comprehensive review, we are happy to inform you that your manuscript is nearly ready for acceptance. However, in light of the reviewer's feedback, a few minor revisions are necessary before we can proceed with finalizing the publication.

[Note: HTML markup is below. Please do not edit.]

Reviewers' comments:

Reviewer's Responses to Questions

**Comments to the Author**

Reviewer #1: All comments have been addressed

Reviewer #3: All comments have been addressed

**********

2. Is the manuscript technically sound, and do the data support the conclusions?

Reviewer #1: Yes

Reviewer #3: Yes

**********

3. Has the statistical analysis been performed appropriately and rigorously?

Reviewer #1: Yes

Reviewer #3: Yes

**********

4. Have the authors made all data underlying the findings in their manuscript fully available?

The PLOS Data policy

Reviewer #1: Yes

Reviewer #3: Yes

**********

5. Is the manuscript presented in an intelligible fashion and written in standard English?

Reviewer #1: Yes

Reviewer #3: Yes

**********

Reviewer #1: This study prepared silver nanoparticles (Ag-NPs) synthesized using Asplenium dalhousiae, which demonstrated significant antibacterial, antioxidant, α-amylase inhibitory, and anticancer activity. However, there is still room for improvement in writing quality, graphical representation, and the overall content of the manuscript. Therefore, I believe this manuscript is acceptable for publication in PLOS ONE after minor revisions.

1. In the abstract, the author initially mentioned that antibacterial activity was tested against Bacillus subtilis and Escherichia coli. However, this information does not match the results section. The author should ensure consistency and correct this based on the original experiment.

2. The author should clarify whether plant extracts (aqueous, chloroform, and n-hexane) were obtained through natural dissolution and provide a more detailed explanation of the extraction process using different solvents.

3. The UV-Vis spectroscopy section should be rewritten separately, with some portions moved to the Results and Discussion section.

4. Additionally, the author should provide details of the instruments used, including the make and manufacturer, for all technical analyses.

5. The manuscript contains several typographical errors (e.g., punctuation mistakes in periods, commas, superscripts, and subscripts) and grammatical issues. These should be corrected, and the author should ensure a smooth flow of sentences.

6. Preparation of Asplenium dalhousiae Extract and AgNPs: The term "AgNPs water" is unclear and should be clarified.

7. The methodology for the preparation of Asplenium dalhousiae extract and AgNPs synthesis is confusing and should be revised for better clarity.

8. The Statistical Analysis section should be placed at the end of the Methods section.

9. Some words in the manuscript are bolded without a clear reason. The author should clarify if there is a specific purpose for this formatting.

10. During AgNP synthesis, the observed color change is inconsistent. Some sections describe a transition from colorless to reddish-brown, while others mention yellow to orange-brown. The author should carefully revise the manuscript to ensure consistency in experimental results and methods.

11. Figure 3 (FTIR Spectrum of Asplenium dalhousiae Plant Extract): The legend describes FTIR analysis of aqueous, chloroform, and n-hexane extracts. If the results refer to AgNPs rather than plant extracts, the legend should be revised accordingly.

12. Figure 4 (XRD Analysis of AgNPs): The author should maintain consistency in terminology when referring to plant extracts or layers.

13. The figure and legend should be revised for uniformity.

14. Table 1 (Antibacterial Activity of Silver Nanoparticles and Plant Extracts): The mean values and dose figures should be formatted uniformly.

15. The author should verify whether Figure 7 represents the antidiabetic test results.

16. All figure legends should be revised, as they do not accurately describe the graphical data. The author is requested to use appropriate legends for each test result.

Reviewer #3: Author did a excellent job in nanomaterials and biomedical application. The author has addressed all comments. Now accepted to publication

**********

what does this mean? ). If published, this will include your full peer review and any attached files.

If you choose “no”, your identity will remain anonymous but your review may still be made public.

**Do you want your identity to be public for this peer review?** For information about this choice, including consent withdrawal, please see our Privacy Policy

Reviewer #1: No

Reviewer #3: No

**********

[NOTE: If reviewer comments were submitted as an attachment file, they will be attached to this email and accessible via the submission site. Please log into your account, locate the manuscript record, and check for the action link "View Attachments". If this link does not appear, there are no attachment files.]

While revising your submission, please upload your figure files to the Preflight Analysis and Conversion Engine (PACE) digital diagnostic tool, https://pacev2.apexcovantage.com/ . PACE helps ensure that figures meet PLOS requirements. To use PACE, you must first register as a user. Registration is free. Then, login and navigate to the UPLOAD tab, where you will find detailed instructions on how to use the tool. If you encounter any issues or have any questions when using PACE, please email PLOS at figures@plos.org

Attachment Submitted filename: Comments.docx

# Author comment: Author response to Decision Letter 2

**DOI:** 10.1371/journal.pone.0325533.r004

1 May 2025

Dear Chinnaperumal Kamaraj, Ph.D Date: 22/04/2025

Editor

PLOS ONE

Subject: Resubmission of Manuscript (PONE-D-24-42400R1)

Dear Dr. Kamaraj,

Thank you for the opportunity to revise and resubmit our manuscript titled "Biosynthesis and Characterization of Silver Nanoparticles from Asplenium dalhousiae and their Potential Biological Properties" (PONE-D-24-42400R1). We greatly appreciate the feedback from both you and the reviewers, which has been extremely helpful in improving the quality of the manuscript. We have carefully addressed all the comments raised, as outlined below.

Reviewer #1:

1. Comment: In the abstract, the author initially mentioned that antibacterial activity was tested against Bacillus subtilis and Escherichia coli. However, this information does not match the results section. The author should ensure consistency and correct this based on the original experiment.

Response: Thank you for highlighting this discrepancy. We have carefully reviewed the manuscript and ensured that the antibacterial activity results are consistent across the abstract and results sections. The correct bacterial strains tested are now consistently mentioned as Bacillus subtilis and Escherichia coli.

2. Comment: The author should clarify whether plant extracts (aqueous, chloroform, and n-hexane) were obtained through natural dissolution and provide a more detailed explanation of the extraction process using different solvents.

Response: We have revised the Materials and Methods section to clarify the extraction process of the plant material. We now provide a more detailed explanation of the solvent extraction procedure, specifying the methods used for aqueous, chloroform, and n-hexane extracts.

3. Comment: The UV-Vis spectroscopy section should be rewritten separately, with some portions moved to the Results and Discussion section.

Response: We have revised the manuscript as requested. The UV-Vis spectroscopy section has been separated and rewritten with a focus on the results and discussion of the data in the Results and Discussion section. The experimental details have been moved to the Materials and Methods section.

4. Comment: The author should provide details of the instruments used, including the make and manufacturer, for all technical analyses.

Response: We have updated the manuscript to include detailed information about the instruments used for each technical analysis, including the make and manufacturer, as suggested.

5. Comment: The manuscript contains several typographical errors (e.g., punctuation mistakes in periods, commas, superscripts, and subscripts) and grammatical issues. These should be corrected, and the author should ensure a smooth flow of sentences.

Response: We appreciate this observation. We have thoroughly proofread the manuscript to correct typographical and grammatical errors, including issues with punctuation, superscripts, and subscripts. The flow of sentences has also been improved for better readability.

6. Comment: The term “AgNPs water” is unclear and should be clarified.

Response: The term “AgNPs water” has been revised for clarity. We now refer to it as “aqueous dispersion of AgNPs” throughout the manuscript to avoid any confusion.

7. Comment: The methodology for the preparation of Asplenium dalhousiae extract and AgNPs synthesis is confusing and should be revised for better clarity.

Response: We have revised the Materials and Methods section to clarify the synthesis of AgNPs from Asplenium dalhousiae extracts. The revised description now provides a clearer step-by-step process for both the plant extract preparation and nanoparticle synthesis.

8. Comment: The Statistical Analysis section should be placed at the end of the Methods section.

Response: We have moved the Statistical Analysis section to the end of the Materials and Methods section, as suggested, to align with the manuscript’s structure.

9. Comment: Some words in the manuscript are bolded without a clear reason. The author should clarify if there is a specific purpose for this formatting.

Response: We have reviewed the manuscript and removed unnecessary bolding. Any remaining bolded words now serve a clear purpose for emphasis or as part of section headings.

10. Comment: During AgNP synthesis, the observed color change is inconsistent. Some sections describe a transition from colorless to reddish-brown, while others mention yellow to orange-brown. The author should carefully revise the manuscript to ensure consistency in experimental results and methods.

Response: We have carefully reviewed and revised the description of the color change during AgNP synthesis. The manuscript now consistently describes the color change as “colorless to reddish-brown,” which aligns with the observed experimental results.

11. Comment: Figure 3 (FTIR Spectrum of Asplenium dalhousiae Plant Extract): The legend describes FTIR analysis of aqueous, chloroform, and n-hexane extracts. If the results refer to AgNPs rather than plant extracts, the legend should be revised accordingly.

Response: We have revised the legend of Figure 3 to clearly specify that the FTIR analysis was conducted on the Asplenium dalhousiae plant extracts. Any mention of AgNPs in the legend has been removed for accuracy.

12. Comment: Figure 4 (XRD Analysis of AgNPs): The author should maintain consistency in terminology when referring to plant extracts or layers.

Response: We have ensured consistency in terminology in the Results and Figures sections. We now consistently refer to the AgNPs and plant extracts separately, with clear distinctions between the two.

13. Comment: The figure and legend should be revised for uniformity.

Response: We have revised the figures and their legends to ensure uniformity in presentation. All figure formatting and legends now follow the same structure for consistency.

14. Comment: Table 1 (Antibacterial Activity of Silver Nanoparticles and Plant Extracts): The mean values and dose figures should be formatted uniformly.

Response: We have updated Table 1 to ensure uniform formatting of the mean values and dose figures, ensuring consistency throughout the manuscript.

15. Comment: The author should verify whether Figure 7 represents the antidiabetic test results.

Response: We have reviewed Figure 7, and we confirm that it represents the anti-diabetic test results. The legend has been updated to ensure that it accurately reflects this data.

16. Comment: All figure legends should be revised, as they do not accurately describe the graphical data. The author is requested to use appropriate legends for each test result.

Response: We have carefully revised all figure legends to ensure they accurately describe the graphical data. Each legend now clearly corresponds to the respective test results (e.g., antibacterial, antioxidant, anti-diabetic activities).

We sincerely appreciate the valuable comments from the reviewers, which have significantly improved the manuscript. We believe that the revisions have addressed all concerns raised, and we are confident that the revised manuscript is now suitable for publication. We look forward to your favorable consideration of our revised submission.

Thank you once again for your time and consideration.

Sincerely,

Dr. Saima Mehar

SBK Women University

Attachment Submitted filename: response to reviewer.docx

# Editor report: Decision Letter 2

**DOI:** 10.1371/journal.pone.0325533.r005

**Authors:** Chinnaperumal Kamaraj

Biosynthesis and Characterization of Silver Nanoparticles from Asplenium dalhousiae and their Potential Biological Properties

PONE-D-24-42400R2

Dear Dr. maher,

We’re pleased to inform you that your manuscript has been judged scientifically suitable for publication and will be formally accepted for publication once it meets all outstanding technical requirements.

Within one week, you’ll receive an e-mail detailing the required amendments. When these have been addressed, you’ll receive a formal acceptance letter and your manuscript will be scheduled for publication.

An invoice will be generated when your article is formally accepted. Please note, if your institution has a publishing partnership with PLOS and your article meets the relevant criteria, all or part of your publication costs will be covered. Please make sure your user information is up-to-date by logging into Editorial Manager at Editorial Manager®  and clicking the ‘Update My Information' link at the top of the page. If you have any questions relating to publication charges, please contact our Author Billing department directly at authorbilling@plos.org.

If your institution or institutions have a press office, please notify them about your upcoming paper to help maximize its impact. If they’ll be preparing press materials, please inform our press team as soon as possible -- no later than 48 hours after receiving the formal acceptance. Your manuscript will remain under strict press embargo until 2 pm Eastern Time on the date of publication. For more information, please contact onepress@plos.org.

Kind regards,

Chinnaperumal Kamaraj, Ph.D

Academic Editor

PLOS ONE

Additional Editor Comments (optional):

Reviewers' comments:

# Editor report: Acceptance letter

**DOI:** 10.1371/journal.pone.0325533.r006

**Authors:** Chinnaperumal Kamaraj

PONE-D-24-42400R2

PLOS ONE

Dear Dr. maher,

I'm pleased to inform you that your manuscript has been deemed suitable for publication in PLOS ONE. Congratulations! Your manuscript is now being handed over to our production team.

At this stage, our production department will prepare your paper for publication. This includes ensuring the following:

* All references, tables, and figures are properly cited

* All relevant supporting information is included in the manuscript submission,

* There are no issues that prevent the paper from being properly typeset

You will receive further instructions from the production team, including instructions on how to review your proof when it is ready. Please keep in mind that we are working through a large volume of accepted articles, so please give us a few days to review your paper and let you know the next and final steps.

Lastly, if your institution or institutions have a press office, please let them know about your upcoming paper now to help maximize its impact. If they'll be preparing press materials, please inform our press team within the next 48 hours. Your manuscript will remain under strict press embargo until 2 pm Eastern Time on the date of publication. For more information, please contact onepress@plos.org.

If we can help with anything else, please email us at customercare@plos.org.

Thank you for submitting your work to PLOS ONE and supporting open access.

Kind regards,

PLOS ONE Editorial Office Staff

on behalf of

Dr. Chinnaperumal Kamaraj

Academic Editor

PLOS ONE
